# Urban health advantage and penalty in aging populations: a comparative study across major megacities in China

**DOI:** 10.1016/j.lanwpc.2024.101112

**Published:** 2024-06-15

**Authors:** Jialu Song, Linxin Liu, Hui Miao, Yanjie Xia, Dong Li, Jun Yang, Haidong Kan, Yi Zeng, John S. Ji

**Affiliations:** a Vanke School of Public Health, Tsinghua University, Beijing, China; b School of Public Health, Peking University, Beijing, China; c T.H. Chan School of Public Health, Harvard University, Boston, MA, USA; d Institute for Urban Governance and Sustainable Development, Tsinghua University, Beijing, China; e Department of Earth System Science, Institute for Global Change Studies, Tsinghua University, Beijing, China; f School of Public Health, Fudan University, Shanghai, China; g National School of Development, Peking University, Beijing, China; h School of Medicine, Duke University, Durham, NC, USA

**Keywords:** Epidemiology, Healthy city, China, Air pollution, Green space, Commercial determinants of health, Aging

## Abstract

**Background:**

Urban living is linked to better health outcomes due to a combination of enhanced access to healthcare, transportation, and human development opportunities. However, spatial inequalities lead to disparities, resulting in urban health advantages and penalties. Understanding the relationship between health and urban development is needed to generate empirical evidence in promoting healthy aging populations. This study provides a comparative analysis using epidemiological evidence across diverse major Chinese cities, examining how their unique urban development trajectories over time have impacted the health of their aging residents.

**Methods:**

We tracked changes in air pollution (NO_2_, PM_2.5_, O_3_), green space (measured by NDVI), road infrastructure (ring road areas), and nighttime lighting over 20 years in six major cities in China. We followed a longitudinal cohort of 4992 elderly participants (average age 87.8 years) over 16,824 person-years. We employed Cox proportional hazard regression to assess longevity, assessing 14 variables, including age, sex, ethnicity, marital status, residence, household income, occupation, education, smoking, alcohol consumption, exercise, and points of interest (POI) count of medicine-related facilities, sports, and leisure service-related places, and scenic spots within a 5 km-radius buffer.

**Findings:**

Geographic proximity to points of interest significantly improves survival. Elderly living in proximity of the POI-rich areas had a 34.6%–35.6% lower mortality risk compared to those in POI-poor areas, for the highest compared to the lowest quartile. However, POI-rich areas had higher air pollution levels, including PM_2.5_ and NO_2_, which was associated with a 21% and 10% increase in mortality risk for increase of 10 μg/m^3^, respectively. The benefits of urban living had higher effect estimates in monocentric cities, with clearly defined central areas, compared to polycentric layouts, with multiple satellite city centers.

**Interpretation:**

Spatial inequalities create urban health advantages for some and penalties for others. Proximity to public facilities and economic activities is associated with health benefits, and may counterbalance the negative health impacts of lower green space and higher air pollution. Our empirical evidence show optimal health gains for age-friendly urban environments come from a balance of infrastructure, points of interest, green spaces, and low air pollution.

**Funding:**

Natural Science Foundation of Beijing (IS23105), National Natural Science Foundation of China (82250610230, 72061137004), World Health Organization (2024/1463606-0), Research Fund Vanke School of Public Health Tsinghua University (2024JC002), Beijing TaiKang YiCai Public Welfare Foundation, National Key R&D Program of China (2018YFC2000400).

**Research in context**

**Evidence before this study**

We searched PubMed, CNKI, and Google Scholar for the studies on urban health published in English up to October 2023. We used a combination of search terms, including “city,” “urban” “urban planning,” “environmental impact,” “health inequalities,” and “healthy aging.” Previous studies have documented the health advantages of urban living due to the enhanced accessibility of healthcare, education resources, and transportation. In developed countries, urban areas have experienced decentralization, leading to concentrations of poverty, crime, and drug use in city centers, which consequently resulted in poor health. Limited attention was paid to health disparities within different city areas in China and their association with environmental impact.

**Added value of this study**

Our study uses high-resolution geospatial demographic data to explore the complex interaction between the urban environment and urban planning with individual-level health outcomes in aging populations within megacities in China. We assessed empirical evidence showing inequities of resources and pollution within cities and between cities. We found that residents in city centers enjoy substantial health benefits from proximity and access to public facilities and economic activities, these factors are collectively associated with healthy aging, and the advantages of the social environment seem to offset the detrimental impacts of reduced green space and heightened air pollution in central urban areas and even exceed the latter in cities with more monocentric layouts.

**Implications of all the available evidence**

The findings of our study are instrumental in aiding urban planners and health policymakers to promote polycentric city layouts and construct more equitable, age-friendly cities. Furthermore, our research offers novel insights into the current industrial layout adjustments that this initiative, to some extent, might be diminishing the limited health advantage of living in the city center. We found evidence that the urban environment is an indispensable factor in health inequalities.

## Introduction

Cities serve as hubs of improved infrastructure and services, historically made the earliest advancements in sanitation, water quality, nutrition, healthcare access, education attainment, and is a driver of economic growth. However, urban environments are also associated with pollution, overcrowding, and health inequalities.^1^, ^2^, ^3^, ^4^ China has undergone an unprecedented urbanization process, with over 600 million people migrating from rural areas to cities, resulting in many megacities with populations exceeding 10 million inhabitants.^5^^,^^6^ However, within the country, there is significant heterogeneity in life expectancy. For example, residents of Shanghai had an average life expectancy of 83.2 years in 2022, which is on par with or even exceeds that of many developed countries in the Organization for Economic Co-operation and Development (OECD), and other less developed western regions have life expectancy of around 70 years.^7^^,^^8^ Aside from the eastern coastal and western inland life expectancy gap, there is also an urban-rural life expectancy gap, with a noticeable life expectancy gap within China, with city residents living on average seven years longer than their rural counterparts.^5^ Many hypotheses attributable this to sociodemographic factors such as education, medical care, and retirement benefits.^9^ Nevertheless, the health benefits of urban living have been diminishing in high-income countries.^10^ This might be attributable to the fact that megacities were linked with unstable sources of food, increasing violence, poor dietary and lifestyle habits, and air pollution.^11^ A higher proportion of elderly residents now live in cities, and understanding urban health advantages and urban health penalties can preserve and aid in furthering life expectancy gains in the future.

China's megacities have undergone rapid growth, economic specialization, and developed distinct characteristics. Shanghai, as a coastal city, has become a major financial hub, Beijing is known as the political and cultural capital, and Guangzhou, situated in the Pearl River Delta, has emerged as a center for international trade and transportation.^12^ Varying urbanization processes have led to different city layouts. Monocentric planning focuses on developing a single central hub, while polycentric planning creates multiple centers with similar access to services and amenities. Monocentric models, although favored for resource efficiency and accessibility, face challenges such as employment congestion, traffic, and environmental issues due to the concentration of urban functions.^13^ Cities in the Yangtze River Delta and the Pearl River Delta evolved a polycentric spatial structure [13Most prior studies focus on urban-rural disparities, with limited attention to evidence-based health inequalities within cities. Health inequalities within cities are due to factors like water resource management, air and noise pollution, green space, and housing quality.^14^ Globally, urban decentralization has, in some developed economies, led to pockets of poverty, crime, and drug use in certain urban areas.^15^^,^^16^ Conversely, in BRICS countries, inner cities are desirable due to better access to infrastructure, employment, and transportation.^17^ The urbanization process in China, distinct from global patterns, may impact aging uniquely and require further exploration.

We conducted a study using a population cohort in six megacities using spatiotemporal variations in green space, air pollution, nighttime light as a proxy indicator of economic activities or light pollution and environmental factors within a city on an ecological and individual level.^18^, ^19^, ^20^ First, we hypothesize that city centers confer higher health benefits, particularly in cities with strong monocentric characteristics.^21^ Second, we propose that a lack of green space and higher air pollutants may modify or negate these positive health gains. Third, we compare the relative risks of these factors on survival.

## Methods

### Ecological and health data of megacities

We obtained the Normalized Difference Vegetation Index (NDVI), particulate matters with an aerodynamic diameter smaller than 2.5 μm (PM_2.5_), and nitrogen dioxide (NO_2_) data at the ecological level and on an individual-level data. At the ecological level, we transformed the annual average NDVI into three-year moving average values. This is estimated as the average of the NDVI observed in the given incident year, the preceding year and the following year, in each megacity and in each area divided by ring roads from 2001 to 2020 by using NDVI data from February 2000 to December 2021. We also calculated 3-year moving average annual PM_2.5_ concentration from 1999 to 2019 and NO_2_ concentration from 2006 to 2019 in each megacity. Furthermore, we estimated the average NDVI from 2000 to 2021, average PM_2.5_ from 1998 to 2020 and average NO_2_ in 2000 and 2005–2020 in each area divided by ring roads to explore the spatial disparity of the above environment indicators. In the epidemiological cohort, we used five waves (2000, 2002, 2005, 2008, and 2011) from the Chinese Longitudinal Healthy Longevity Survey (CLHLS), a prospective cohort of oldest-old adults in China. The CLHLS cohort contains participants from select locations in China. A more detailed description of the cohort can be found in prior protocols.^22^ In our study, participants at advanced ages of megacities of China with a sample size of 4992 individuals. We also ascertained all-cause mortality occurring between 2000 and 2019 in the participants.

### Environmental factor changes

#### Green space

We used the NDVI, a satellite image-based vegetation index, to measure greenness. This measurement is based on chlorophyll in plants that absorb visible light for photosynthesis, and leaves reflect near-infrared light. NDVI was calculated as the ratio of the difference between the near-infrared region and the red visible reflectance to the sum of these two measurements. The NDVI value ranges from −1.0 to 1.0, with the positive value indicating vegetation coverage and the higher value indicating higher levels of vegetative density, and a negative NDVI value is often considered as blue space or water.^23^ NDVI data was obtained from the Moderate Resolution Imaging Spectroradiometer (MODIS) in the National Aeronautics and Space Administration (NASA)'s Terra satellite, which is updated every 16 days with a spatial resolution of 250 m.^24^

According to prior research, walking distance can range from 800 m (0.5 miles) to 1600 m (1 mile) and the average self-reported walking distance was found to be 0.7 miles (1126 m).^25^ Therefore, we used 1250 m buffer as a measure of greenness within the neighborhood walking distance from the residence. The cumulative annual NDVI was the mean of the annual-average NDVI during each participants' follow-up period--from the baseline year to the death year for deceased individuals, and to the last interview year for those still alive at follow-up and those lost to follow-up. We also calculated changes in annual NDVI in the 1250 m buffer by putting every year's annual-average NDVI over each participant's follow-up period into a linear regression model. The changes in annual NDVI for one participant was defined as a significant increase or decrease if the regression coefficient was positive or negative, with its P-value less than 0.05. On the contrary, if the P value was larger than 0.05, the changes in annual NDVI was defined as non-significant.

#### Air pollution

We acquired ground-level PM_2.5_ for 1998–2020 from the Atmospheric Composition Analysis Group, which combined Aerosol Optical Depth (AOD) retrievals from the NASA MODIS, Multiangle Imaging Spectro Radiometer (MISR), and Sea-viewing Wide Field-of-view instruments with the GEOS-Chem chemical transport model, and subsequently calibrating to global ground-based observations using a Geographically Weighted Regression (GWR).^26^^,^^27^ The NO_2_ data in 2000 and 2005–2020 was obtained from a global NO_2_ land use regression (LUR) model created by Larkin and colleagues, with surface annual average NO_2_ concentrations at 0.0083° (∼1 km^2^) resolution.^28^ We calculated the monthly mean ozone (O_3_) concentrations from January 2005 to December 2019 at a 1 km × 1 km spatial resolution by combining ground ozone measurements from over 1600 ground monitoring stations of the National Air Quality Monitoring Network in mainland China, ozone simulations from the Community Multiscale Air Quality (CMAQ) modeling system, meteorological parameters, population density, road length, and elevation to predict ground maximum daily 8-h average (MDA8) ozone concentrations at a daily level and. Hourly ozone concentrations were available from the China National Environmental Monitoring Center (CNEMC) since 2013.^29^ More details about the measurements of ozone concentration were described elsewhere.^30^

We linked PM_2.5,_ NO_2_ and ozone data with area-level location information. We considered the exposure window for PM_2.5,_ NO_2_ and ozone as the annual-average value in the death year for deceased individuals, and in the last interview year for those still alive at follow-up and those lost to follow-up. As NO_2_ data during 2001–2004 were unavailable, the NO_2_ concentration in 2000 was used as the last-year NO_2_ for participants whose end-up year was 2001 or 2002 and NO_2_ concentration in 2005 was used as the last-year NO_2_ for those with end-up year 2003 or 2004. For participants that lived in areas with no significant PM_2.5_ change over time, we evaluated whether PM_2.5_ showed an inverse U-shaped association with time by putting annual PM_2.5_ over each participant's follow-up period into regression models including linear and quadratic terms. The changes in annual PM_2.5_ for one participant was defined as following an inverse U shape if the regression model presents a significant positive linear (P value < 0.05 and coefficient >0) and negative quadratic (P value < 0.05 and coefficient <0) effect. Changes in individual-level annual NO_2_ and ozone were defined as a significant decrease, a non-significant change, a significant increase or an inverse U shape, in the same way as defining changes in PM_2.5_.

#### Points of interest

Data of facilities was obtained from AutoNavi Map in 2019 for each city on an ecological level.^31^ We included public facilities hypothesized to be associated with health and extracted 13 categories of POI (point of interest), including medicine-related facilities, sports and leisure service-related places, scenic spots-related places, highway-affiliated facilities, communal facilities, incorporated-business related places, shopping-related places, traffic service-related facilities, indoor facilities, living service-related places, scientific, technological, educational and cultural service-related facilities and serviced apartment-related places. We adopted a multi-dimensional approach that combines both proximity and density of POI. We computed the counts of POI within 1 km and 5 km radius. This accounts for the density of facilities within proximity, which approximately takes 20 min by walk and bus respectively. Our Cox models included the counts of three main categories of POI in 5 km radius for health-medicine-related facilities, sports and leisure service-related places and scenic spots-related places.

In this study, ring roads are used as a measure of proximity to city centers. Beijing is divided into six areas based on its five ring roads. Similarly, Shanghai, Tianjin, Chongqing, and Guangzhou are divided into five, six, four, and three areas respectively, based on the number of ring roads each city has. Chengdu, which has seven ring roads, is divided into eight areas. Geographic distribution of participants was estimated, which allows for a detailed analysis of urban health dynamics in relation to the proximity to city centers.

#### Covariates

In our analysis, we used demographic, socioeconomic and behavioral information interviewed in the baseline survey after participants entered the cohort, as covariates, including age (continuous), sex, ethnicity, education, marital status, occupation, city, living in urban or rural areas, annual household income, smoking status, alcohol consumption and exercise. We calculated continuous age by subtracting the self-reported date of birth from the date of interview, which was verified by family members, genealogical recodes, ID cards, and household registration booklets. For those reported to be older than 105 years, the age data was verified by local government committees. Meanwhile, we divided the age of participants into four categories, “65–79 years”, “80–89 years”, “90–99 years” and “≥100 years”. We classified ethnicity into two categories--Han Chinese and ethnic minority (Hui, Korean, Manchurian, Mongolian, Yao, Zhuang, and others). Education level was divided into three categories, “0 year”, “1–6 years”, and “> 6 years”. A binary variable was generated to assess marital status, “married and living with spouse”, and “not married or not living with spouse” (separated, divorced, widowed, or never married). Occupation was classified into two types: non-manual, including professional, technical, governmental, institutional or managerial personnel, and manual, including commercial, service, industrial, agriculture, forestry, animal husbandry, military or fishery personnel, self-employed, houseworker, never worked and others. City included Beijing, Shanghai, Tianjin, Guangzhou, Chongqing and Chengdu. We dichotomized location as “urban areas” and “rural areas” on the basis of the administrative division of each city. Total annual household income included three categories, “<5000 yuan”， “5000–15000 yuan”, “>15,000 yuan”. Smoking status was coded as “current smoker”, “former smoker”, or “never smoking”. A similar approach was taken to define alcohol consumption and exercise status.

#### Nighttime light

We used nighttime light as an indicator of economic activity. Ecological-level nighttime light data was obtained from Version 4 of the US Air Force Defense Meteorological Program (DMSP) Operational Line-Scan System (OLS),^32^ a cloud-free annual composited product that detects visible and near-infrared (VNIR) emission sources at night and collects all the available archived DMSP-OLS smooth resolution data for calendar years from 1992 to 2013. We used the stable light datasets from DMSP-OLS that are composited cleaned up average visible band digital number values containing the lights from cities, towns, and other sites with fires excluded.^33^ Data values range from 0 to 63. The products are 30 arc-second grids, spanning −180 to 180° longitude and −65 to 75° latitude. We used Google Earth Engine to visualize average nighttime light distribution from 2000 to 2011 since we used CLHLS cohort data during this period. We acquired individual-level nighttime light from an annual data set on night light in China Resource and Environment Science and Data Center and linked it within 1 km of participants.^34^ The dataset is based on DMSP-OLS data from 1992 to 2013 and NPP-VIIRS satellite nighttime light remote sensing image data from 2012 to now, processing and generating the annual nighttime light data of China since 1992.

#### Population density

We obtained population data from The Gridded Population of World Version 4 (GPWv4), a minimally modeled global population dataset that uniformly distributes census data into 30 arc-second (approximately 1 km) grid cells for the years 2000, 2005, 2010, 2015, and 2020. The population is allocated to cells through proportional allocation based on census and administrative units. Population input data are collected from the results of the 2010 round of censuses, which took place over the years 2005–2014, and then extrapolated to generate population estimates for each modeled year.^35^ We drew population density maps by using Google Earth Engine to demonstrate city centers and sub-centers in each city.

### Statistical analysis

Firstly, we performed an ecological analysis, describing the trends of annual NDVI, PM_2.5,_ and NO_2_ over time in each city as well as assessing the difference level of these environmental measurements among ring road areas. Area-level ozone is not included here because of data availability. Following, we used an epidemiological analysis was conducted. For baseline characteristics of participants, demographic information, socioeconomic characteristics, and lifestyle were presented as mean (continuous variables) and SD or frequency distribution (categorical variable) by cities. Greenness levels, air pollutants, and public facilities were presented by participants’ characteristics and ring road areas.

We used the Cox proportional hazard model to estimate the mortality hazard ratios to assess the relative risk of socioeconomic and environmental factors on survival. Age-sex-adjusted models were built to evaluate the association between all-cause mortality and covariates, public facilities, road, green space, air pollution. Furthermore, we constructed seven regression models, with successive variable inclusions, to assess all-cause mortality. In Model 1, we only included continuous age and sex. Model 2 was further adjusted for all covariates that could be potential confounders or predictors of mortality: ethnicity, education, occupation, marital status, annual household income, smoking status, alcohol consumption, and exercise. In Model 3, we added the counts of three main POIs within a 5 km radius based on Model 2. In Models 4 and 6, cumulative annual NDVI and trends of NDVI were included as one independent variable, respectively. In Models 5 and 7, air pollutants and their changing tendencies were added into the model based on Models 4 and 6, respectively. Some cells with sample sizes below 20 are presented, but not interpreted due to statistical power.

We estimated the unweighted population attributable fraction (PAF) of all-cause mortality with environmental, socioeconomic, and geographical risk factors, which indicates the fraction of mortality that could be prevented by eliminating certain risk factors from a population. We used the most common formula introduced by Levin in 1953 to calculate PAF:

$$\text{PAF}=\frac{P\left(\text{RR}\;-\;1\right)}{P\left(\text{RR}\;-\;1\right)\;+\;1}$$

in which P is the population prevalence of a risk factor and RR being the relative risk of that risk factor^36^

We used RStudio (R version 4.2.1) for all statistical analyses.

### Role of the funding sources

The funding sources have no role in the analytical research or writing process of this study.

## Results

### Ecological trends

**Fig. 1:**
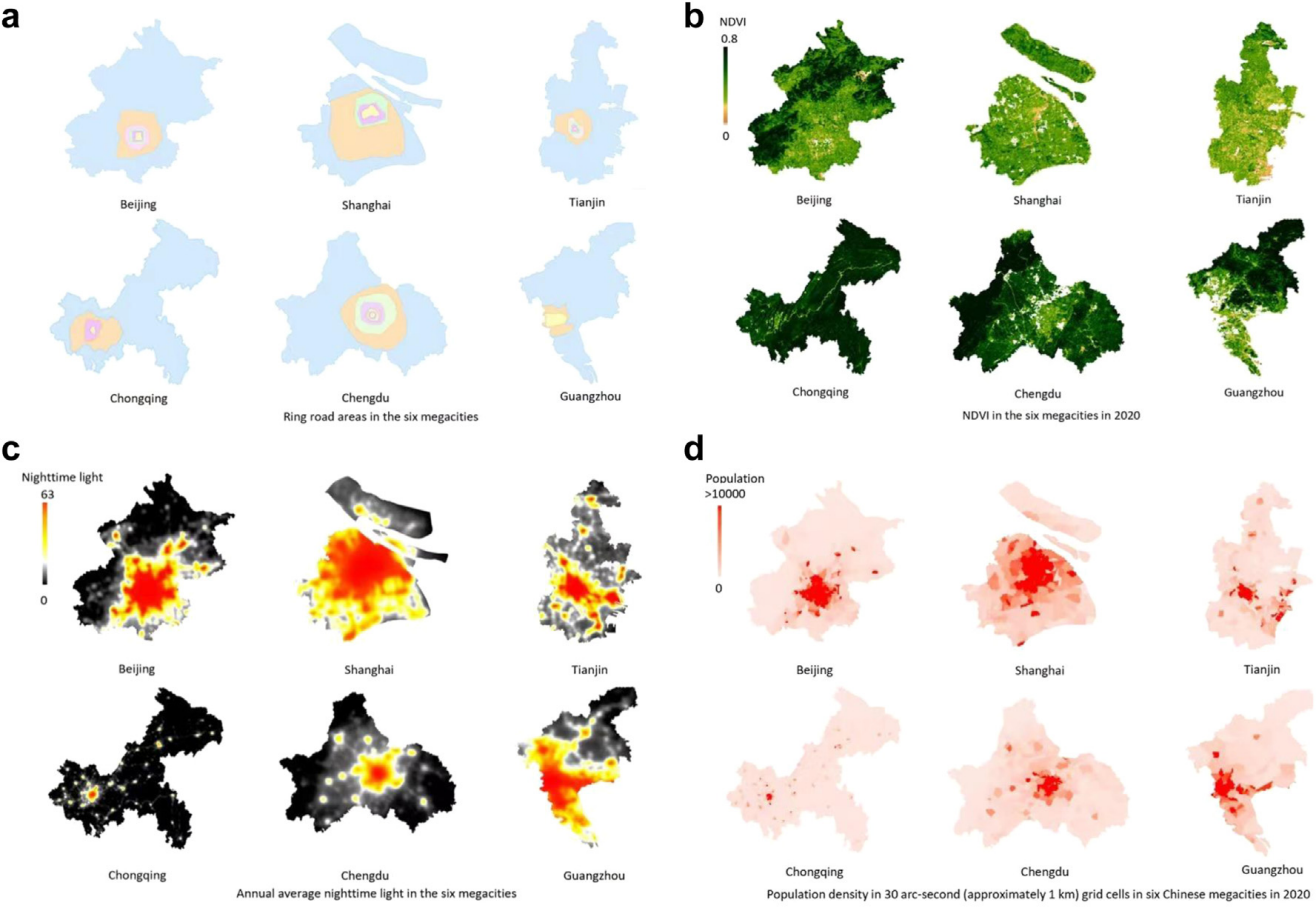
**Ring road areas, NDVI, nighttime light and population density in the six megacities.** (a) The figure shows ring road areas divided by ring roads in the six megacities. (b) The figure depicts the spatial distribution of NDVI in the six megacities in 2020. It indicates city centers had less vegetation while outer ring road areas were greener. NDVI data was obtained from the Moderate Resolution Imaging Spectroradiometer (MODIS) in the National Aeronautics and Space Administration (NASA)'s Terra satellite, which is updated every 16 days with a spatial resolution of 250 m. (c) The figure depicts the spatial distribution of average nighttime light during the period 2000–2011 in the six megacities, since we used CLHLS cohort data from 2000 to 2011. Nighttime light was significantly associated with proximity to city centers. In addition, we saw evidences of polycentricism in all the cities by using nighttime light as an indicator of economic activities. However, Beijing and Shanghai had closer economic subcenters to the main center in terms of distance, while Chongqing and Chengdu had the highest number of subcenters that were diffusely scattered around the city. The polycentric level in Tianjin and Guangzhou fell in between. Nighttime light data was obtained from Version 4 of the US Air Force Defense Meteorological Program (DMSP) Operational Line-Scan System (OLS) dataset that included nighttime light for calendar years from 1992 to 2013 with a resolution of 30-arc second (approximately 1 km). (d) The figure describes the spatial distribution of population density in the six megacities in 2020. Population density was also higher in city centers and its distribution pattern was found to be in accordance with economic centers in our analysis. We obtained Population data from The Gridded Population of World Version 4 (GPWv4), a minimally modeled global population dataset that uniformly distributes census data into 30 arc-second (approximately 1 km) grid cells, for the years 2000, 2005, 2010, 2015, and 2020.

**Fig. 2:**
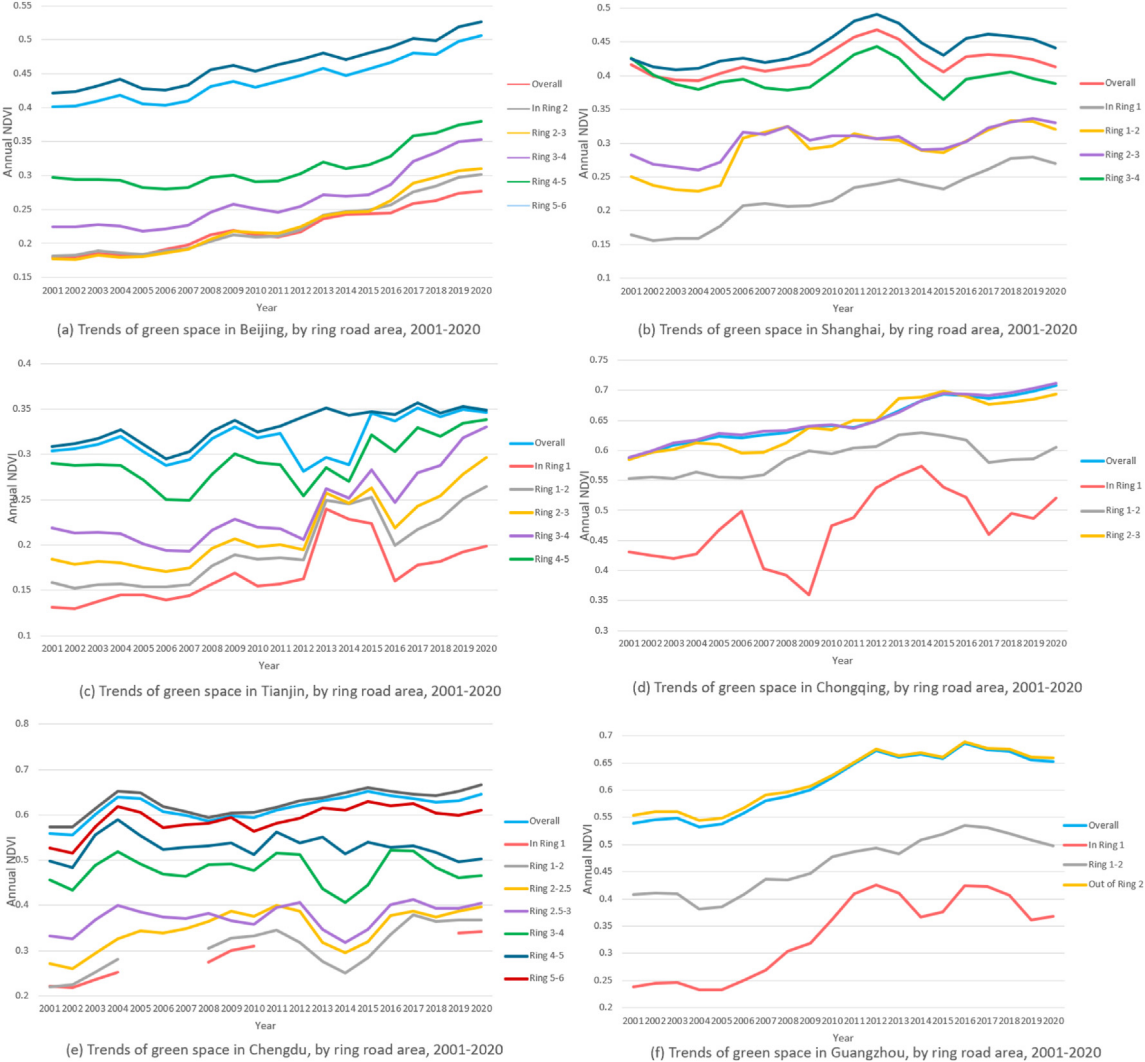

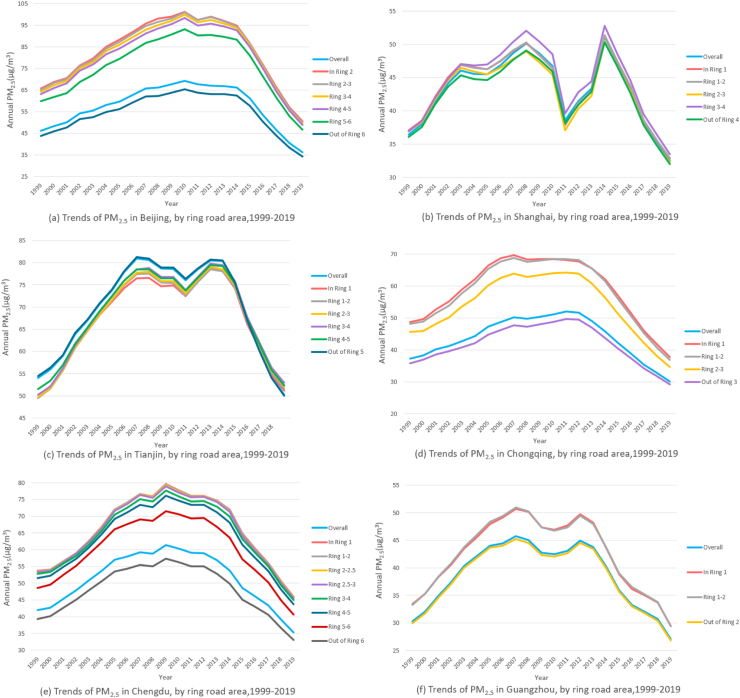

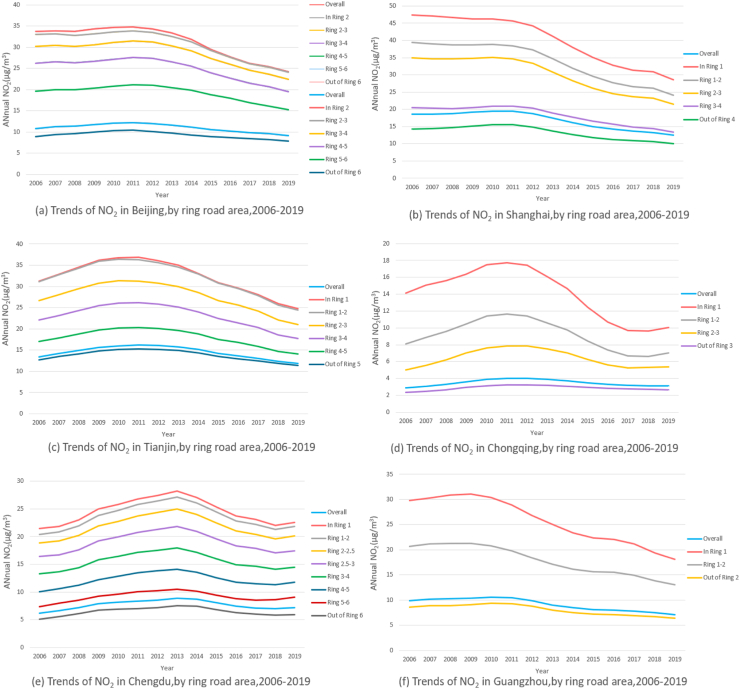

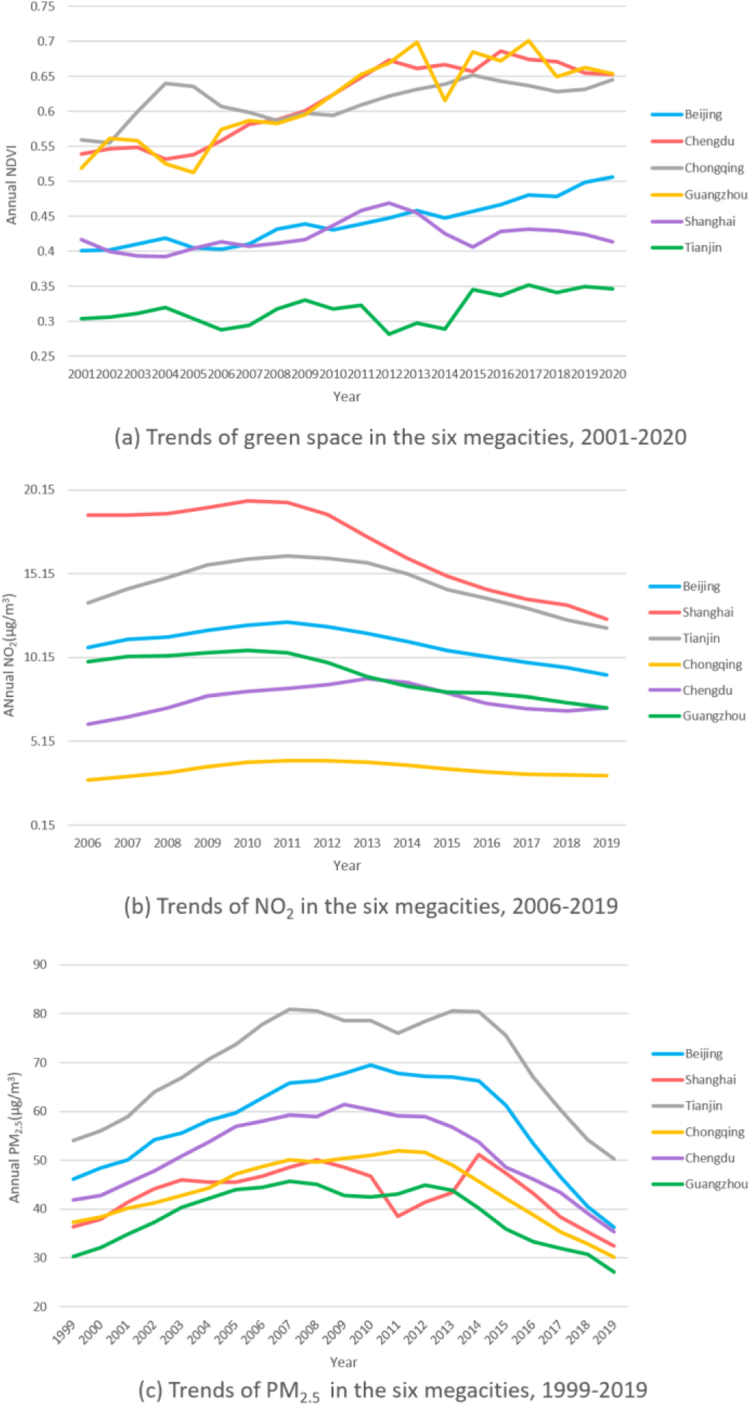
**1. Three-year moving averages of Normalized Difference Vegetation Index (NDVI), in the six megacities, by ring road area, 2001–2020.** Counts presented are the three-year moving average NDVI value calculated as the average of the NDVI observed in the given incident year, the preceding year and the following year. Our analysis of the six megacities revealed an overall upward trend in greenness levels, and that inner areas of the city had lower NDVI than outer ones. **2. Three-year moving averages of PM**_**2.5**_**concentration, in the six megacities, by ring road area, 1999–2019.** Counts presented are the three-year moving average PM_2.5_ value calculated as the average of the PM_2.5_ observed in the given incident year, the preceding year and the following year. The three-year moving average concentration of PM_2.5_ in the six megacities from 1999 to 2019 first increased with fluctuation and then declined steadily, following an inverse U-shape verified by regression models. Furthermore, between the steep upward and downward trend, PM_2.5_ concentration presented a slow decline and a closely followed rise from 2010 to 2013, making the overall evolution trend presenting an “M” shape, which was most evident in Shanghai. For residents in Beijing, Shanghai, Chongqing, Chengdu and Guangzhou, living in closer proximity to city centers was associated with higher PM_2.5_ exposure. However, we did not find similar trend from city center to the outskirts in Tianjin. We acquired ground-level PM_2.5_ for 1998–2020 from the Atmospheric Composition Analysis Group, which combined Aerosol Optical Depth (AOD) retrievals from the NASA MODIS, Multiangle Imaging Spectro Radiometer (MISR), and Sea-viewing Wide Field-of-view instruments with the GEOS-Chem chemical transport model, and subsequently calibrating to global ground-based observations using a Geographically Weighted Regression (GWR). **3. Three-year moving averages of NO**_**2**_**concentration, in the six megacities, by ring road area, 2006–2019.** Counts presented are the three-year moving average NO_2_ value calculated as the average of the NO_2_ observed in the given incident year, the preceding year and the following year. The relative levels of NO_2_ showed similar inverse U pattern in the city of Chengdu, Chongqing and Tianjin, while in Beijing, Shanghai and Guangzhou, NO_2_ showed a decreasing trend. NO_2_ was found to be higher in the city center in all the six megacities. The NO_2_ data in 2000 and 2005–2020 was obtained from a global NO_2_ land use regression (LUR) model created by Larkin and colleagues, with surface annual average NO_2_ concentrations at 0.0083° (∼1 km^2^) resolution. **4. Three-year moving averages of NDVI, NO**_**2**_**and PM**_**2.5**_**in the six megacities.** The three figures compare the level of NDVI and air pollution in the six megacities. Among the six megacities, Tianjin had the lowest greenness level and the highest PM_2.5_ exposure, whereas Guangzhou had the lowest PM_2.5_ and high NDVI. Shanghai had the highest NO_2_ exposure among the six megacities and Chongqing had the lowest.

**Fig. 3:**
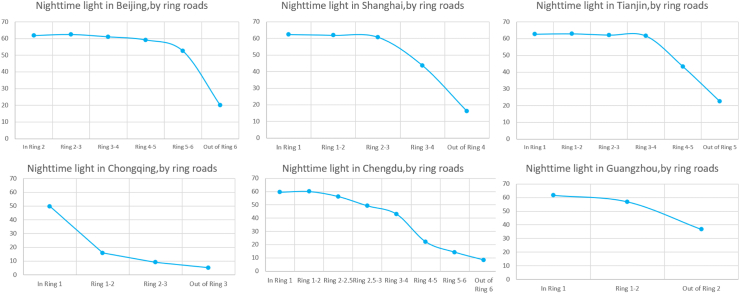
**Annual average nighttime light in six Chinese megacities, by ring roads. The above line charts illustrate nighttime light in the six megacities.** Data was downloaded from annual data set on night light in China Resource and Environment Science and Data Center using 0.01° grids (about 1 km). Nighttime light showed a decreasing trend from inner ring road areas to outer ring road areas in all the six megacities. Beijing, Shanghai and Tianjin had similar trend lines, with inner city residents exposed to a similar amount of nighttime light and those living towards outer ring roads. The other three cities had irregular trend lines, which might be attributable to their higher polycentric tendency.

**Fig. 4:**
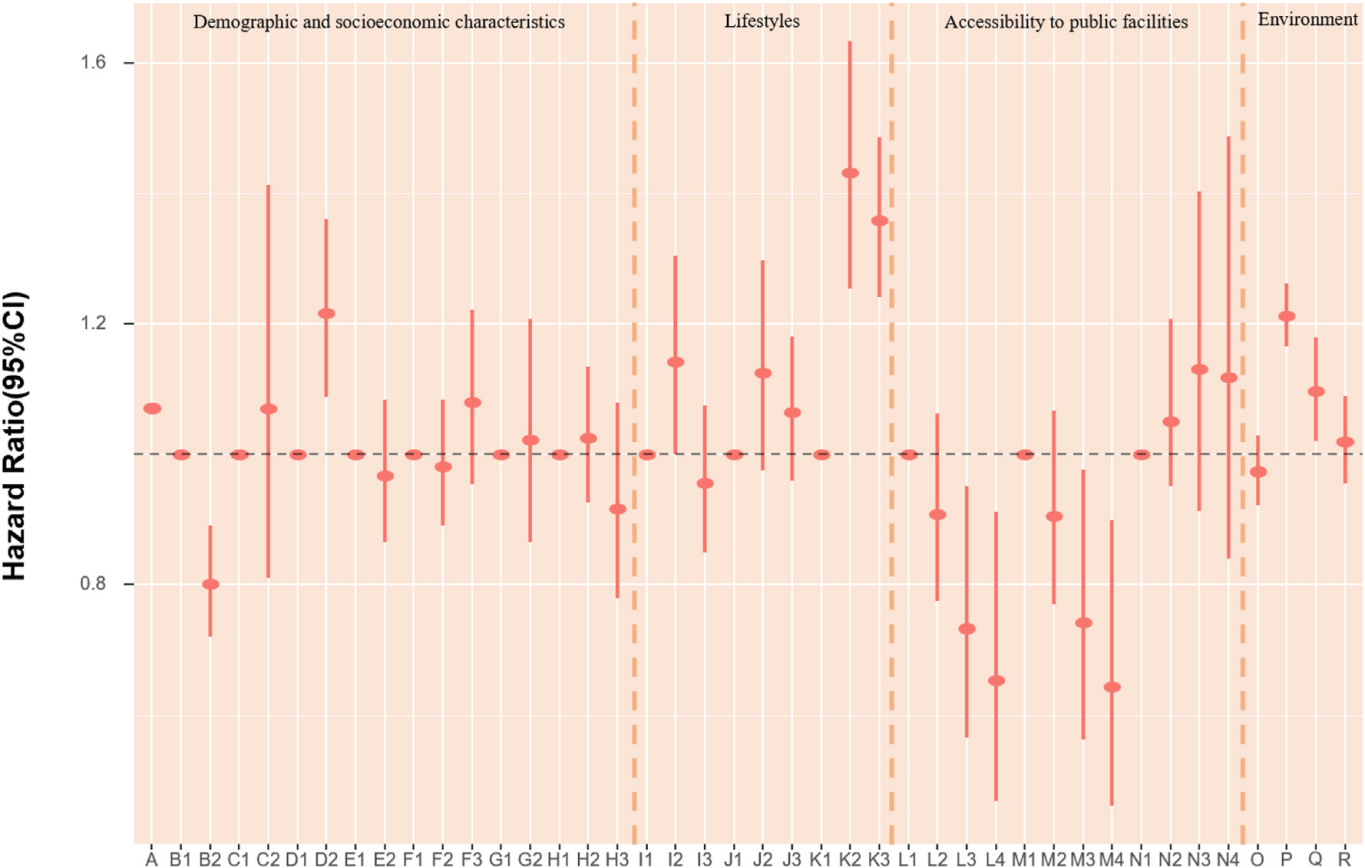

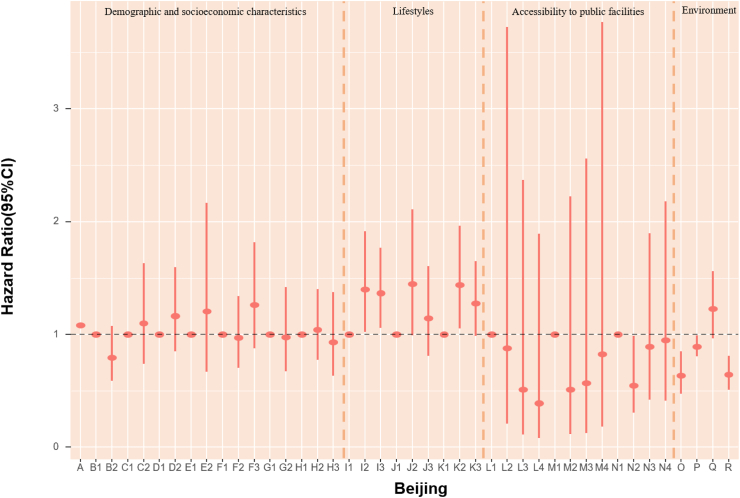

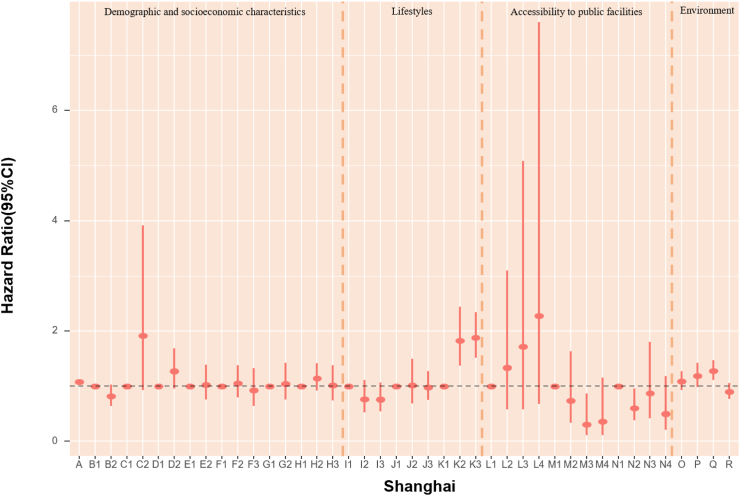

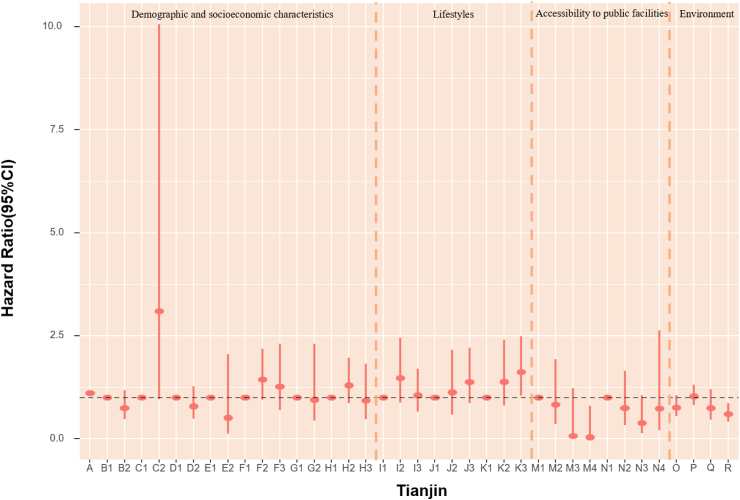

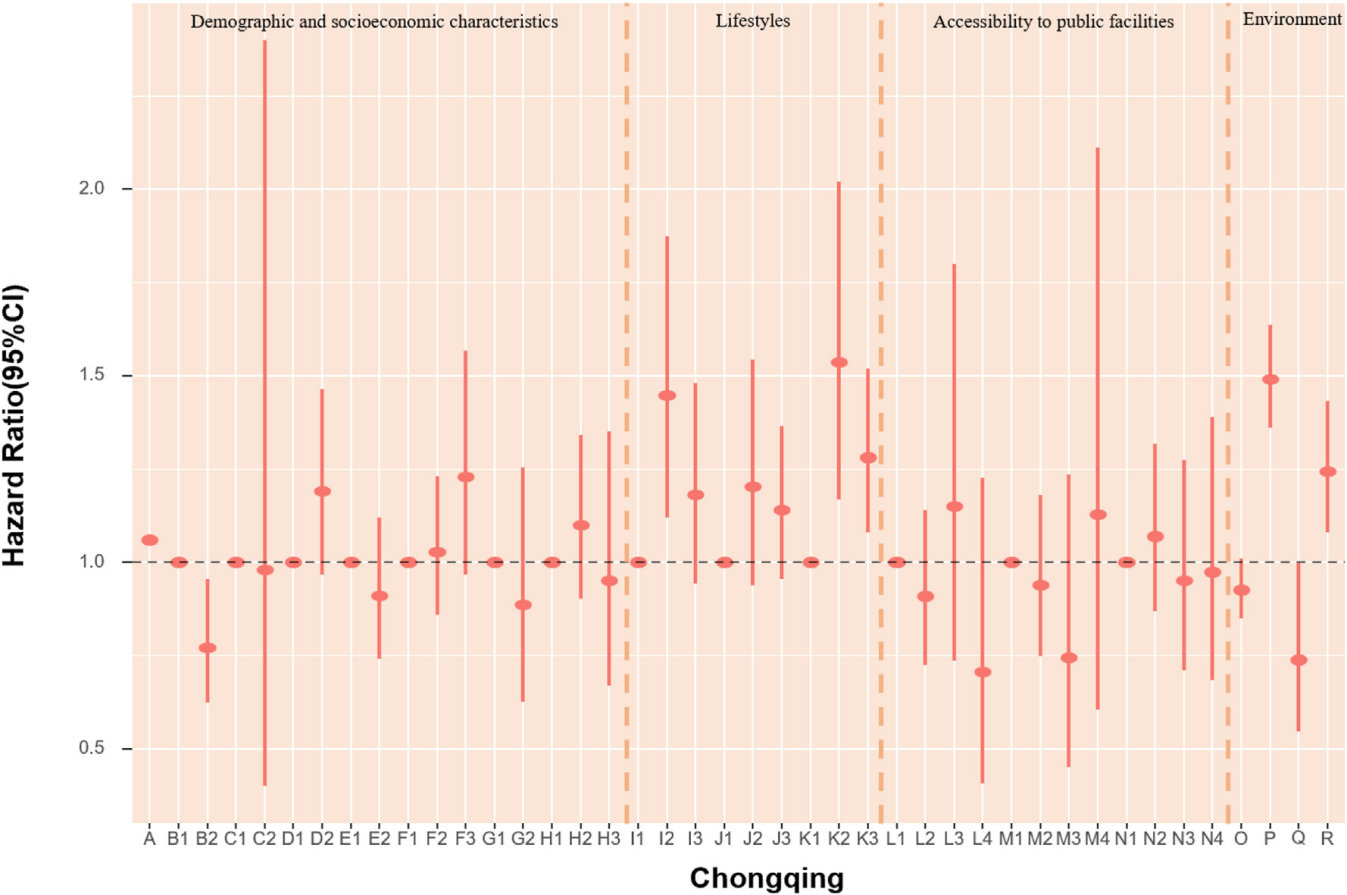

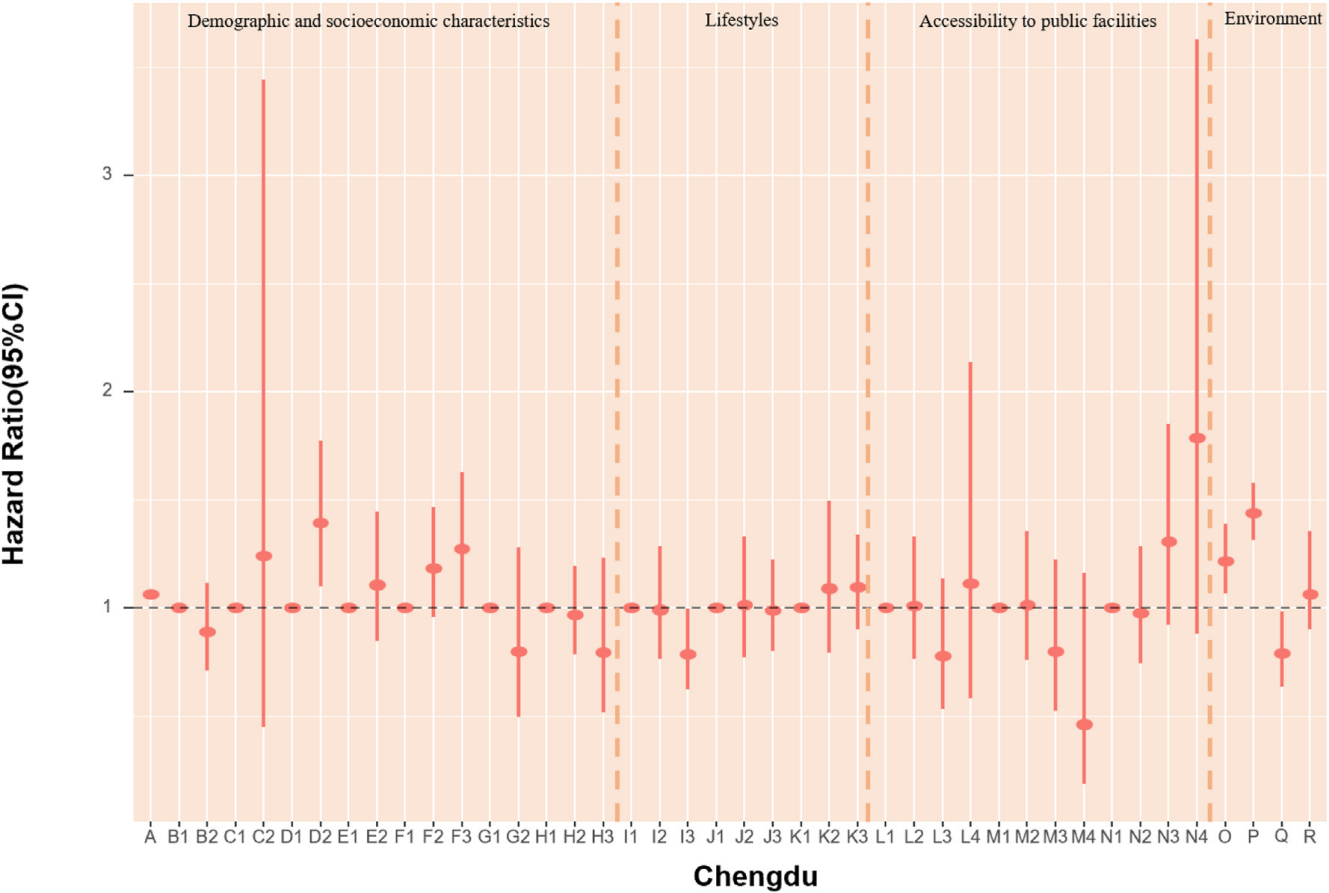

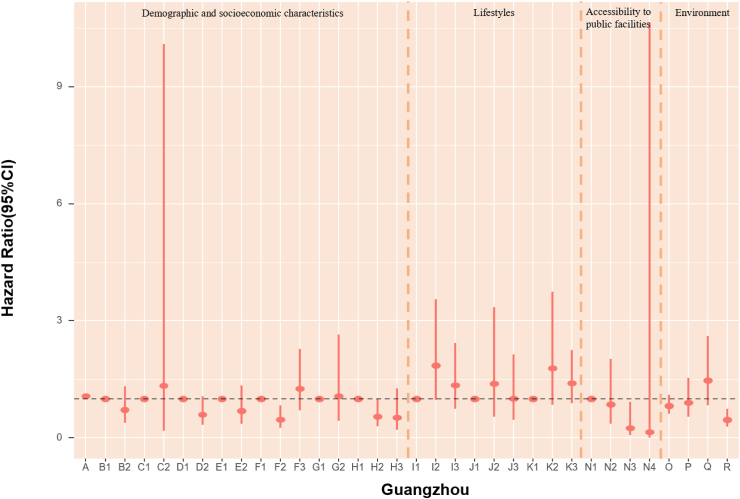
**1. HRs and 95% CIs for the association between all-cause mortality and demographic, socioeconomic, lifestyle, cumulative annual greenness, air pollution and nearby facilities (by quartiles of counts in 5 km-radius buffer) in the Cox model (N** = **3629).** This Cox regression model was also adjusted for cities, which were not presented in the figure. Nodes represent the hazard ratios; the line segments represent the 95%CIs and the solid single nodes are the references. Corresponding numeric data can be found in Table 5. A: Age. B: Sex, B1: Male, B2: Female. C: Ethnic, C1: Han, C2: Other ethnicities. D: Marital status, D1: Married and living with spouse, D2: Not co-habiting with a spouse. E: Residence, E1: residing in inner city, E2: residing in suburb areas. F: Household income, F1: Household income<¥5000, F2: Household income ¥5000–15000, F3: Household income>¥15,000. G: Occupation, G1: Non-manual occupation, G2: Other occupation. H: Education, H1: 0-year education, H2:1–6 years education, H3:>6 years education. I: Smoking, I1: Current, I2: Former, I3: Never. J: Alcohol, J1: Current, J2: Former, J3: Never; K: Exercise, K1: Current, K2: Former, K3: Never. L: Count of Medicine-related facilities in 5 km-radius buffer (per quartile increase). M: Count of Sports and leisure service-related places in 5 km-radius buffer (per quartile increase). N: Count of Scenic spots-related places in 5 km-radius buffer (per quartile increase). O: Cumulative annual NDVI (per 0.1-unit increase). P:PM_2.5_ (10 μg/m^3^). Q:NO_2_ (10 μg/m^3^). R: O_3_ (10 μg/m^3^). **2. HRs and 95% CIs for the association between all-cause mortality and demographic, socioeconomic, lifestyle, cumulative annual greenness, air pollution and nearby facilities (by quartiles of counts in 5 km-radius buffer) in different cities in the Cox model.** The above six Cox models were all adjusted for sex, age, ethnicity, marital status, occupation, education level, whether residing in inner-city or suburb, smoking status, alcohol status, exercise status, counts of public facilities (by quartile), NDVI and air pollutants. Nodes represent the hazard ratios; the line segments represent the 95%CIs and the solid single nodes are the references. Some variables were not presented in the figure due to their high HR, including counts of medicine-related facilities in Tianjin and medicine and leisure facilities in Guangzhou. Corresponding numeric data can be found in Table 5.

Our findings challenge the common perception that urban areas lack green spaces. Contrary, our analysis of the six megacities revealed an overall upward trend in greenness levels, with the largest increase observed in Chongqing, where the three-year moving averages of NDVI rose from 0.59 in 2001 to 0.71 in 2020. However, inner areas of the city had lower NDVI than outer ones. For instance, in Beijing, the mean cumulative NDVI from 2001 to 2020 was 0.22 within the second ring road. The number constantly went up to 0.31 between the fifth ring road and the sixth ring road before further rising to 0.46 beyond this area (Fig. 1a–b, Fig. 2-1, Table 1, Supplementary Figure S1). Among the six megacities, the measured greenness level by NDVI in Tianjin was the lowest compared with Chengdu, Guangzhou, and Chongqing (Fig. 2-4).

**Table 1:** Area-level green space and air pollutant in the six megacities.

	NDVI		PM_2.5_ (μg/m^3^)		NO_2_ (μg/m^3^)	
	Mean (SD)	Change	Mean (SD)	Change	Mean (SD)	Change
**Beijing**						
**Total**	0.44 (0.04)	Increase	56.0 (11.7)	Inverse U-shape	10.7 (1.2)	No change
**Ring road**						
In Ring 2	0.22 (0.04)	Increase	80.8 (17.8)	Inverse U-shape	30.6 (4.3)	Decrease
Ring 2–3	0.23 (0.04)	Increase	80.2 (17.8)	Inverse U-shape	30.1 (3.9)	Decrease
Ring 3–4	0.23 (0.05)	Increase	79.2 (17.6)	Inverse U-shape	27.9 (3.5)	Decrease
Ring 4–5	0.27 (0.05)	Increase	78.0 (17.2)	Inverse U-shape	24.4 (3.1)	Decrease
Ring 5–6	0.32 (0.03)	Increase	73.9 (16.3)	Inverse U-shape	18.7 (2.2)	Decrease
Out of Ring 6	0.47 (0.03)	Increase	52.9 (56.0)	Inverse U-shape	9.0 (1.0)	No change
**Shanghai**						
**Total**	0.42 (0.03)	No change	42.4 (7.4)	Inverse U-shape	16.5 (2.8)	Decrease
**Ring road**						
In Ring 1	0.22 (0.05)	Increase	42.7 (7.5)	Inverse U-shape	39.4 (7.7)	Decrease
Ring 1–2	0.29 (0.05)	Increase	42.7 (7.5)	Inverse U-shape	33.1 (6.3)	Decrease
Ring 2–3	0.30 (0.03)	Increase	42.0 (7.3)	Inverse U-shape	29.5 (5.7)	Decrease
Ring 3–4	0.40 (0.04)	No change	43.6 (7.9)	Inverse U-shape	17.9 (3.0)	Decrease
Out of Ring 4	0.44 (0.03)	Increase	41.8 (7.1)	Inverse U-shape	13.0 (2.1)	Decrease
**Tianjin**						
**Total**	0.33 (0.03)	Increase	68.0 (12.1)	Inverse U-shape	14.1 (1.7)	Inverse U-shape
**Ring road**						
In Ring 1	0.16 (0.02)	Increase	65.6 (11.5)	Inverse U-shape	31.4 (4.4)	Inverse U-shape
Ring 1–2	0.19 (0.03)	Increase	65.9 (11.7)	Inverse U-shape	31.1 (4.4)	Inverse U-shape
Ring 2–3	0.21 (0.04)	Increase	66.2 (11.7)	Inverse U-shape	26.8 (3.8)	Inverse U-shape
Ring 3–4	0.24 (0.04)	Increase	66.9 (11.8)	Inverse U-shape	22.4 (3.1)	Inverse U-shape
Ring 4–5	0.30 (0.03)	Increase	66.9 (11.5)	Inverse U-shape	17.5 (2.3)	Inverse U-shape
Out of Ring 5	0.33 (0.03)	Increase	68.2 (12.2)	Inverse U-shape	13.3 (1.6)	Inverse U-shape
**Chongqing**						
**Total**	0.65 (0.04)	Increase	42.8 (7.4)	Inverse U-shape	3.4 (0.4)	Inverse U-shape
**Ring road**						
In Ring 1	0.49 (0.06)	Increase	57.3 (11.2)	Inverse U-shape	13.8 (3.0)	Inverse U-shape
Ring 1–2	0.59 (0.04)	Increase	56.6 (11.3)	Inverse U-shape	9.0 (1.8)	Inverse U-shape
Ring 2–3	0.64 (0.05)	Increase	52.7 (10.4)	Inverse U-shape	6.2 (1.1)	Inverse U-shape
Out of Ring 3	0.65 (0.04)	Increase	41.0 (6.9)	Inverse U-shape	2.8 (0.3)	Inverse U-shape
**Chengdu**						
**Total**	0.61 (0.04)	Increase	50.1 (9.1)	Inverse U-shape	7.4 (1.0)	Inverse U-shape
**Ring road**						
In Ring 1	0.27 (0.06)	Increase	64.4 (11.9)	Inverse U-shape	24.0 (2.8)	Inverse U-shape
Ring 1–2	0.30 (0.06)	Increase	64.4 (11.9)	Inverse U-shape	23.0 (2.7)	Inverse U-shape
Ring 2–2.5	0.34 (0.07)	Increase	64.2 (11.9)	Inverse U-shape	21.2 (2.5)	Inverse U-shape
Ring 2.5–3	0.37 (0.06)	No change	64.0 (11.9)	Inverse U-shape	18.5 (2.2)	Inverse U-shape
Ring 3–4	0.48 (0.07)	No change	63.1 (11.6)	Inverse U-shape	15.2 (1.8)	Inverse U-shape
Ring 4–5	0.53 (0.06)	No change	61.8 (11.5)	Inverse U-shape	11.9 (1.5)	Inverse U-shape
Ring 5–6	0.59 (0.05)	No change	58.4 (11.0)	Inverse U-shape	8.9 (1.1)	Inverse U-shape
Out of Ring 6	0.63 (0.04)	Increase	46.9 (8.5)	Inverse U-shape	6.2 (0.9)	Inverse U-shape
**Guangzhou**						
**Total**	0.61 (0.06)	Increase	37.7 (7.0)	Inverse U-shape	9.0 (1.3)	Decrease
**Ring road**						
In Ring 1	0.33 (0.08)	Increase	41.3 (7.9)	Inverse U-shape	25.2 (4.9)	Decrease
Ring 1–2	0.46 (0.06)	Increase	41.4 (7.9)	Inverse U-shape	17.5 (3.2)	Decrease
Out of Ring 2	0.62 (0.06)	Increase	37.3 (7.0)	Inverse U-shape	7.9 (1.1)	Decrease

Data is the mean (SD) and change of area-level NDVI, PM_2.5_ and NO_2_ in the six megacities among ring road areas. Mean NDVI were calculated as the average values of annual NDVI from 2000 to 2021. Mean PM_2.5_ were calculated as the average values of ground-level PM_2.5_ for 1998–2020. Mean NO_2_ were calculated as the average values of annual NO_2_ in 2000 and 2005–2020. Change of the environmental measurements was estimated by putting every year's annual-average NDVI, PM_2.5_ and NO_2_ into a linear regression model and was defined as a significant increase or decrease if the regression coefficient was positive or negative, with its P-value less than 0.05. On the contrary, if the P-value was larger than 0.05, change was defined as non-significant. If the changing pattern was non-significant, we also evaluated whether NDVI, PM_2.5_ and NO_2_ showed an inverse U-shaped association with time by putting annual average values into regression models including linear and quadratic terms. The change was defined as following an inverse U shape if the regression model presents a significant positive linear (P value < 0.05 and coefficient >0) and negative quadratic (P value < 0.05 and coefficient <0) effect.

The three-year moving average concentration of PM_2.5_ in the six megacities from 1999 to 2019 first increased with fluctuation and then declined steadily, following an inverse U-shape verified by regression models. Furthermore, between the steep upward and downward trend, PM_2.5_ concentration presented a slow decline and a closely followed rise from 2010 to 2013, making the overall evolution trend presenting an “M” shape, which was most evident in Shanghai. For residents in Beijing, Shanghai, Chongqing, Chengdu, and Guangzhou, living in closer proximity to city centers was associated with higher PM_2.5_ exposure. However, we did not find a similar trend from the city center to the outskirts of Tianjin. PM_2.5_ exposure level in Tianjin showed an upward trend from 65.6 μg/m^3^ within the first ring road to 68.2 μg/m^3^ out of the fifth ring road (Fig. 2-2, Table 1, Supplementary Figure S2). Among the six megacities, PM_2.5_ exposure in Tianjin was the highest, whereas Guangzhou had the lowest PM_2.5_ (Fig. 2-4).

The relative levels of NO_2_ showed similar inverse U-pattern in the city of Chengdu, Chongqing and Tianjin, while in Beijing, Shanghai and Guangzhou, NO_2_ showed a decreasing trend. Highest NO_2_ level was found in the city center in all the six megacities (Fig. 2, Fig. 3, Table 1, Supplementary Figure S3). Shanghai had the highest NO_2_ exposure among the six megacities while Chongqing had the lowest (Fig. 2-4, Supplementary Figure S4).

Using nighttime light as an indicator, we saw evidence of polycentricism in all the cities. However, Beijing and Shanghai had closer economic subcenters to the main center in terms of distance, while Chongqing and Chengdu had the highest number of subcenters that were diffusely scattered around the city. The polycentric level in Tianjin and Guangzhou fell in between. Population distribution was found to be in accordance with economic centers in our analysis (Fig. 1c–d).

### Epidemiologic cohort participant characteristics

We followed 4992 participants whose mean age was 87.8 years (SD:11.7) at baseline, 3775 (75.6%) of whom were aged 80 years and older. There were slightly more female participants (2887; 57.8%), and a majority of *Han* Chinese (97.9%). Most of the participants were not co-habiting with a spouse (72.0%), had previously worked in manual labor (88.5%), without formal education (53.4%), never smoked (64.6%) or consumed alcohol (69.1%) (Table 2). We found that some subgroups lived with higher air pollution, lower greenness, and easier accessibility to public facilities, such as ethnic minorities, participants who lived in inner-city, who had higher household income, who had previously worked in non-manual labor, who received formal education less than one year and who were former smokers (Supplementary Table S1).

**Table 2:** Characteristics of participants.

Characteristics	Total	Beijing	Shanghai	Tianjin	Chongqing	Chengdu	Guangzhou
Age, years	87.81 (11.68)	88.7 (11.10)	88.51 (12.00)	86.07 (11.51)	88.25 (11.45)	89.04 (10.78)	80.83 (12.32)
Age group							
65–79 years	24.4%	21.3%	26.0%	27.9%	21.6%	16.8%	49.4%
80–89 years	23.1%	21.5%	15.7%	24.3%	26.8%	30.8%	21.2%
90–99 years	28.2%	34.7%	28.6%	29.6%	26.6%	27.6%	18.5%
≥100 years	24.2%	22.5%	29.8%	18.2%	25.1%	24.8%	11.0%
Sex							
Male	42.2%	41.6%	42.7%	41.62%	38.5%	45.9%	44.7%
Female	57.8%	58.4%	57.4%	58.4%	61.5%	54.1%	55.3%
Ethnicity							
Han Chinese	97.9%	91.7%	99.2%	98.0%	99.4%	99.1%	98.4%
Ethnic minorities	2.1%	8.3%	0.8%	2.0%	0.4%	0.8%	1.6%
Marital status							
Married and living with spouse	28.0%	26.8%	30.6%	31.3%	24.8%	24.7%	38.1%
Other	72.0%	73.6%	69.4%	68.7%	75.3%	75.3%	61.9%
Residence							
Inner-city	65.9%	76.9%	64.0%	53.6%	44.0%	87.6%	76.0%
Suburb	34.1%	23.1%	36.0%	46.4%	56.0%	12.4%	24.0%
Household income (RMB)							
<5000	36.77%	18.0%	12.6%	36.6%	53.4%	46.0%	33.7%
5000–15000	36.32%	34.5%	53.4%	40.55%	25.9%	25.8%	25.3%
>15,000	18.07%	25.2%	19.8%	14.8%	11.4%	18.8%	19.1%
Main occupation before 60 years of age							
Non-manual	11.3%	16.9%	15.4%	13.1%	6.1%	6.1%	12.5%
Other	88.5%	82.9%	84.5%	86.9%	93.8%	93.6%	87.2%
Education, years	2.78 (8.27)	3.17 (8.34)	4.03 (9.57)	2.59 (5.95)	1.91 (8.14)	2.04 (8.25)	2.29 (3.92)
Education							
0 year	53.4%	52.3%	41.7%	54.8%	65.4%	58.4%	45.7%
1–6 year	31.1%	28.4%	34.7%	28.8%	26.3%	32.5%	37.9%
>6 years	14.7%	18.6%	22.6%	16.2%	7.6%	8.1%	16.2%
Smoking status							
Current	19.1%	16.3%	11.0%	21.5%	19.9%	30.0%	21.2%
Former	16.2%	18.5%	14.2%	21.8%	13.2%	19.5%	15.1%
Never	64.6%	65.2%	74.6%	56.7%	67.0%	50.4%	63.7%
Alcohol status							
Current	19.1%	16.9%	15.5%	13.4%	24.2%	24.3%	13.3%
Former	11.7%	12.0%	8.4%	12.0%	12.5%	16.7%	7.8%
Never	69.1%	71.0%	76.2%	74.6%	63.3%	58.8%	78.9%
Exercise							
Current	37.7%	45.0%	33.4%	38.3%	32.3%	37.0%	50.1%
Former	11.2%	17.0%	13.1%	12.3%	8.0%	9.6%	6.0%
Never	51.3%	37.7%	53.5%	49.4%	59.7%	53.1%	43.9%

Data is percentages or mean (SD).

### Environmental characteristic of city centers

Proximity to the city center was associated with lower green space availability, higher air pollution levels, and better access to public facilities. For example, areas within the second ring road in Beijing had the least amount of green space (NDVI = 0.19, SD = 0.03), whereas areas outside the sixth ring road had much higher NDVI values of 0.35 (SD = 0.06). Additionally, inner-city locations had a higher density of public facilities (Table 3). For air pollutants, the annual average PM_2.5_ and NO_2_ had a spread of 81.6 μg/m^3^ and 24.7 μg/m^3^ within the second ring road to 70.7 μg/m^3^ and 12.5 μg/m^3^ out of the sixth ring road. It is intriguing to note that although PM_2.5_ and NO_2_ exhibited a similar trend from the city center to outskirts in Shanghai, Chongqing, Chengdu and Guangzhou as Beijing, this pattern was not evident in Tianjin, where PM_2.5_ levels showed an upward trend from 64.6 μg/m^3^ within the first ring road to 71.5 μg/m^3^ out of the fifth ring road. On the contrary to PM_2.5_ and NO_2,_ ozone exposure was higher in Beijing, Shanghai, and Chongqing's outlying areas, whereas it was not substantially correlated with location in the other three megacities (Table 4). Nighttime light showed a decreasing trend from inner ring road areas to outer ring road areas in all the six megacities. Beijing, Shanghai and Tianjin had similar trend lines, with participants living in inner areas seeing a similar amount of nighttime light and those living in the outermost two ring road areas experiencing a considerable reduction in nighttime light. The other three cities had irregular trend lines, which might be attributable to their higher polycentric tendency (Fig. 3).

**Table 3:** Nearby facilities and green space in megacities.

	Nearby facilities							Green space					
	Distance to the nearest facility			Count of facilities in 5 km buffer area				NDVI			NDVI change		
	Medicine	Leisure	Landscape	Overall	Medicine	Leisure	Landscape	Mean (SD)	Min	Max	No change or missing	Increase	Decrease
**Beijing**													
**Total**	0.37 (0.62)	0.29 (0.49)	0.51 (0.65)	52,603	1426 (928)	1929 (1359)	1018 (929)	0.24 (0.08)	0.13	0.52	43.60%	56.40%	0
**Ring road**													
In Ring 2	0.15 (0.12)	0.12 (0.09)	0.16 (0.09)	93,476	2336 (161)	1861 (630)	2194 (353)	0.19 (0.03)	0.13	0.34	40.10%	59.90%	0
Ring 2–3	0.13 (0.07)	0.11 (0.08)	0.23 (0.13)	80,671	2127 (228)	2909 (819)	1199 (477)	0.20 (0.04)	0.15	0.29	37.80%	62.20%	0
Ring 3–4	0.16 (0.10)	0.14 (0.09)	0.27 (0.20)	58,397	1692 (271)	2562 (933)	632 (244)	0.22 (0.05)	0.14	0.32	41.70%	58.30%	0
Ring 4–5	0.28 (0.21)	0.21 (0.21)	0.39 (0.25)	31,992	913 (278)	1347 (588)	367 (233)	0.25 (0.03)	0.16	2.47	55.40%	44.60%	0
Ring 5–6	0.30 (0.24)	0.27 (0.22)	0.66 (0.32)	13,006	459 (300)	550 (340)	197 (182)	0.27 (0.05)	0.16	0.38	40.20%	59.80%	0
Out of Ring 6	1.17 (1.04)	0.86 (0.88)	1.44 (0.94)	2640	89 (137)	115 (130)	34 (35)	0.35 (0.06)	0.21	0.52	52.50%	47.50%	0
**Shanghai**													
**Total**	0.32 (0.46)	0.24 (0.28)	0.56 (0.88)	87,203	2096 (1309)	3570 (2292)	971 (728)	0.23 (0.13)	−0.17	0.55	60.20%	38.20%	1.60%
**Ring road**													
In Ring 1	0.14 (0.11)	0.09 (0.07)	0.20 (0.16)	161,825	3214 (488)	5365 (1024)	1585 (374)	0.15 (0.06)	−0.17	0.34	53.50%	46.50%	0
Ring 1–2	0.16 (0.17)	0.11 (0.07)	0.35 (0.21)	95,761	2066 (601)	3468 (1148)	691 (343)	0.18 (0.06)	0.05	0.4	49.80%	50.20%	0
Ring 2–3	0.29 (0.29)	0.19 (0.18)	0.40 (0.23)	50,698	1158 (478)	1941 (764)	299 (126)	0.22 (0.06)	0.09	0.36	55.30%	44.70%	0
Ring 3–4	0.49 (0.40)	0.35 (0.30)	0.75 (0.57)	7817	263 (160)	418 (298)	90 (63)	0.40 (0.08)	0.1	0.51	87.40%	2.30%	10.30%
Out of Ring 4	1.03 (0.72)	0.84 (0.66)	2.04 (1.45)	2209	47 (55)	86 (89)	40 (69)	0.44 (0.06)	0.22	0.55	86.00%	8.00%	6.00%
**Tianjin**													
**Total**	0.53 (0.81)	0.73 (1.32)	1.43 (1.88)	45,383	1169 (965)	1117 (1016)	331 (341)	0.24 (0.10)	0.12	0.47	48.00%	51.70%	0.30%
**Ring road**													
In Ring 1	0.13 (0.09)	0.10 (0.05)	0.13 (0.15)	110,830	2546 (56)	2667 (144)	892 (38)	0.14 (0.01)	0.12	0.16	44.40%	55.60%	0
Ring 1–2	0.13 (0.09)	0.16 (0.13)	0.38 (0.29)	91,575	2128 (277)	2170 (493)	698 (167)	0.17 (0.02)	0.14	0.2	37.20%	62.80%	0
Ring 2–3	0.13 (0.11)	0.15 (0.11)	0.44 (0.29)	56,095	1529 (301)	1297 (400)	321 (164)	0.18 (0.02)	0.15	0.22	47.10%	52.90%	0
Ring 3–4	0.26 (0.18)	0.27 (0.23)	0.69 (0.53)	23,376	758 (261)	607 (211)	73 (34)	0.21 (0.04)	0.15	0.27	58.30%	41.70%	0
Ring 4–5	0.61 (0.48)	0.59 (0.40)	1.87 (0.54)	7370	159 (160)	128 (93)	19 (18)	0.27 (0.03)	0.2	0.31	75.00%	18.80%	6.30%
Out of Ring 5	1.17 (1.06)	1.69 (1.84)	3.10 (2.24)	2300	124 (231)	96 (209)	20 (49)	0.35 (0.08)	0.15	0.47	51.90%	48.10%	0
**Chongqing**													
**Total**	1.10 (1.14)	1.41 (1.66)	1.41 (1.23)	12,509	521 (896)	435 (846)	95 (165)	0.53 (0.13)	0.07	0.73	63.10%	34.40%	2.50%
**Ring road**													
In Ring 1	0.21 (0.21)	0.23 (0.17)	0.54 (0.36)	60,985	2185 (928)	1994 (967)	378 (195)	0.35 (0.12)	0.07	0.67	71.70%	28.30%	0
Ring 1–2	0.70 (0.57)	0.74 (0.42)	1.11 (0.72)	6937	273 (309)	216 (273)	73 (102)	0.57 (0.09)	0.18	0.72	73.90%	15.90%	10.20%
Ring 2–3	1.26 (1.17)	1.26 (1.26)	1.35 (1.19)	4524	195 (252)	97 (119)	31 (39)	0.55 (0.11)	0.16	0.73	50.50%	38.10%	1.40%
Out of Ring 3	1.61 (1.27)	2.39 (2.05)	2.00 (1.40)	1737	85 (176)	39 (81)	15 (18)	0.56 (0.10)	0.15	0.73	55.40%	43.90%	0.70%
**Chengdu**													
**Total**	0.61 (0.56)	0.51 (0.53)	0.86 (0.68)	30,052	1318 (1768)	978 (1298)	235 (304)	0.51 (0.16)	0.17	0.81	75.00%	21.70%	3.30%
**Ring road**													
In Ring 1	0.13 (0.07)	0.12 (0.06)	0.21 (0.14)	142,133	5351 (440)	3935 (343)	960 (82)	0.25 (0.03)	0.18	0.28	38.80%	61.20%	0
Ring 1–2	0.09 (0.08)	0.09 (0.05)	0.27 (0.13)	114,893	3973 (507)	3065 (304)	700 (124)	0.27 (0.05)	0.19	0.36	37.80%	62.20%	0
Ring 2–2.5	0.16 (0.10)	0.17 (0.09)	0.37 (0.18)	85,563	3076 (741)	2299 (459)	504 (131)	0.30 (0.07)	0.17	0.46	47.40%	52.60%	0
Ring 2.5–3	0.40 (0.32)	0.37 (0.28)	0.50 (0.33)	40,178	1651 (740)	1089 (477)	224 (58)	0.32 (0.06)	0.22	0.4	79.30%	20.70%	0
Ring 3–4	0.40 (0.31)	0.42 (0.29)	0.65 (0.28)	34,492	1408 (658)	885 (381)	154 (51)	0.46 (0.08)	0.29	0.6	100%	0	0
Ring 4–5	0.60 (0.40)	0.51 (0.43)	1.23 (0.69)	9869	373 (259)	303 (200)	56 (40)	0.52 (0.07)	0.38	0.69	90.90%	1.30%	7.80%
Ring 5–6	0.80 (0.83)	0.44 (0.42)	0.70 (0.35)	7167	325 (250)	221 (144)	68 (39)	0.56 (0.05)	0.46	0.67	83.60%	6.60%	9.80%
Out of Ring 6	0.87 (0.55)	0.74 (0.61)	1.17 (0.70)	4332	173 (229)	152 (168)	60 (67)	0.62 (0.07)	0.3	0.81	84.30%	11.60%	4.10%
**Guangzhou**													
**Total**	0.44 (0.69)	0.40 (0.79)	0.52 (0.71)	53,079	1697 (1523)	1707 (159)	695 (671)	0.35 (0.14)	0.08	0.74	73.20%	24.50%	2.10%
**Ring road**													
In Ring 1	0.15 (0.12)	0.11 (0.09)	0.17 (0.14)	125,327	3320 (793)	3415 (740)	1381 (447)	0.24 (0.06)	0.08	0.49	57.90%	42.10%	0
Ring 1–2	0.35 (0.23)	00.26 (0.17)	0.41 (0.17)	40,053	1219 (672)	1211 (484)	518 (232)	0.33 (0.14)	0.13	0.7	88.70%	11.30%	0
Out of Ring 2	0.74 (0.93)	0.72 (1.10)	0.87 (0.93)	10,238	335 (298)	272 (266)	111 (131)	0.44 (0.10)	0.22	0.74	83.00%	12.30%	4.70%

Cumulative annual NDVI was the mean of the annual average NDVI during follow-up period from the baseline year to the death year for deceased individuals and to the last interview year for those still alive at follow-up and those lost to follow-up. Data presented in the table are the mean (SD), minimum, and maximum values, as well as the change of public facilities and NDVI in the six megacities among ring road areas. Change of NDVI was estimated by putting every year's annual average NDVI over each participant's follow-up period into a linear regression model and was defined as a significant increase or decrease if the regression coefficient was positive or negative, with its P-value less than 0.05. On the contrary, if the P-value was larger than 0.05, change was defined as non-significant.

**Table 4:** Air pollutants exposure in the six megacities.

	PM_2.5_				NO_2_				O_3_				
	Mean (Min, Max)	No change or missing	Increase	Decrease	Mean (Min, Max)	No change or missing	Increase	Decrease	Mean (Min, Max)	No change or missing	Increase	Decrease	
**Beijing**													
**Total**	81.61 (33.80, 107.30)	100%	0	0	24.69 (1.61, 41.89)	99.60%	0.40%	0	78.27 (62.03, 100.24)	85.60%	14.40%	0	
**Ring road**													
In Ring 2	87.12 (50.00, 106.40)	100%	0	0	31.15 (2.38, 41.89)	99.60%	0.40%	0	76.66 (65.72, 94.96)	87%	13.00%	0	
Ring 2–3	86.98 (53.40, 107.30)	100%	0	0	31.03 (10.17, 38.13)	100%	0	0	77.15 (64.69, 93.86)	86.50%	13.50%	0	
Ring 3–4	84.06 (45.60, 105.40)	100%	0	0	27.82 (2.46, 37.31)	100%	0	0	78.36 (62.03, 97.10)	87.50%	12.50%	0	
Ring 4–5	82.72 (47.90, 105.80)	100%	0	0	23.70 (7.63, 34.00)	100%	0	0	79.32 (68.10, 97.60)	83.10%	16.90%	0	
Ring 5–6	77.58 (42.20, 101.30)	100%	0	0	19.92 (1.61, 28.73)	100%	0	0	78.98 (65.76, 98.78)	84.30%	15.70%	0	
Out of Ring 6	70.65 (33.80, 99.70)	100%	0	0	12.48 (1.61, 23.00)	98.70%	1.30%	0	80.34 (66.43, 100.24)	82.90%	17.10%	0	
**Shanghai**													
**Total**	45.54 (22.30, 55.40)	100%	0 (0)	0 (0)	36.72 (0.00, 60.09)	98.30%	0	1.70%	70.69 (59.47, 100.99)	60.20%	39.80%	0	
**Ring road**													
In Ring 1	46.71 (24.50, 54.90)	100%	0 (0)	0 (0)	46.47 (3.01, 60.09)	99.90%	0	0.10%	68.85 (59.75, 95.89)	60.90%	39.10%	0	
Ring 1–2	46.51 (24.90, 54.20)	100%	0 (0)	0 (0)	42.83 (7.31, 56.73)	98.20%	0	1.80%	69.44 (59.47, 95.05)	57.20%	42.80%	0	
Ring 2–3	45.49 (24.30, 51.50)	100%	0 (0)	0 (0)	37.50 (12.04, 57.10)	97.70%	0	2.30%	70.29 (62.19, 81.48)	0	40.20%	0	
Ring 3–4	44.03 (33.40, 53.70)	100%	0 (0)	0 (0)	16.22 (9.08, 40.00)	98.90%	0	1.10%	73.90 (61.83, 92.12)	0	40.20%	0	
Out of Ring 4	42.07 (22.30, 55.30)	100%	0 (0)	0 (0)	14.40 (0.00, 31.93)	93.50%	0	6.50%	75.79 (59.68, 100.99)	61.50%	38.50%	0	
**Tianjin**													
**Total**	70.63 (46.80, 90.40)	100%	0	0	22.20 (2.27, 45.94)	100%	0	0	81.45 (68.73, 98.21)	75.70%	24.30%	0	
**Ring road**													
In Ring 1	64.58 (52.50, 77.40)	100%	0	0	28.62 (12.90, 37.56)	100%	0	0	81.82 (71.91, 96.73)	0	38.90%	0	
Ring 1–2	69.75 (52.90, 84.80)	100%	0	0	31.27 (24.97, 45.93)	100%	0	0	79.30 (69.22, 96.95)	0	32.10%	0	
Ring 2–3	72.71 (52.20, 85.40)	100%	0	0	27.06 (5.01, 34.15)	100%	0	0	80.19 (71.38, 97.09)	73.60%	26.40%	0	
Ring 3–-4	73.04 (55.3, 79.40)	100%	0	0	24.85 (17.29, 36.00)	100%	0	0	83.24 (75.14, 96.51)	91.70%	8.30%	0	
Ring 4–5	68.22 (54.60, 90.40)	100%	0	0	17.37 (13.96, 21.86)	100%	0	0	80.92 (73.11, 84.43)	87.50%	12.50%	0	
Out of Ring 5	71.54 (46.80, 90.40)	100%	0	0	12.80 (2.27, 24.00)	100%	0	0	83.30 (68.73, 98.21)	82.90%	7.10%	0	
**Chongqing**													
**Total**	59.85 (22.60, 777.90)	99.80%	0	2 (0.2%)	8.16 (0.82, 35.00)	85.10%	8.60%	6.30%	71.42 (37.12, 84.43)	65%	0.20%	34.80%	
**Ring road**													
In Ring 1	62.89 (38.30, 73.60)	100%	0	0	15.27 (2.86, 35.00)	77.30%	0.50%	22.20%	68.33 (37.12, 84.43)	51.90%	0.50%	47.60%	
Ring 1–2	61.32 (36.70, 76.10)	100%	0	0	8.85 (4.48, 15.14)	87.20%	0.40%	12.40%	70.13 (51.30, 82.33)	34.80%	0	64.20%	
Ring 2–3	59.97 (34.40, 77.90)	100%	0	0	7.61 (1.96, 15.26)	99.30%	0.70%	0	72.42 (53.74, 82.84)	70.10%	0	29.90%	
Out of Ring 3	57.88 (22.60, 77.10)	99.60%	0	2 (0.4%)	5.48 (0.82, 20.17)	78.70%	21.30%	0	72.65 (57.48, 85.53)	82.20%	0.20%	17.60%	
**Chengdu**													
**Total**	58.94 (32.40, 83.90)	100%	0	0	13.10 (1.77, 30.20)	74.90%	25.10%	0	75.72 (58.69, 88.79)	95.80%	33 (3.6%)	6 (0.6%)	
**Ring road**													
In Ring 1	70.19 (45.60, 83.90)	100%	0	0	21.55 (1.88, 30.20)	89.40%	10.60%	0	76.99 (61.81, 82.80)	100%	0	0	
Ring 1–2	70.76 (49.60, 83.40)	100%	0	0	22.51 (17.67, 30.11)	93.30%	6.70%	0	77.36 (63.21, 83.95)	100%	0	0	
Ring 2–2.5	69.63 (45.40, 83.70)	100%	0	0	19.74 (1.81, 28.04)	76.30%	23.70%	0	77.23 (63.84, 84.36)	93.80%	0	6.20%	
Ring 2.5–3	72.79 (45.10, 81.90)	100%	0	0	15.66 (7.05, 29.85)	79.30%	20.70%	0	78.13 (63.53, 85.01)	100%	0	0	
Ring 3–4	67.55 (44.80, 80.80)	100%	0	0	14.48 (9.84, 20.61)	91.70%	8.30%	0	80.74 (73.48, 85.66)	97.90%	2.10%	0	
Ring 4–5	66.96 (42.20, 83.30)	100%	0	0	10.42 (1.77, 17.61)	46.10%	53.90%	0	78.48 (62.51, 84.93)	100%	0	0	
Ring 5–6	64.51 (41.80, 81.50)	100%	0	0	10.79 (4.63, 19.09)	55.70%	44.30%	0	77.79 (64.19, 86.11)	100%	0	0	
Out of Ring 6	49.97 (32.40, 77.70)	100%	0	0	10.19 (2.15, 23.57)	75.10%	24.90%	0	73.65 (58.69, 88.79)	93.00%	7.00%	0	
**Guangzhou**													
**Total**	45.18 (30.30, 53.80)	100%	0	0	19.42 (1.99, 47.73)	100%	0	0	78.55 (67.04, 93.94)	57.70%		42.30%	0
**Ring road**													
In Ring 1	45.75 (33.40, 52.50)	100%	0	0	30.28 (2.01, 47.73)	100%	0	0	77.74 (67.04, 90.67)	71.70%		28.30%	0
Ring 1–2	45.90 (33.90, 53.40)	100%	0	0	19.71 (8.87, 30.33)	100%	0	0	79.98 (68, 75, 91.8)	56.60%		43.40%	0
Out of Ring 2	44.57 (30.30, 53.80)	100%	0	0	12.30 (1.99, 25.61)	100%	0	0	78.73 (67.39, 93.94)	45%		55.00%	0

Data presented in the table is the mean (SD), minimum, maximum values and change of last-year air pollutants in the six megacities among ring road areas. We considered the exposure window for PM2.5, NO2, and ozone as the annual average value in the death year for deceased individuals and in the last interview year for those still alive at follow-up and those lost to follow-up. As NO2 data from 2001 to 2004 were unavailable, the NO2 concentration in 2000 was used as the last-year NO2 for participants whose end-up year was 2001 or 2002, and NO2 concentration in 2005 was used as the last-year NO2 for those with the end-up year 2003 or 2004. Change of air pollutants was estimated by putting annual-average values of air pollutants over each participant's follow-up period into a linear regression model. It was defined as a significant increase or decrease if the regression coefficient was positive or negative, with its P-value less than 0.05. On the contrary, if the P-value was larger than 0.05, change was defined as non-significant.

### Demographic and lifestyle determinants of mortality risk

We examined the association of each covariate and mortality in age- and sex-adjusted Cox regression models and fully-adjusted models. As a well-known risk factor, age (continuous) had an HR (95% CI) of 1.07 (1.07, 1.08) in fully-adjusted models. Another well-studied predictor of health outcomes, gender, showed an HR (95% CI) of 0.80 (0.72, 0.89), with females showing superior health outcomes. Furthermore, we noticed that participants who were married and living with their spouses outlived their counterparts (Table 5).

**Table 5:** HRs and 95% CIs for association between all-cause mortality and health risk factors in adjusted Cox models.

Factors	Model 1:Age + Sex			Model 2: Model 1 + Demographics + SES + lifestyle			Model 3: Model 2 + Accessibility to public facilities		
	n	HR (95% CI)	P value	n	HR (95% CI)	P value	n	HR (95% CI)	P value
**Age**	3818	1.082 (1.077, 1.086)	<0.001	3781	1.076 (1.071, 1.081)	<0.001	3781	1.077 (1.071, 1.081)	<0.001
**Sex**									
Male	1602	Ref		1589	Ref		1589	Ref	
Female	2216	0.831 (0.769, 0.899)	<0.001	2192	0.769 (0.695, 0.852)	<0.001	2192	0.772 (0.697, 0.855)	<0.001
**Ethnicity**		*Not adjusted*							
Han				3711	Ref		3711	Ref	
Other				70	1.121 (1.090, 1.356)	0.409	70	1.130 (0.859, 1.486)	0.383
**Marriage**		*Not adjusted*							
Married and living with spouse				1075	Ref		1075	Ref	
Other				2706	1.216 (1.090, 1.356)	<0.001	2706	1.210 (1.085, 1.351)	<0.001
**Residence**		*Not adjusted*							
Inner-city				2394	Ref		2394	Ref	
Suburb				1387	1.086 (0.997, 1.182)	0.058	1387	1.032 (0.935, 1.139)	0.852
**Household income (RMB)**		*Not adjusted*							
<5000				1445	Ref		1445	Ref	
5000–15000				1429	0.974 (0.886, 1.070)	0.576	1429	0.982 (0.894, 1.080)	0.848
>15,000				591	1.034 (0.918, 1.164)	0.583	591	1.044 (0.926, 1.176)	0.415
**Occupation**		*Not adjusted*							
Non-manual				394	Ref		394	Ref	
Other				3387	1.032 (0.878, 1.213)	0.701	3387	1.022 (0.869, 1.201)	0.864
**Education**		*Not adjusted*							
0 year				2093	Ref		2093	Ref	
1–6 years				1191	1.008 (0.914, 1.111)	0.877	1191	1.015 (0.921, 1.120)	0.758
>6 years				497	0.886 (0.756, 1.039)	0.136	497	0.902 (0.769, 1.058)	0.174
**Smoking**		*Not adjusted*							
Current				762	Ref		762	Ref	
Former				1191	1.136 (0.998, 1.293)	0.053	1191	1.147 (1.008, 1.307)	0.043
Never				497	0.961 (0.858, 1.077)	0.496	497	0.966 (0.861, 1.083)	0.617
**Alcohol**		*Not adjusted*							
Current				763	Ref		763	Ref	
Former				455	1.155 (1.006, 1.325)	0.041	455	1.157 (1.008, 1.328)	0.036
Never				2563	1.097 (0.991, 1.215)	0.073	2563	1.100 (0.994, 1.218)	0.072
**Exercise**		*Not adjusted*							
Current				1373	Ref		1373	Ref	
Former				414	1.462 (1.286, 1.662)	<0.001	414	1.469 (1.292, 1.671)	<0.001
Never				1994	1.323 (1.212, 1.444)	<0.001	1994	1.318 (1.206, 1.439)	<0.001
**Count in 5 km-radius buffer (unit** = **150)**		*Not adjusted*			*Not adjusted*				
Any medicine-related facilities							3781	0.997 (0.985, 1.001)	0.703
Any sports and leisure service-related places							3781	0.998 (0.986, 1.010)	0.584
Any scenic spots-related places							3781	1.001 (0.981, 1.021)	0.950
**NDVI (per 0.1-unit increase)**		*Not adjusted*			*Not adjusted*			*Not adjusted*	
**NDVI change**		*Not adjusted*			*Not adjusted*			*Not adjusted*	
No change									
Increase									
Decrease									
**PM**_**2.5**_**(10 μg/m**^**3**^**)**		*Not adjusted*			*Not adjusted*			*Not adjusted*	
**PM**_**2.5**_**change**		*Not adjusted*			*Not adjusted*			*Not adjusted*	
No change									
Increase									
Decrease									
**NO**_**2**_**(10 μg/m**^**3**^**)**		*Not adjusted*			*Not adjusted*			*Not adjusted*	
**NO2 change**		*Not adjusted*			*Not adjusted*			*Not adjusted*	
NO change									
Increase									
Decrease									
**O**_**3**_**(10 μg/m**^**3**^**)**		*Not adjusted*			*Not adjusted*			*Not adjusted*	
**O**_**3**_**change**		*Not adjusted*			*Not adjusted*			*Not adjusted*	
No change									
Increase									
Decrease									

Factors	Model 4: Model 3 + Cumulative annual NDVI			Model 5: Model 4 + Air pollutants		
	n	HR (95% CI)	P value	n	HR (95% CI)	P value
**Age**	3732	1.077 (1.071, 1.081)	<0.001	3629	1.072 (1.067, 1.078)	<0.001
**Sex**						
Male	1567	Ref		1513	Ref	
Female	2165	0.782 (0.705, 0.868)	<0.001	2116	0.800 (0.720, 0.890)	<0.001
**Ethnicity**						
Han	3663	Ref		3561	Ref	
Other	69	1.118 (0.847, 1.474)	0.431	68	1.123 (0.851, 1.481)	0.413
**Marriage**						
Married and living with spouse	1064	Ref		1023	Ref	
Other	2668	1.214 (1.087, 1.356)	<0.001	2606	1.204 (1.076, 1.347)	0.001
**Residence**						
Inner-city	2357	Ref		2288	Ref	
Suburb	1375	1.023 (0.926, 1.130)	0.654	1341	1.049 (0.946, 1.163)	0.362
**Household income (RMB)**						
<5000	1433	Ref		1410	Ref	
5000–15000	1413	0.980 (0.891, 1.078)	0.678	1354	0.980 (0.889, 1.081)	0.692
>15, 000	573	1.028 (0.911, 1.161)	0.649	559	1.044 (0.923, 1.180)	0.497
**Occupation**						
Non-manual	390	Ref		369	Ref	
Other	3342	1.014 (0.861, 1.194)	0.869	3260	1.022 (0.866, 1.207)	0.794
**Education**						
0 year	2067	Ref		2030	Ref	
1–6 years	1173	1.017 (0.921, 1.123)	0.735	1136	1.026 (0.928, 1.135)	0.615
>6 years	492	0.914 (0.778, 1.073)	0.272	463	0.925 (0.786, 1.088)	0.346
**Smoking**						
Current	756	Ref		737	Ref	
Former	606	1.140 (0.999, 1.300)	0.051	591	1.148 (1.005, 1.312)	0.042
Never	2370	0.968 (0.862, 1.086)	0.574	2301	0.954 (0.849, 1.072)	0.429
**Alcohol**						
Current	757	Ref		739	Ref	
Former	448	1.171 (1.019, 1.346)	0.026	434	1.138 (0.987, 1.311)	0.075
Never	2527	1.092 (0.985, 1.209)	0.093	2456	1.075 (0.970, 1.193)	0.169
**Exercise**						
Current	1355	Ref		1311	Ref	
Former	404	1.470 (1.291, 1.675)	<0.001	392	1.419 (1.244, 1.620)	<0.001
Never	1973	1.326 (1.213, 1.449)	<0.001	1926	1.332 (1.217, 1.458)	<0.001
**Count in 5 km-radius buffer (unit** = **150)**						
Any medicine-related facilities	3732	0.985 (0.971, 1.000)	0.450	3629	0.979 (0.964, 0.994)	0.006
Any sports and leisure service-related places	3732	1.000 (0.971, 1.000)	0.945	3629	1.001 (0.987, 1.014)	0.918
Any scenic spots-related places	3732	1.004 (0.983, 1.025)	0.721	3629	1.001 (0.981, 1.023)	0.902
**NDVI (per 0.1-unit increase)**	3732	0.593 (0.367, 0.956)	0.032	3629	1.005 (0.587, 1.720)	0.985
**NDVI change**		*Not adjusted*			*Not adjusted*	
No change						
Increase						
Decrease						
**PM**_**2.5**_**(10 μg/m**^**3**^**)**		*Not adjusted*		3629	1.019 (1.015, 1.024)	<0.001
**PM**_**2.5**_**change**		*Not adjusted*			*Not adjusted*	
No change						
Increase						
Decrease						
**NO**_**2**_**(10 μg/m**^**3**^**)**		*Not adjusted*		3629	1.006 (0.999, 1.013)	0.104
**NO**_**2**_**change**		*Not adjusted*			*Not adjusted*	
NO change						
Increase						
Decrease						
**O**_**3**_**(10 μg/m**^**3**^**)**		*Not adjusted*		3629	1.002 (0.996, 1.009)	0.543
**O**_**3**_**change**		*Not adjusted*			*Not adjusted*	
No change						
Increase						
Decrease						

Factors	Model 6: Model 3 + NDVI change			Model 7: Model 6 + Air pollutants change		
	n	HR (95% CI)	P value	n	HR (95% CI)	P value
**Age**	2302	1.076 (1.070, 1.083)	<0.001	1051	1.077 (1.068, 1.087)	<0.001
**Sex**						
Male	967	Ref		428	Ref	
Female	1355	0.759 (0.665, 0.867)	<0.001	623	0.682 (0.556, 0.836)	<0.001
**Ethnicity**						
Han	2261	Ref		1039	Ref	
Other	41	1.026 (0.713, 1.475)	0.892	12	0.654 (0.322, 1.327)	0.240
**Marriage**						
Married and living with spouse	659	Ref		303	Ref	
Other	1643	1.164 (1.012, 1.339)	0.033	748	1.238 (1.009, 1.519)	0.041
**Residence**						
Inner-city	1470	Ref		646	Ref	
Suburb	832	1.015 (0.893, 3.739)	0.821	405	1.108 (0.910, 1.350)	0.307
**Household income (RMB)**						
<5000	895	Ref		463	Ref	
5000–15000	872	0.953 (0.842, 1.078)	0.444	360	0.964 (0.804, 1.157)	0.694
>15,000	346	1.062 (0.908, 1.243)	0.450	150	1.121 (0.892, 1.409)	0.326
**Occupation**						
Non-manual	226	Ref		76	Ref	
Other	2076	1.074 (0.869, 1.328)	0.508	975	1.107 (0.783, 1.565)	0.564
**Education**						
0 year	1267	Ref		624	Ref	
1–6 years	735	0.964 (0.848, 1.095)	0.570	321	0.923 (0.758, 1.124)	0.423
>6 years	300	0.916 (0.745, 1.127)	0.408	106	1.196 (0.864, 1.655)	0.281
**Smoking**						
Current	477	Ref		242	Ref	
Former	394	1.045 (0.888, 1.231)	0.594	179	1.306 (1.032, 1.654)	0.026
Never	1431	0.928 (0.806, 1.068)	0.297	630	1.065 (0.866, 1.309)	0.551
**Alcohol**						
Current	469	Ref		222	Ref	
Former	279	1.211 (1.014, 1.446)	0.035	139	1.391 (1.078, 1.795)	0.011
Never	1554	1.134 (0.993, 1.295)	0.064	690	1.375 (1.129, 1.673)	0.002
**Exercise**						
Current	835	Ref		379	Ref	
Former	258	1.503 (1.272, 1.775)	<0.001	102	1.298 (0.994, 1.695)	0.055
Never	1209	1.359 (1.213, 1.775)	<0.001	570	1.212 (1.026, 1.432)	0.024
**Count in 5 km-radius buffer (unit** = **150)**						
Any medicine-related facilities	2302	0.996 (0.981, 1.012)	0.628	1051	0.992 (0.968, 1.017)	0.540
Any sports and leisure service-related places	2302	0.996 (0.980, 1.013)	0.658	1051	1.003 (0.972, 1.034)	0.865
Any scenic spots-related places	2302	0.995 (0.981, 1.022)	0.708	1051	0.999 (0.943, 1.059)	0.977
**NDVI (per 0.1-unit increase)**		*Not adjusted*			*Not adjusted*	
**NDVI change**						
No change	922	Ref		478	Ref	
Increase	1316	0.975 (0.865, 1.100)	0.681	541	1.087 (0.909, 1.301)	0.362
Decrease	64	0.998 (0.754, 1.321)	0.989	32	0.820 (0.540, 1.244)	0.350
**PM**_**2.5**_**(10 μg/m**^**3**^**)**		*Not adjusted*			*Not adjusted*	
**PM**_**2.5**_**change**		*Not adjusted*				
No change				1049	Ref	
Increase				0	NA	NA
Decrease				2	1.828 (0.445, 7.506)	0.403
**NO**_**2**_**(10 μg/m**^**3**^**)**		*Not adjusted*			*Not adjusted*	
**NO**_**2**_**change**		*Not adjusted*				
NO change				814	Ref	
Increase				188	1.189 (0.950, 1.487)	0.130
Decrease				49	1.369 (0.954, 1.965)	0.088
**O**_**3**_**(10 μg/m**^**3**^**)**		*Not adjusted*			*Not adjusted*	
**O**_**3**_**change**		*Not adjusted*				
No change				467	Ref	
Increase				384	1.236 (0.852, 1.792)	0.265
Decrease				200	1.122 (0.883, 1.425)	0.347

In Model 1, we only included continuous age and sex. Model 2 was further adjusted for all covariates that could be potential confounders or predictors of mortality: ethnicity, education, occupation, marital status, annual household income, smoking status, alcohol consumption, and exercise. In Model 3, we added the counts of three main POI in 5 km radius on the basis of model 2. In model 4 and 6, cumulative annual NDVI and trends of NDVI were included as one independent variable respectively. In model 5 and 7, air pollutants and their changing tendency were added into the model on the basis of model 4 and model 6 respectively.

### POI and mortality risk

The association of accessibility to public facilities on all-cause mortality was evaluated. In models fully adjusted for covariates and environmental exposure variables, we found a robust association of medicine-related facilities with mortality, with continuous counts (unit = 150) showing an HR of 0.979 (95% CI: 0.964, 0.994) (Table 5). Also, the highest quartile (count >2261) of medicine-related places had an HR of 0.65 (95% CI: 0.47, 0.91) compared with the lowest quartile (count ≤ 47). The highest quartile (count >2641) of sports and leisure-related places had 36% lower death risk (HR:0.65, 95% CI: 0.46, 0.90) when using the lowest quartile (count ≤ 46) as reference (Fig. 4-1, Table 6). Accessibility to public facilities affected participants' health differently depending on where they lived. For Beijing and Guangzhou participants, scenic spot-related places presented a protective association. In Shanghai and Tianjin, sports and leisure-related places were associated with lower mortality risk. Nonetheless, the other cities in our study did not demonstrate a significant association between the three main categories of public facilities and mortality (Fig. 4-2, Table 6). As most Chinese megacities have urban sprawl, we explored health disparities. We found that elderly inhabitants of Beijing, Shanghai, and Tianjin who reside in more urban places likely city centers with higher road density had lower mortality rates. However, road density was not significantly associated with mortality in Chongqing, Guangzhou and Chengdu.

**Table 6:** HRs and 95% CIs for association between all-cause mortality and health risk factors in adjusted Cox models for overall participants and participants in each city.

Characteristics	Overall		Beijing		Shanghai		Tianjin	
	HR (95% CI)	P value	HR (95% CI)	P value	HR (95% CI)	P value	HR (95% CI)	P value
**Age**	1.071 (1.066, 1.077)	<0.001	1.083 (1.068, 1.099)	<0.001	1.080 (1.067, 1.094)	<0.001	1.110 (1.084, 1.137)	<0.001
**Sex**								
Male	Ref		Ref		Ref		Ref	
Female	0.801 (0.721, 0.891)	<0.001	0.796 (0.588, 1.076)	0.138	0.817 (0.646, 1.032)	<0.001	0.750 (0.479, 1.173)	0.207
**Ethnicity**								
Han	Ref		Ref		Ref		Ref	
Other	1.070 (0.810, 1.413)	0.634	1.099 (0.740, 1.633)	0.640	1.911 (0.932, 3.917)	0.077	3.101 (0.955, 10.066)	0.060
**Marriage**								
Married and living with spouse	Ref		Ref		Ref		Ref	
Other	1.217 (1.088, 1.361)	0.001	1.163 (0.848, 1.596)	0.348	1.271 (0.958, 1.686)	0.097	0.790 (0.489, 1.274)	0.333
**Residence**								
Inner-city	Ref		Ref		Ref		Ref	
Suburb	0.968 (0.865, 1.084)	0.576	1.205 (0.670, 2.168)	0.534	1.026 (0.761, 1.385)	0.865	0.513 (0.129, 2.051)	0.345
**Household income (RMB)**								
<5000	Ref		Ref		Ref		Ref	
5000–15000	0.982 (0.891, 1.084)	0.724	0.970 (0.704, 1.339)	0.855	1.051 (0.799, 1.383)	0.720	1.443 (0.951, 2.188)	0.085
>15,000	1.080 (0.954, 1.222)	0.223	1.262 (0.876, 1.818)	0.212	0.924 (0.642, 1.329)	0.670	1.268 (0.696, 2.309)	0.438
**Occupation**								
Non-manual	Ref		Ref		Ref		Ref	
Other	1.022 (0.866, 1.207)	0.795	0.977 (0.673, 1.419)	0.904	1.041 (0.759, 1.428)	0.802	0.947 (0.440, 2.037)	0.889
**Education**								
0 year	Ref		Ref		Ref		Ref	
1–6 years	1.025 (0.926, 1.134)	0.638	1.043 (0.775, 1.404)	0.781	1.144 (0.922, 1.418)	0.221	1.300 (0.861, 1.964)	0.212
>6 years	0.917 (0.779, 1.080)	0.299	0.932 (0.632, 1.375)	0.724	1.012 (0.742, 1.380)	0.942	0.926 (0.470, 1.822)	0.823
**Smoking**								
Current	Ref		Ref		Ref		Ref	
Former	1.142 (1.000, 1.305)	0.051	1.400 (1.024, 1.915)	0.055	0.764 (0.525, 1.111)	0.159	1.471 (0.882, 2.455)	0.139
Never	0.956 (0.850, 1.075)	0.448	1.366 (1.056, 1.767)	0.444	0.758 (0.542, 1.060)	0.106	1.058 (0.659, 1.699)	0.816
**Alcohol**								
Current	Ref		Ref		Ref		Ref	
Former	1.125 (0.976, 1.298)	0.104	1.447 (0.992, 2.110)	0.868	1.012 (0.684, 1.498)	0.953	1.130 (0.590, 2.165)	0.712
Never	1.065 (0.960, 1.181)	0.235	1.142 (0.812, 1.606)	0.890	0.976 (0.749, 1.271)	0.857	1.382 (0.863, 2.214)	0.179
**Exercise**								
Current	Ref		Ref		Ref		Ref	
Former	1.432 (1.254, 1.634)	<0.001	1.438 (1.053, 1.963)	0.022	1.827 (1.369, 2.438)	<0.001	1.385 (0.799, 2.404)	0.246
Never	1.359 (1.241, 1.487)	<0.001	1.276 (0.986, 1.651)	0.064	1.880 (1.511, 2.339)	<0.001	1.616 (1.050, 2.486)	0.029
**Count in 5 km-radius buffer**								
Any medicine-related facilities								
Q1	Ref		Ref		Ref		Ref	
Q2	0.908 (0.776, 1.063)	0.229	0.880 (0.208, 3.723)	0.862	1.334 (0.575, 3.093)	0.502	0.724 (0.390, 1.344)	0.306
Q3	0.733 (0.565, 0.951)	0.019	0.512 (0.111, 2.367)	0.391	1.717 (0.580, 5.082)	0.329	5.601 (0.502, 62.443)	0.161
Q4	0.654 (0.469, 0.912)	0.012	0.391 (0.081, 1.894)	0.243	2.274 (0.680, 7.599)	0.182	5.919 (0.455, 77.055)	0.174
Any sports and leisure service-related places								
Q1	Ref		Ref		Ref		Ref	
Q2	0.906 (0.770, 1.067)	0.236	0.512 (0.118, 2.224)	0.371	0.738 (0.333, 1.634)	0.453	0.832 (0.359, 1.931)	0.669
Q3	0.742 (0.563, 0.977)	0.033	0.568 (0.126, 2.558)	0.461	0.306 (0.109, 0.863)	0.025	0.072 (0.004, 1.228)	0.069
Q4	0.644 (0.461, 0.899)	0.010	0.825 (0.181, 3.767)	0.804	0.357 (0.110, 1.155)	0.085	0.038 (0.002, 0.802)	0.036
Any scenic spots-related places								
Q1	Ref		Ref		Ref		Ref	
Q2	1.051 (0.915, 1.208)	0.481	0.548 (0.305, 0.985)	0.044	0.604 (0.380, 0.960)	0.033	0.745 (0.336, 1.655)	0.470
Q3	1.131 (0.913, 1.403)	0.260	0.893 (0.420, 1.897)	0.768	0.868 (0.419, 1.799)	0.703	0.381 (0.137, 1.058)	0.064
Q4	1.118 (0.840, 1.488)	0.444	0.948 (0.413, 2.180)	0.901	0.497 (0.209, 1.181)	0.113	0.734 (0.204, 2.638)	0.636
**NDVI (per 0.1-unit increase)**	0.974 (0.922, 1.029)	0.346	0.635 (0.474, 0.850)	0.002	1.090 (0.931, 1.275)	0.284	0.758 (0.546, 1.053)	0.099
**PM**_**2.5**_**(10 μg/m**^**3**^**)**	1.213 (1.165, 1.262)	<0.001	0.892 (0.804, 0.989)	0.030	1.184 (0.981, 1.428)	0.079	1.032 (0.813, 1.309)	0.798
**NO**_**2**_**(10 μg/m**^**3**^**)**	1.097 (1.021, 1.179)	0.012	1.228 (0.965, 1.561)	0.094	1.277 (1.110, 1.469)	0.001	0.749 (0.467, 1.202)	0.231
**O**_**3**_**(10 μg/m**^**3**^**)**	1.020 (0.955, 1.090)	0.554	0.643 (0.510, 0.812)	<0.001	0.901 (0.767, 1.058)	0.203	0.598 (0.415, 0.860)	0.006

Factors	Chongqing		Chengdu		Guangzhou	
	HR (95% CI)	P value	HR (95% CI)	P value	HR (95% CI)	P value
**Age**	1.060 (1.051, 1.070)	<0.001	1.061 (1.050, 1.073)	<0.001	1.075 (1.047, 1.103)	<0.001
**Sex**						
Male	Ref		Ref		Ref	
Female	0.772 (0.623, 0.955)	0.017	0.890 (0.71, 1.114)	0.309	0.719 (0.393, 1.316)	0.285
**Ethnicity**						
Han	Ref		Ref		Ref	
Other	0.980 (0.400, 2.398)	0.965	1.241 (0.448, 3.442)	0.678	1.336 (0.177, 10.092)	0.779
**Marriage**						
Married and living with spouse	Ref		Ref		Ref	
Other	1.190 (0.967, 1.464)	0.100	1.394 (1.096, 1.773)	0.007	0.601 (0.340, 1.062)	0.080
**Residence**						
Inner-city	Ref		Ref		Ref	
Suburb	0.910 (0.740, 1.119)	0.371	1.106 (0.846, 1.446)	0.462	0.695 (0.358, 1.348)	0.281
**Household income (RMB)**						
<5000	Ref		Ref		Ref	
5000–15000	1.028 (0.858, 1.231)	0.766	1.184 (0.956, 1.466)	0.122	0.466 (0.262, 0.832)	0.010
>15,000	1.230 (0.966, 1.566)	0.092	1.274 (0.998, 1.625)	0.052	1.266 (0.702, 2.283)	0.433
**Occupation**						
Non-manual	Ref		Ref		Ref	
Other	0.887 (0.627, 1.254)	0.497	0.797 (0.496, 1.280)	0.348	1.075 (0.437, 2.644)	0.875
**Education**						
0 year	Ref		Ref		Ref	
1–6 years	1.100 (0.902, 1.342)	0.345	0.966 (0.782, 1.193)	0.748	0.550 (0.302, 1.002)	0.051
>6 years	0.951 (0.670, 1.35)	0.777	0.794 (0.513, 1.229)	0.301	0.520 (0.213, 1.269)	0.151
**Smoking**						
Current	Ref		Ref		Ref	
Former	1.448 (1.119, 1.873)	0.005	0.990 (0.763, 1.283)	0.937	1.858 (0.971, 3.553)	0.061
Never	1.181 (0.942, 1.481)	0.150	0.787 (0.623, 0.995)	0.045	1.350 (0.750, 2.428)	0.317
**Alcohol**						
Current	Ref		Ref		Ref	
Former	1.203 (0.939, 1.542)	0.143	1.012 (0.769, 1.331)	0.932	1.390 (0.547, 3.534)	0.489
Never	1.141 (0.954, 1.365)	0.149	0.989 (0.799, 1.223)	0.916	1.003 (0.470, 2.14)	0.994
**Exercise**						
Current	Ref		Ref		Ref	
Former	1.536 (1.168, 2.02)	0.002	1.089 (0.793, 1.495)	0.600	1.788 (0.853, 3.749)	0.124
Never	1.281 (1.08, 1.519)	0.005	1.096 (0.900, 1.336)	0.363	1.408 (0.881, 2.252)	0.153
**Count in 5 km-radius buffer**						
Any medicine-related facilities						
Q1	Ref		Ref		Ref	
Q2	0.909 (0.725, 1.140)	0.409	1.008 (0.764, 1.330)	0.956	0.542 (0.103, 2.851)	0.470
Q3	1.151 (0.737, 1.798)	0.537	0.776 (0.531, 1.134)	0.190	0.611 (0.087, 4.290)	0.620
Q4	0.707 (0.408, 1.225)	0.216	1.113 (0.580, 2.137)	0.747	0.400 (0.001, 110.218)	0.749
Any sports and leisure service-related places						
Q1	Ref		Ref		Ref	
Q2	0.939 (0.748, 1.180)	0.590	1.013 (0.757, 1.355)	0.932	2.059 (0.351, 12.091)	0.424
Q3	0.745 (0.450, 1.236)	0.255	0.798 (0.523, 1.220)	0.297	2.289 (0.266, 19.681)	0.451
Q4	1.129 (0.604, 2.111)	0.705	0.461 (0.183, 1.161)	0.100	3.514 (0.064, 193.832)	0.539
Any scenic spots-related places						
Q1	Ref		Ref		Ref	
Q2	1.070 (0.869, 1.318)	0.525	0.976 (0.743, 1.282)	0.86	0.860 (0.365, 2.027)	0.730
Q3	0.951 (0.710, 1.273)	0.734	1.306 (0.921, 1.851)	0.134	0.258 (0.072, 0.923)	0.037
Q4	0.974 (0.683, 1.389)	0.885	1.786 (0.880, 3.627)	0.109	0.148 (0.002, 10.641)	0.381
**NDVI (per 0.1-unit increase)**	0.926 (0.848, 1.011)	0.088	1.216 (1.066, 1.387)	0.004	0.822 (0.616, 1.097)	0.183
**PM**_**2.5**_**(10 μg/m**^**3**^**)**	1.491 (1.360, 1.635)	<0.001	1.439 (1.312, 1.578)	<0.001	0.907 (0.537, 1.535)	0.717
**NO**_**2**_**(10 μg/m**^**3**^**)**	0.739 (0.548, 0.997)	0.048	0.789 (0.636, 0.980)	0.032	1.473 (0.833, 2.604)	0.183
**O**_**3**_**(10 μg/m**^**3**^**)**	1.243 (1.079, 1.432)	0.003	1.063 (0.900, 1.254)	0.473	0.465 (0.288, 0.750)	0.002

We constructed seven fully-adjusted Cox regression models to assess the association between all-cause mortality and demographic, socioeconomic, lifestyle, cumulative annual greenness, air pollution and nearby facilities (by quartiles of counts in 5 km-radius buffer) in the Cox model for overall participants and participants in each city.

### Environmental determinants of mortality risk

In assessing air pollution and green space mortality risks, each 10 μg/m^3^ increase in PM_2.5_ was linked to a 2% (HR = 1.02, 95% CI: 1.02, 1.02) higher rate of death, after adjusted for demographic, socioeconomic factors and public facilities. Changes in exposure of NDVI and air pollution did not appear to have an impact on mortality as compared with stable levels (Table 5). In models that examined POIs by quartiles of counts in 5 km-radius buffer, each 10 μg/m^3^ increase in PM_2.5_ and NO_2_ were associated with a 21% (HR = 1.21, 95% CI: 1.17, 1.26) and 10% (HR = 1.10, 95% CI: 1.02, 1.18) higher risk of death. Nonetheless, the mortality HRs for NDVI and ozone were not significant (Fig. 4-1, Table 6). Effect sizes of natural environment exposure variables also showed diverse results in the six megacities (Fig. 4-2, Table 6). We also acquired the mortality HRs for the above risk factors in age- and sex-adjusted models (Supplementary Table S2). The relationship between mortality and baseline year nighttime light was assessed. Greater night-time light level was associated with a better health outcome in both age-and sex-adjusted models and models fully adjusted for covariates, public facilities, NDVI, and air pollutants overall, with HRs of 0.998 (95% CI: 0.997, 0.999) and 0.995 (95% CI: 0.992, 0.999) respectively (Table 7). We calculated the Pearson correlation coefficients between nighttime light and other health risk factors (Supplementary Table S3). We also calculated the risk ratios, prevalence, and unweighted PAFs for all-cause mortality associated with risk factors (Supplementary Figure S5, Supplementary Table S4).

**Table 7:** HRs and 95% CIs for association between all-cause mortality and nighttime light in age-sex adjusted and fully adjusted Cox models.

Nighttime light	Model 1: Age + Sex		Model 2: Fully adjusted	
	HR (95% CI)	P value	HR (95% CI)	P value
Total	0.998 (0.997, 0.999)	0.005	0.995 (0.992, 0.999)	0.040
City				
Beijing	0.993 (0.989, 0.998)	0.004	0.994 (0.984, 1.005)	0.297
Shanghai	0.999 (0.995, 1.003)	0.503	0.993 (0.984, 1.002)	0.136
Tianjin	0.991 (0.985, 0.997)	0.003	1.006 (0.987, 1.024)	0.548
Chongqing	1.001 (0.997, 1.004)	0.781	1.009 (0.999, 1.019)	0.095
Chengdu	0.998 (0.995, 1.002)	0.313	1.007 (0.998, 1.016)	0.152
Guangzhou	1.000 (0.990, 1.010)	0.940	1.004 (0.985, 1.023)	0.706

Model 1 is adjusted for age and sex. Model 2 was adjusted for age, sex, ethnicity, marital status, occupation, education level, urban or rural residence, household income, smoking status, alcohol status, exercise, counts of medicine-related facilities, sports and leisure service-related places and scenic spots-related places in 5 km radius buffer, cumulative annual greenness, last-year PM_2.5_, last-year NO_2_ and last-year ozone.

## Discussion

Our findings confirmed that many urban attributes can lead to longer survival in this prospective cohort of elderly residents. In our model, each additional year of increase in age is associated with a 7% increase in mortality risk. Living closer to the city center benefits residents in Beijing, Tianjin, and Shanghai, but not necessarily in Chongqing, Chengdu, and Guangzhou, which have more polycentric layouts. In Beijing, living in the suburbs compared to living in the downtown is associated with a 47.6% increase in mortality risk, equivalent to 6.8 years age. Environmental pollution is associated with higher mortality between and within cities. In Chengdu, the area-average green space NDVI from 2000 to 2021 varied from 0.27 in the inner city to 0.63 in the city's outskirts, coupled with corresponding large variations in air pollution (64.4–46.9 μg/m^3^ for PM_2.5_). The heterogeneity within these environmental factors led to variations in mortality risk effect estimates in accordance with previous research,^37^, ^38^, ^39^, ^40^, ^41^, ^42^ but our findings provided a holistic assessment of large variations of exposure in a city. Environmental pollution generally decreases in southern China, with Guangzhou exhibiting relatively cleaner air, and more green space. Living near public facilities in the general vicinity, not necessarily within walking distance, is associated with lower mortality risk. With a 5 km distance, a density of at least 520 medicine-related facilities is associated with over 26.7% lower mortality risk, compared to the lowest quartile (less than 47 medicine-related facilities). We note that though inner-city residents had higher SES compared with outskirts residents, people living in the innermost areas in each city were not always associated with the highest SES.

### Novel findings

Our study's unique contribution lies in evaluating multiple environmental risk factors using population health and ecological data, noting their spatial and temporal trends. We observed interesting patterns in PM_2.5_ concentration, which typically followed an inverse-"U" or “M" shape over time. Prior research identified three phases in the trend from 2000 to 2019: an increase (2000–2007), a slight decline (2008–2012), and finally, a sharp rise followed by a steady decline.^43^ This trend reflects the interplay of emission regulations and meteorological variations.^43^^,^^44^ Notably, PM_2.5_ distribution in Tianjin diverged from the city center concentration observed in other megacities. This may be attributed to its unique industrial history and resulting urban development pattern.^45^^,^^46^ However, understanding air pollution concentrations requires considering additional factors such as the environment, automobile exhaust, and pollution from neighboring cities, which warrants further investigation to inform effective policy decisions.

### Intra-city and inter-city disparities

Existing research has observed the heterogeneous urban development and its association with health. Since 1980s, an extensive amount of literature has demonstrated the association between health outcomes and city resources, with higher-SES individuals and communities generally having better access to public resources and enjoying better health.^47^, ^48^, ^49^, ^50^, ^51^, ^52^ In our population, the findings of the protective association of public facilities on mortality were similar to prior studies. However, contrary to developed economies, city center residents in China lived in areas with lower air quality, less greenness coverage, easier access to public facilities and higher SES, mainly demonstrated by higher household income since income is an important indicator of SES.^53^ Consequently, how the contradictory impact of social and physical environment affect health within the same city in developing countries was worthy of scholarly attention. A study focusing on Latin American cities noted that sub-city-level intersection density and population density were positively related to obesity and diabetes, while green space was negatively associated.^54^ A negative association of urban built environment density on health was also proved in China.^55^ These findings were evidence that physical environment might overshadow the health advantages of city resources in some cases. Nevertheless, in Chinese megacities, we found that elderly residents in Beijing, Tianjin, and Shanghai living in city centers had lower mortality, whereas ring road areas were not significantly associated with mortality in Chongqing, Guangzhou, and Chengdu. We assume that the health benefit of city resources may outweigh the harm from air pollution and insufficient green space in Beijing, Tianjin, and Shanghai since they have greater SES disparity due to higher levels of urbanization and GDP.^56^ In contrast, in cities like Guangzhou, Chongqing, and Chengdu, SES disparities among residents living near ring roads were less pronounced. We assume the trade-offs created an equilibrium for health outcomes.

### Polycentric Urbanism Health Impact

The nexus of urban health outcomes extends beyond mere proximity to a city's geographic center. Our study, leveraging nighttime light data as a proxy for urban activity, reveals an intriguing pattern: Beijing, Guangzhou, and Shanghai, while each possessing a primary urban center, also feature multiple sub-centers. This spatial distribution is particularly distinct in Guangzhou and Shanghai, where urban centers are more dispersed compared to the more centralized Beijing. Complementing these findings, an analysis based on population density corroborates that Chongqing and Chengdu exhibit a more pronounced polycentric structure, with greater distances between their sub-centers and main centers, as compared to Beijing, Tianjin, and Shanghai.^57^ In our research, we created a geographical mapping of average nighttime light intensity and population density variations across these cities. Our results resonate with and build upon existing literature, delineating Beijing's predominantly monocentric layout against the more polycentric configurations of Chengdu and Chongqing. Previous research shows indoor nighttime light, or artificial light at night, may cause negative health effects, such as breast cancer, circadian phase disruption, and sleep disorders.^58^ Our ambient or outdoor nighttime light, with mostly null health risks, is perhaps a surrogate for economic vibrancy, was relatively constant within the inner ring road areas of Beijing, Shanghai, and Tianjin, but markedly diminished in the outer rings. This disparity in urban intensity could be the notable health disparities we found among residents of different urban rings, especially pronounced in Beijing, Shanghai, and Tianjin.^59^ A notable finding in our analysis was that lower mortality risks in Beijing were predominantly associated with areas between the fourth and fifth ring roads, rather than the more centrally located second ring road. This challenges conventional assumptions about urban health dynamics and may be attributed to a combination of factors including socioeconomic status, urban planning and infrastructure, healthcare access, and environmental conditions. A striking finding in our analysis was that, in Beijing, lower mortality risks were predominantly associated with areas between the fourth and fifth ring roads, rather than the more centrally located second ring road. Specifically, we observed that the second ring road had higher levels of air pollution exposure compared to the city average (PM_2.5_: 87.12 μg/m^3^ vs 81.61 μg/m^3^). Areas between the fourth and fifth ring road exhibited similar environmental exposure to the average level in Beijing, suggesting that the interplay of social and physical environments could have counteracting effects, or that these areas may attract healthier residents.

The World Health Organization (WHO)'s framework for Age-Friendly Cities and Communities, established in 2007, has gained global recognition.^60^ However, its detailed application in regions with complex socio-environmental disparities warrants further exploration. In our analysis, several variables align with WHO's domains on outdoor environments (air pollution, green space), social participation (sports and leisure service-related places), and community and health services (medicine-related facilities). While our findings suggest potential correlations between these aspects and healthy longevity within the context of Chinese megacities, they do not conclusively establish the age-friendliness of city centers compared to outskirts or places of peri-urbanization. This is due to the observed variations: city centers appear more conducive to social participation and access to health services, yet less favorable in terms of outdoor environmental quality.

### Ethnic disparities

Ethnic differences may contribute to poorer health outcomes in cities.^61^ A nationally representative survey in England found that socioeconomic status and support from local services are important determinants for poorer health outcomes of minority ethnic groups.^62^ A sizeable body of literature in China has also documented health inequalities among different ethnic groups ascribed to socioeconomic status.^63^^,^^64^ However, our study, possibly limited by sample size, presented contrasting findings. Ethnic minorities in our study, primarily residing in Beijing, were found to live in areas with less green space, higher NO_2_ and ozone exposure, and closer proximity to public facilities than the *Han* population, indicating higher socioeconomic status. Despite these, their health outcomes were not significantly better than those of the *Han* population, suggesting that ethnic minorities might not be gaining equivalent health advantages that their socioeconomic position should confer. It could suggest that they may be missing out on neighborhood benefits, such as social support, despite their urban dwelling and elevated socioeconomic status.

### Strengths

Our study leveraged a unique combination of methodologies to explore the key determinants of successful aging and the role of urban locations in mortality rates among older adults in megacities within a developing country. A notable strength of this research is the innovative use of point of interest (POI) data from AutoNavi maps, offering a granular assessment of individual-level accessibility to various health-related public facilities. Second, our study population is among the oldest, which is one of the highest-growing demographic segments but has little evidence. Additionally, our study provides ecological trends with almost two decades of data on greenness and air pollution exposure and a prospective cohort study involving 4992 older participants. The richness of the data used not only validates the results but also enables a nuanced subgroup analysis, linking socioeconomic status, POI, and remote sensing databases. The extensive, diverse datasets used and our ability to track changes over time across multiple cities contribute to a more profound understanding of urban environmental health effects on aging, and these findings can be crucial for future urban planning and health policy developments.

### Limitations

Admittedly, the generalizability of the study is subject to certain limitations. First, our sample size is comparatively small in examining the health effects in the many stratification groups, which could also be why we did not see consistent effects of environmental change as reported in prior literature. Second, we did not find the confounders that could explain the discrepant association between public facilities and mortality in age- and sex-adjusted and fully-adjusted models. Third, the deficiency of the NO_2_ and ozone data for 2001–2004 and 2000–2005 on individual levels, as well as the unavailability of ecological ozone data due to the lack of monitoring data and measurement quality, made the results of long-term effects of NO_2_ and ozone less reliable. Lastly, because of the age demographic of our study population of the oldest-olds, we do not have generalizability of our findings for those less advanced.

### Conclusion

Our study revealed disparities between urban and rural areas in China and intra-city inequalities. We found that residents in city centers, often of higher socioeconomic status, have proximity to public amenities and economic activities. Remarkably, these urban health advantages offset the urban health penalty of reduced green space and heightened air pollution. While socioeconomic factors remain a significant predictor of urban mortality, our findings underscore the considerable influence of environmental pollution and greenness on longevity in urban settings. Proximity to public facilities and economic activities is associated with health benefits, counterbalancing the negative impacts of lower green space and high air pollution. Our research suggests that polycentric city spatial development, combined with balanced infrastructure, points of interest, green spaces, and low air pollution, can create age-friendly cities that promote health.

## Contributors

John S. Ji, Jialu Song and Hui Miao conceived and designed the study idea. Hui Miao and Linxin Liu collected, pre-processed and validated the underlying data. Jialu Song conducted the statistical analyses. John S. Ji and Jialu Song led the writing processes. Dong Li, Jun Yang, Haidong Kan, and Yi Zeng contributed to resources and finding interpretation. Authors approved the final version of the manuscript.

## Data sharing statement

The data used in this study are accessible on platforms with restricted access. Vegetation data from the NDVI MODIS is available through NASA's Land Processes Distributed Active Archive Center (LP DAAC: https://lpdaac.usgs.gov). Atmospheric data include PM_2.5_ measurements, accessible at https://sites.wustl.edu/acag/datasets/surface-pm2-5/#V4.CH.03, ozone data from the China National Environmental Monitoring Center (CNEMC: http://www.cnemc.cn/), and NO2 levels from a global database at https://figshare.com/articles/dataset/Global_surface_NO2_concentrations_1990-2020/12968114. Nightlight data sources are the Version 4 DMSP-OLS Nighttime Lights Time Series (https://eogdata.mines.edu/products/dmsp/) and the Resource and Environment Science and Data Center (Annual data set of Chinese night light: https://www.resdc.cn/DOI/DOI.aspx?DOIID=105). Additionally, public facilities data are accessible via the AutoNavi Open platform (https://lbs.amap.com). Notably, NDVI and ozone data undergo secondary processing using the datasets mentioned above, with methodologies detailed in the methods section. Epidemiological health data are partly sourced from the Duke Aging Center (https://agingcenter.duke.edu/CLHLS). Data on urban population density and urban construction land for municipal utilities are derived from the China Urban and Rural Construction Statistical Yearbook. Coding is made available in GitHub and updated when necessary (https://github.com/johnjiresearchlab/MegaCity_healthy_aging).

## Editor note

The Lancet Group takes a neutral position with respect to territorial claims in published maps and institutional affiliations.

## Declaration of interests

We declare no competing interests.
