# Supplementary material for: Urban health advantage and penalty in aging populations: a comparative study across major megacities in China
Source: Lancet Reg Health West Pac. 2024 Jun 15;48:101112. doi: 10.1016/j.lanwpc.2024.101112 (PMC11228801; doi:10.1016/j.lanwpc.2024.101112)
Supplement: Supplementary Figures and Tables [file mmc1.docx]

**Supplementary Information**

**Title**

Urban Landscape and Exceptional Longevity: Ecological and Epidemiological Cohort Analysis of Six Chinese Megacities

**Authors**

Jialu Song (1,2)

Linxin Liu (1)

Hui Miao (3)

Yanjie Xia (1)

Dong Li (4)

Jun Yang (5)

Haidong Kan (6)

Yi Zeng (7,8)

John S. Ji (1,*)

**Affiliations**

1. Vanke School of Public Health, Tsinghua University, Beijing, China
2. School of Public Health, Peking University, Beijing, China
3. T.H. Chan School of Public Health, Harvard University, Boston, MA, USA
4. Institute for Urban Governance and Sustainable Development, Tsinghua University, Beijing, China
5. Department of Earth System Science, Tsinghua University, Beijing, China
6. School of Public Health, Key Laboratory of Public Health Safety of the Ministry of Education and Key Laboratory of Health Technology Assessment of the Ministry of Health, Fudan University, Shanghai, China
7. National School of Development, Peking University, Beijing, China
8. School of Medicine, Duke University, Durham, NC, USA


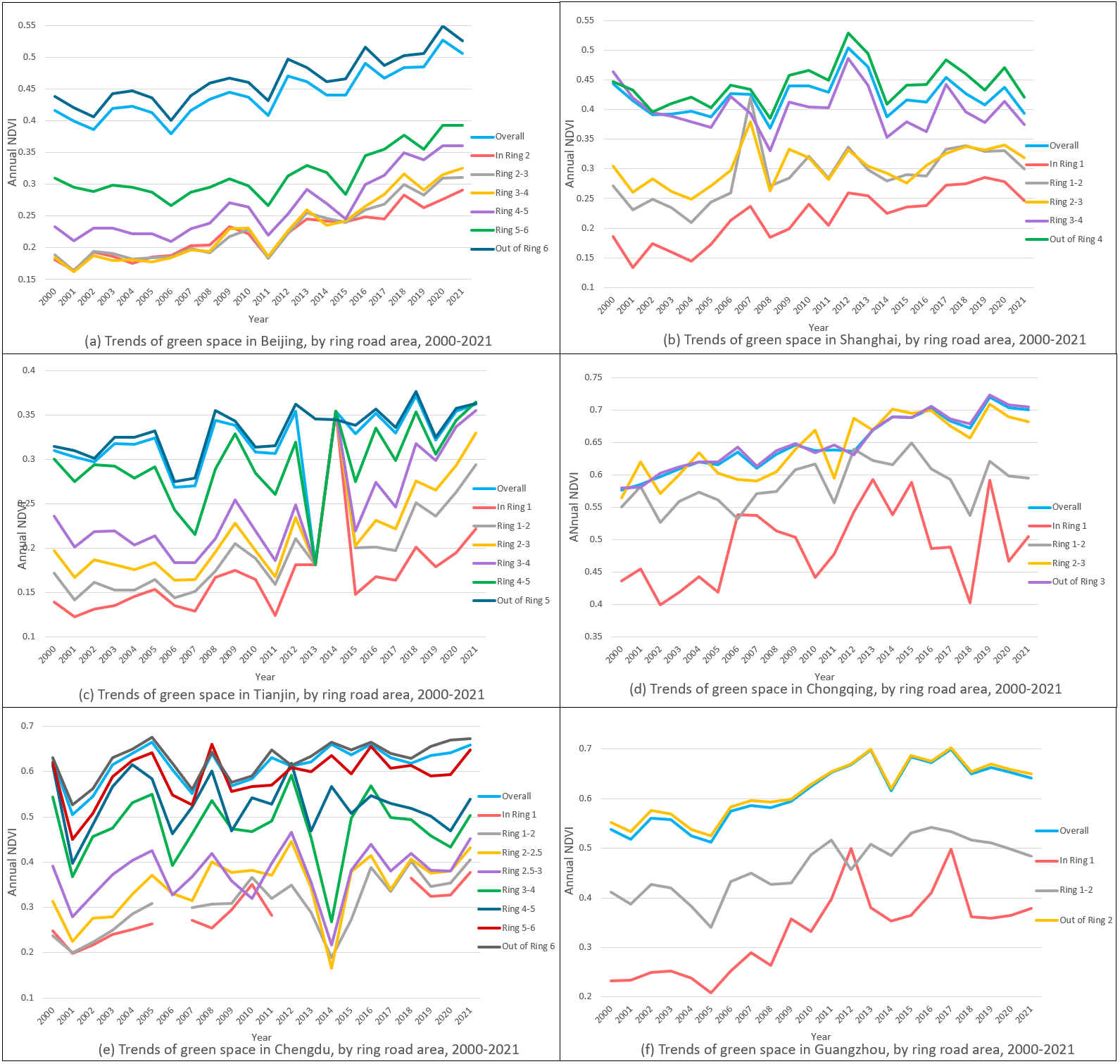


**Supplementary Figure 1. Annual average NDVI in the six megacities, by ring road areas, 2000-2021.** The lines depict the spatial-temporal distribution of green space. Data presented is annual average NDVI values in each ring road area during 2000 to 2021 in the six megacities. Our analysis of the six megacities revealed an overall upward trend in greenness levels, though with great fluctuations, and that inner areas of the city had lower NDVI than outer ones.


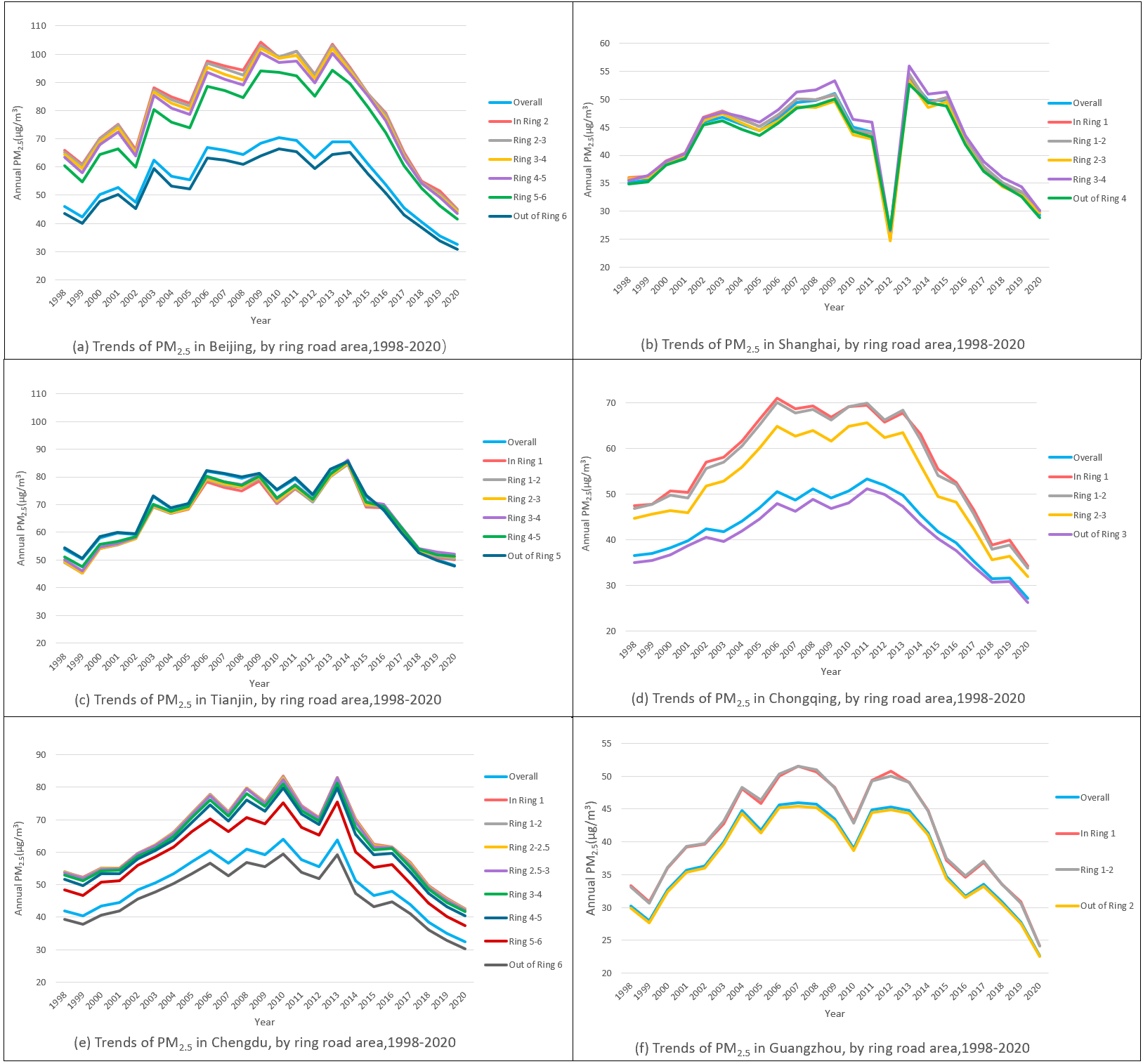


**Supplementary Figure 2. Annual average PM_2.5_ concentration in the six megacities, by ring road areas, 1998-2020.** The lines depict the spatial-temporal distribution of PM_2.5_. Data presented is annual average PM_2.5_ in each ring road area during 1998 to 2020 in the six megacities. PM_2.5_ in the six megacities first increased with fluctuation and then declined steadily, following an inverse U-shape verified by regression models. For residents in Beijing, Shanghai, Chongqing, Chengdu and Guangzhou, living in close proximity to city centers was associated with higher PM_2.5_ exposure. However, an opposite pattern was found in Tianjin.


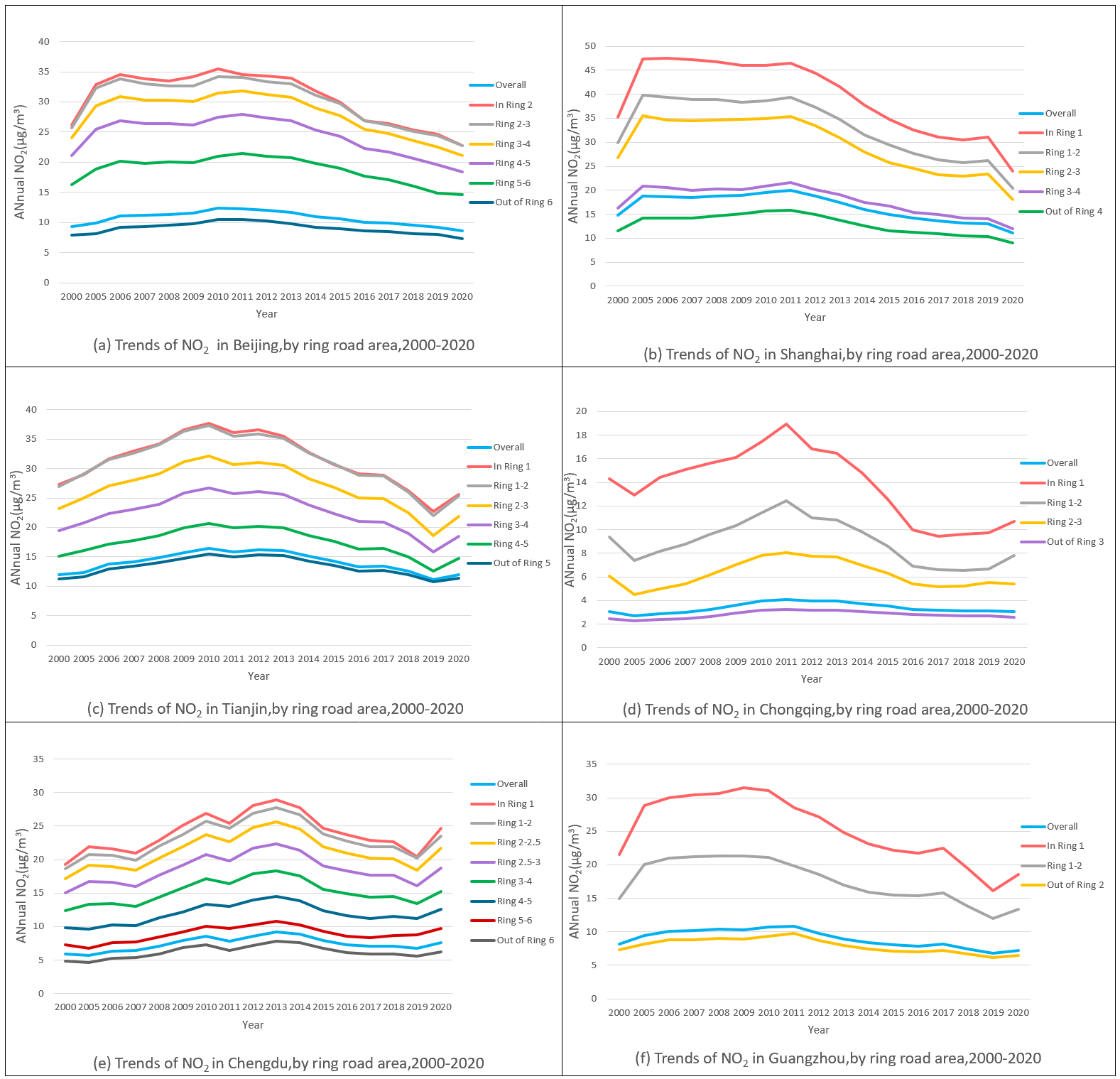


**Supplementary Figure 3. Annual average NO_2_ concentration in the six megacities, by ring road areas, 2000-2020.** The lines depict the spatial-temporal distribution of NO_2_. Data presented is annual average NO_2_ in each ring road area during 2000 to 2020 in the six megacities. The relative levels of NO_2_ showed an inverse U pattern in the city of Chengdu, Chongqing and Tianjin, while in Beijing, Shanghai and Guangzhou_,_ NO_2_ showed a decreasing trend. Highest NO_2_ level was found in the city center in all the six megacities.


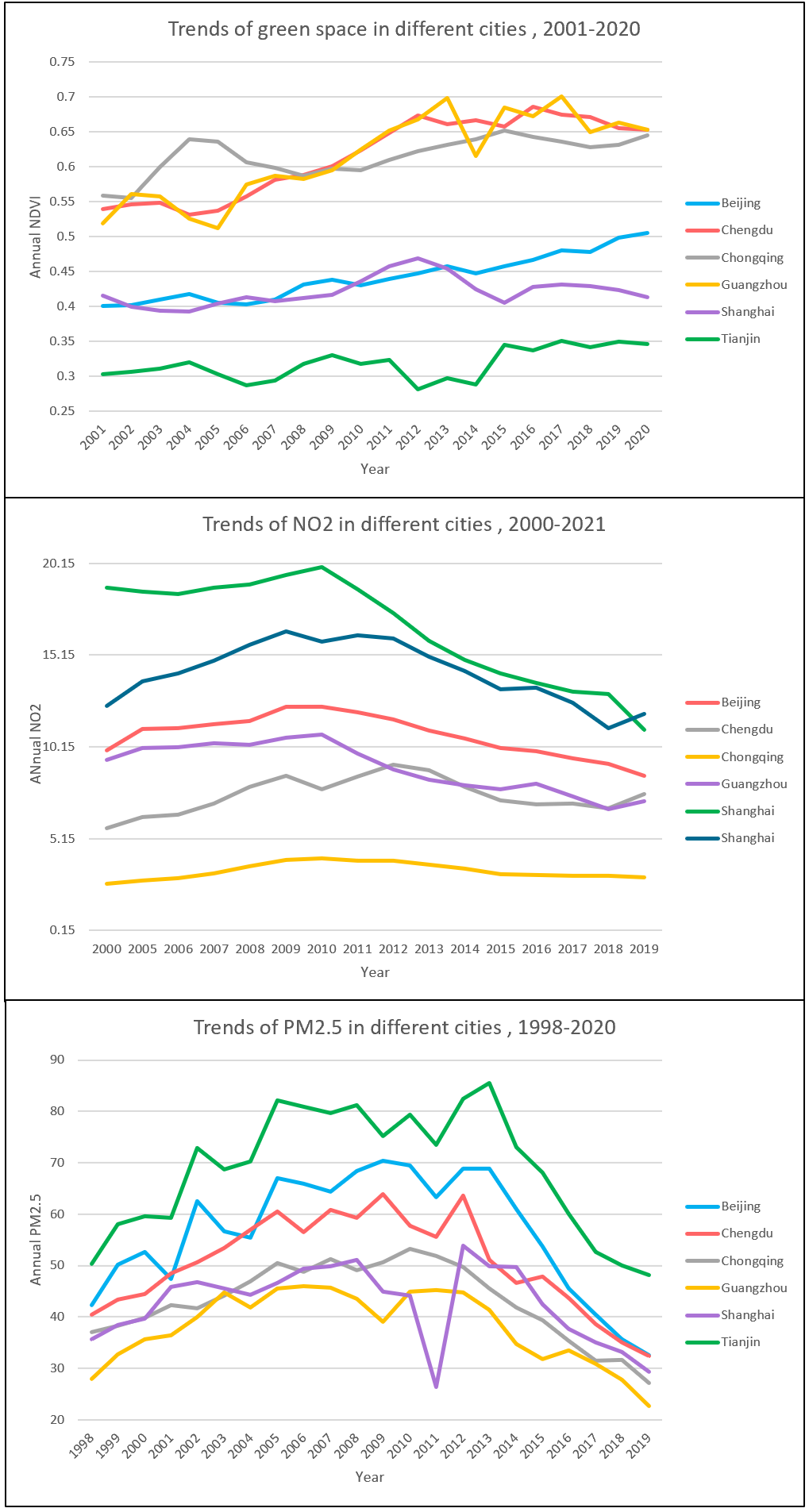


**Supplementary Figure 4. Three-year moving averages of NDVI, NO_2_ and PM_2.5_ in the six megacities.** The three figures compare the level of NDVI and air pollution in the six megacities. Among the six megacities, Tianjin had the lowest greenness level and the highest PM_2.5_ exposure, whereas Guangzhou had the lowest PM_2.5_ and high NDVI. Shanghai had the highest NO_2_ exposure among the six megacities and Chongqing had the lowest.


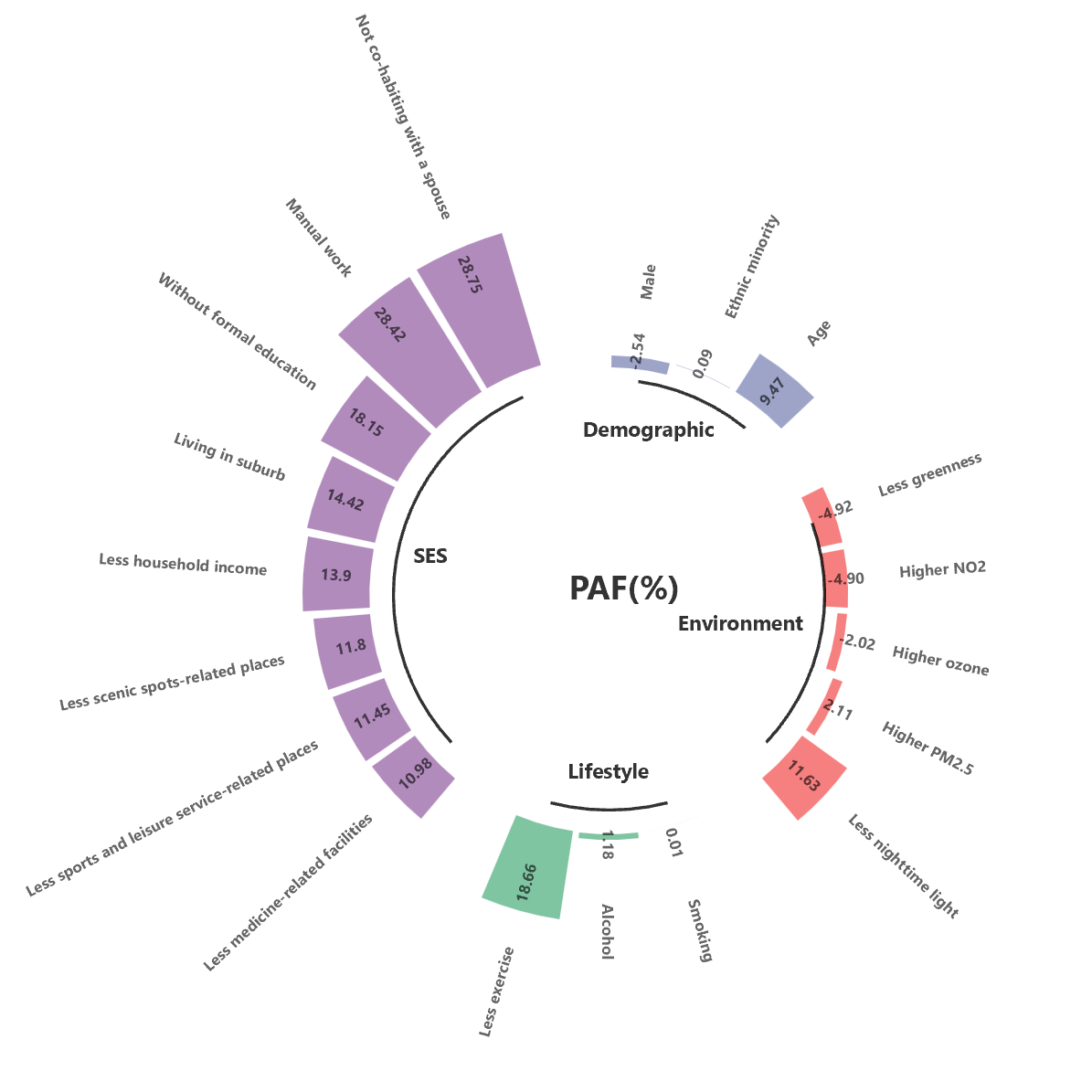


**Supplementary Figure 5. Unweighted Population attributable fractions (PAF) of mortality risk factors.** PAF indicates the fraction of mortality that could be prevented by eliminating certain risk factors from a population, which is calculated by the formula: PAF = P (RR − 1)/ (P [RR − 1] + 1), in which P is the population prevalence of a risk factor and RR is the risk ratio of that risk factor. The figure indicates that SES, lifestyle and social resources were important determinants of health, while environmental indicators made less contribution to death. As anticipated, SES, lifestyle and social resources were important determinants of health, with marriage (28.75%), occupation (28.42%), and exercise status (18.66%) associated with the greatest fraction of attributable mortality. The count of medicine-related, leisure service-related, and scenic spots-related places in a 5 km-radius buffer showed a substantial association with mortality, with PAFs of 10.98%, 11.45% and 11.80%, respectively. However, environmental indicators made less contribution to death, among which, 11.63% death can be prevented by increasing nighttime light. Corresponding numeric data can be found in Supplementary Table 4.

**Supplementary Table 1-1. Characteristics of participants, and the levels and changes of greenness in 1250 m radius buffer.** Residential NDVI in 1250 m radius was calculated by linking NDVI data with the longitude and latitude of each participants’ residential address. The cumulative annual NDVI and seasonal NDVI were the mean of the annual-average or seasonal-average NDVI during each participants’ follow-up period--from the baseline year to the death year for deceased individuals, and to the last interview year for those still alive at follow-up and those lost to follow-up. Change of NDVI was estimated by putting every year’s annual-average NDVI during each participants’ follow-up period into a linear regression model and was defined as a significant increase or decrease if the regression coefficient was positive or negative, with its p-value less than 0.05. On the contrary, if the p-value was larger than 0.05, change was defined as non-significant. The mean cumulative annual NDVI in the 1250 m radius around each participant’ s residential address was 0.38 (SD:0.19). We found that individuals aged 80-99 years or above 100 years, who were not co-habiting with a spouse, who lived in rural areas, who had less than 5000 yuan of household income, who had previously worked in manual labor, who received formal education less than one year, who were current smokers, current or former alcohol consumers and who never exercised had higher residential greenness level than the average. While some people lived in areas with lower cumulative NDVI than the average, including people aged 65-79 years, people who were male, ethnic minorities, married and living with spouse, who lived in inner-city, who had more than 5000-yuan annual household income, who had previously worked in non-manual labor, who received formal education longer than one year, who were former smokers or never smoked, never consumed alcohol, were current or former exercisers. As for greenness variation over time, 1133, 1852 and 91 participants lived with stable, increasing and decreasing NDVI respectively.

| **Characteristics** | | **Cumulative annual NDVI (1250m)** | **Cumulative spring NDVI (1250m)** | **Cumulative summer NDVI (1250m)** | **Cumulative autumn NDVI (1250m)** | **Cumulative winter NDVI (1250m)** | **NDVI change** | | | |
| --- | --- | --- | --- | --- | --- | --- | --- | --- | --- | --- |
|  |  |  |  |  |  |  | **No change** | **Increase** | **Decrease** | **Missing** |
| **Total** | | 0.38(0.19) | 0.34(0.18) | 0.47(0.20) | 0.38(0.17) | 0.25(0.16) | 1033(20.7%) | 1852(37.1%) | 91(1.8%) | 2016(40.4%) |
|  | **Range** | -0.17 to 0.81 | -0.17 to 0.78 | -0.06 to 0.98 | -0.01 to 0.80 | -0.04 to 0.69 |  |  |  |  |
|  | **Median (IQR)** | 0.36(0.20 to 0.56) | 0.34(0.17 to 0.50) | 0.48(0.27 to 0.65) | 0.37(0.22 to 0.53) | 0.19(0.11 to 0.40) |  |  |  |  |
| **Age, years** | |  |  |  |  |  |  |  |  |  |
| **Age group** | |  |  |  |  |  |  |  |  |  |
|  | **65-79 years** | 0.35(0.18) | 0.33(0.17) | 0.43(0.19) | 0.36(0.17) | 0.26(0.16) | 249(20.5%) | 469(38.5%) | 33(2.7%) | 466(38.3%) |
|  | **80-89 years** | 0.40(0.18) | 0.36(0.17) | 0.48(0.20) | 0.39(0.17) | 0.26(0.16) | 284(24.6%) | 389(33.7%) | 19(1.6%) | 463(40.1%) |
|  | **90-99 years** | 0.38(0.19) | 0.34(0.18) | 0.48(0.20) | 0.37(0.17) | 0.24(0.16) | 288(20.4%) | 498(41.0%) | 24(1.7%) | 599(42.5%) |
|  | **≥100 years** | 0.39(0.19) | 0.35(0.18) | 0.47(0.20) | 0.38(0.17) | 0.25(0.17) | 212(17.5%) | 496(41.0%) | 15(1.2%) | 488(40.3%) |
| **Sex** | |  |  |  |  |  |  |  |  |  |
|  | **Male** | 0.37(0.19) | 0.34(0.17) | 0.46(0.20) | 0.37(0.17) | 0.25(0.16) | 434(20.6%) | 780(37.1%) | 27(1.3%) | 864(41.0%) |
|  | **Female** | 0.38(0.19) | 0.35(0.18) | 0.48(0.20) | 0.38(0.17) | 0.26(0.16) | 599(20.7%) | 1072(37.1%) | 64(2.2%) | 1152(40.0%) |
| **Ethnicity** | |  |  |  |  |  |  |  |  |  |
|  | **Han Chinese** | 0.38(0.19) | 0.34(0.18) | 0.47(0.20) | 0.38(0.17) | 0.25(0.16) | 1023(20.9%) | 1802(36.9%) | 91(1.9%) | 1969(40.3%) |
|  | **Ethnic minorities** | 0.26(0.14) | 0.23(0.13) | 0.35(0.17) | 0.27(0.12) | 0.15(0.11) | 10(9.6%) | 50(48.1%) | 0(0) | 47(45.2%) |
| **Marital status** | |  |  |  |  |  |  |  |  |  |
|  | **Married and living with spouse** | 0.35(0.19) | 0.32(0.18) | 0.44(0.20) | 0.36(0.17) | 0.25(0.15) | 304(21.7%) | 521(37.2%) | 21(1.5%) | 553(39.5%) |
|  | **Other** | 0.39(0.19) | 0.35(0.18) | 0.48(0.20) | 0.38(0.17) | 0.26(0.16) | 729(20.3%) | 1331(37.1%) | 70(1.9%) | 1463(40.7%) |
| **Residence** | |  |  |  |  |  |  |  |  |  |
|  | **Inner-city** | 0.33(0.19) | 0.30(0.18) | 0.40(0.20) | 0.33(0.17) | 0.21(0.16) | 612(18.6%) | 1316(40.0%) | 43(1.3%) | 1318(40.1%) |
|  | **Suburb** | 0.45(0.16) | 0.41(0.14) | 0.57(0.14) | 0.45(0.14) | 0.31(0.13) | 421(24.7%) | 536(31.5%) | 48(2.8%) | 698(41.0%) |
| **Household income (RMB)** | |  |  |  |  |  |  |  |  |  |
|  | **<5000** | 0.46(0.16) | 0.42(0.15) | 0.55(0.17) | 0.45(0.15) | 0.34(0.15) | 431(26.3%) | 523(32.0%) | 57(3.5%) | 625(38.2%) |
|  | **5000-15000** | 0.32(0.19) | 0.29(0.18) | 0.40(0.21) | 0.32(0.17) | 0.19(0.13) | 338(18.6%) | 743(41.0%) | 15(0.8%) | 717(39.5%) |
|  | **>15000** | 0.36(0.18) | 0.31(0.18) | 0.45(0.18) | 0.36(0.15) | 0.23(0.16) | 160(17.7%) | 347(38.5%) | 5(0.6%) | 390(43.2%) |
| **City** | |  |  |  |  |  |  |  |  |  |
|  | **Beijing** | 0.24(0.08) | 0.20(0.06) | 0.36(0.14) | 0.27(0.08) | 0.13(0.04) | 19(2.3%) | 461(56.4%) | 0(0) | 338(41.3%) |
|  | **Shanghai** | 0.23(0.13) | 0.21(0.13) | 0.32(0.17) | 0.26(0.13) | 0.19(0.11) | 269(20.5%) | 501(38.2%) | 21(1.6%) | 522(39.8%) |
|  | **Tianjin** | 0.24(0.10) | 0.22(0.11) | 0.37(0.15) | 0.25(0.10) | 0.13(0.07) | 15(4.2%) | 185(51.7%) | 1(0.3%) | 157(48.9%) |
|  | **Chongqing** | 0.53(0.13) | 0.50(0.11) | 0.59(0.14) | 0.52(0.14) | 0.44(0.07) | 256(21.5%) | 410(34.4%) | 30(2.5%) | 496(37.8%) |
|  | **Chengdu** | 0.51(0.16) | 0.47(0.15) | 0.58(0.18) | 0.46(0.13) | 0.46(0.13) | 343(37.0%) | 201(20.7%) | 31(3.3%) | 353(38.0%) |
|  | **Guangzhou** | 0.35(0.14) | 0.38(0.10) | 0.46(0.13) | 0.45(0.14) | 0.34(0.13) | 131(34.2%) | 94(24.5%) | 8(2.1%) | 150(39.2%) |
| **Main occupation before 60 years of age** | |  |  |  |  |  |  |  |  |  |
|  | **Non-manual** | 0.28(0.16) | 0.25(0.16) | 0.35(0.17) | 0.29(0.15) | 0.18(0.13) | 65(11.5%) | 248(43.8%) | 2(0.4%) | 251(44.3%) |
|  | **Other** | 0.39(0.19) | 0.35(0.18) | 0.48(0.20) | 0.39(0.17) | 0.26(0.16) | 967(21.9%) | 1603(36.3%) | 89(2.0%) | 1761(39.8%) |
| **Education, years** | |  |  |  |  |  |  |  |  |  |
| **Education** | |  |  |  |  |  |  |  |  |  |
|  | **0 year** | 0.42(0.18) | 0.38(0.17) | 0.51(0.19) | 0.41(0.17) | 0.28(0.16) | 599(22.5%) | 947(35.5%) | 53(2.0%) | 1068(40.0%) |
|  | **1-6 year** | 0.35(0.19) | 0.32(0.18) | 0.44(0.20) | 0.36(0.16) | 0.24(0.16) | 322(20.8%) | 580(37.4%) | 31(2.0%) | 618(39.8%) |
|  | **>6 years** | 0.28(0.17) | 0.25(0.16) | 0.35(0.18) | 0.29(0.16) | 0.18(0.13) | 103(14.0%) | 310(42.2%) | 7(1.0%) | 315(42.9%) |
| **Smoking status** | |  |  |  |  |  |  |  |  |  |
|  | **Current** | 0.42(0.19) | 0.38(0.18) | 0.51(0.19) | 0.41(0.16) | 0.28(0.16) | 234(24.6%) | 317(33.3%) | 24(2.5%) | 376(39.5%) |
|  | **Former** | 0.36(0.18) | 0.33(0.17) | 0.45(0.20) | 0.36(0.16) | 0.24(0.15) | 165(20.3%) | 320(39.5%) | 13(1.6%) | 313(38.6%) |
|  | **Never** | 0.37(0.19) | 0.34(0.18) | 0.46(0.20) | 0.37(0.17) | 0.25(0.16) | 634(19.7%) | 1213(37.6%) | 54(1.7%) | 1325(41.1%) |
| **Alcohol status** | |  |  |  |  |  |  |  |  |  |
|  | **Current** | 0.42(0.19) | 0.38(0.18) | 0.50(0.20) | 0.41(0.17) | 0.29(0.17) | 214(22.5%) | 340(35.7%) | 14(1.5%) | 385(40.4%) |
|  | **Former** | 0.40(0.18) | 0.36(0.18) | 0.48(0.20) | 0.39(0.16) | 0.25(0.16) | 117(20.0%) | 216(36.9%) | 13(2.2%) | 239(40.9%) |
|  | **Never** | 0.36(0.19) | 0.33(0.18) | 0.45(0.20) | 0.37(0.17) | 0.24(0.16) | 701(20.3%) | 1295(37.5%) | 64(1.9%) | 1390(40.3%) |
| **Exercise** | |  |  |  |  |  |  |  |  |  |
|  | **Current** | 0.35(0.18) | 0.32(0.17) | 0.43(0.20) | 0.35(0.17) | 0.24(0.16) | 355(19.0%) | 731(39.2%) | 25(1.3%) | 753(40.4%) |
|  | **Former** | 0.31(0.17) | 0.28(0.17) | 0.39(0.18) | 0.31(0.15) | 0.19(0.14) | 90(16.0%) | 229(40.7%) | 9(1.6%) | 234(41.6%) |
|  | **Never** | 0.41(0.19) | 0.37(0.17) | 0.51(0.20) | 0.41(0.17) | 0.28(0.16) | 587(22.9%) | 890(34.8%) | 57(2.2%) | 1025(40.1%) |

**Supplementary Table 1-2. Characteristics of participants, and the levels and changes of residential air pollution.** For each participant, we linked PM_2.5,_ NO_2_ and ozone data with participant location information. We considered the exposure window for PM_2.5,_ NO_2_ and ozone as the annual-average value in the death year for deceased individuals, and in the last interview year for those still alive at follow-up and those lost to follow-up. As NO_2_ data during 2001 to 2004 were unavailable, the NO_2_ concentration in 2000 was used as the last-year NO_2_ for participants whose end-up year was 2001 or 2002 and NO_2_ concentration in 2005 was used as the last-year NO_2_ for those with end-up year 2003 or 2004. Change of air pollutants was estimated by putting annual-average values of air pollutants over each participant’s follow-up period into a linear regression model and was defined as a significant increase or decrease if the regression coefficient was positive or negative, with its p-value less than 0.05. On the contrary, if the p-value was larger than 0.05, change was defined as non-significant. The mean concentration of last-year PM_2.5_, NO_2_ and ozone were 59.2 μg/m^3^ (SD:16.0 μg/m^3^), 20.2 μg/m^3^ (SD:14.6 μg/m^3^) and 74.2 μg/m^3^ (SD:6.8 μg/m^3^) for all 4,992 participants, respectively. Individuals who lived in areas with higher PM_2.5_ exposure level were aged 90-99 years, female, ethnic minorities, not co-habiting with a spouse, lived in inner-city, had higher household income, had previously worked in non-manual labor, received formal education less than one year, were former smokers, former alcohol consumers and who never exercised. Moreover, it was discovered that subgroup living with highest level of PM_2.5_ were ethnic minorities (72.3 μg/m^3^) and those from Tianjin (70.6 μg/m^3^) among the six megacities. Interestingly, NO_2_ exposure showed an opposite pattern to PM_2.5_ in terms of participants’ age, gender, marital status, education, smoking status, alcohol status and exercise. With respect to ozone exposure, we noticed that people living with highest level of ozone were ethnic minorities (75.7 μg/m^3^) and those from Tianjin (81.5 μg/m^3^). Furthermore, residential NO_2_ concentration of 1817 (36.4%) participants had no change, while 338 (6.8%) and 97 (1.9%) individuals lived with increasing and decreasing NO_2_ level respectively. Residential PM_2.5_ exposure showed an inverse U-shaped connection with time. As for last-year ozone level, there were 889 (17.8%) people living with stable ozone concentration, with 924 (18.5%) and 421(8.4%) participants had rising and declining ozone.

| **Characteristics** | | **PM_2.5_ (μg/m^3^)** | | |  | **NO_2_ (μg/m^3^)** | | | | |  | **O_3_ (μg/m^3^)** | | | | |
| --- | --- | --- | --- | --- | --- | --- | --- | --- | --- | --- | --- | --- | --- | --- | --- | --- |
|  |  | **Last-year PM_2.5_ (μg/m^3^)** | **Inverse U** | **Missing** |  | **Last-year NO_2_ (μg/m^3^)** | **No change** | **Increase** | **Decrease** | **Missing** |  | **Last-year O_3_ (μg/m^3^)** | **No change** | **Increase** | **Decrease** | **Missing** |
| **Total** | | 59.20(16.03) | 2976(59.62%) | 2016(40.38%) |  | 20.21(14.57) | 1817(36.4%) | 338(6.8%) | 97(1.9%) | 2740(54.9%) |  | 74.21(6.77) | 889(17.8%) | 92418.5%) | 421(8.4%) | 2758(55.2%) |
|  | **Range** | 22.30 to 107.30 |  |  |  | 0 to 60.09 |  |  |  |  |  | 37.12 to 100.99 |  |  |  |  |
|  | **Median (IQR)** | 55.90(46.90 to 69.50) |  |  |  | 15.07(8.57 to 30.34) |  |  |  |  |  | 74.32(69.48 to 78.61) |  |  |  |  |
| **Age, years** | |  |  |  |  |  |  |  |  |  |  |  |  |  |  |  |
| **Age group** | |  |  |  |  |  |  |  |  |  |  |  |  |  |  |  |
|  | **65-79 years** | 55.87(17.02) | 751(61.7%) | 466(38.3%) |  | 21.40(15.49) | 478(31.5%) | 64(4.2%) | 26(1.7%) | 649(42.8%) |  | 74.90(8.84) | 199(13.1%) | 275(18.1%) | 95(6.3%) | 648(42.7%) |
|  | **80-89 years** | 59.79(15.66) | 692(59.9%) | 463(40.1%) |  | 17.62(12.14) | 407(35.2%) | 107(9.3%) | 24(2.1%) | 617(53.4%) |  | 74.26(6.49) | 230(19.9%) | 178(15.4%) | 122(7.7%) | 625(54.1%) |
|  | **90-99 years** | 60.97(16.07) | 810(57.5%) | 599(42.5%) |  | 20.58(14.70) | 483(34.3%) | 98(7.0%) | 24(1.7%) | 804(57.1%) |  | 73.97(5.84) | 238(16.9%) | 254(18.0%) | 109(7.8%) | 808(57.3%) |
|  | **≥100 years** | 59.88(14.86) | 723(59.7%) | 488(40.3%) |  | 21.13(15.34) | 449(37.1%) | 69(5.7%) | 23(1.9%) | 670(55.3%) |  | 73.88(5.54) | 222(18.3%) | 217(17.9%) | 95(7.8%) | 677(55.9%) |
| **Sex** | |  |  |  |  |  |  |  |  |  |  |  |  |  |  |  |
|  | **Male** | 59.04(16.09) | 1241(59.0%) | 864(41.0%) |  | 20.68(14.62) | 725(34.4%) | 152(7.2%) | 35(1.7%) | 1193(56.7%) |  | 74.30(6.92) | 378(18.0%) | 376(17.9%) | 164(7.8%) | 1187(56.4%) |
|  | **Female** | 59.32(15.98) | 1735(60.1%) | 1152(39.9%) |  | 19.87(14.53) | 1092(37.8%) | 186(6.4%) | 62(2.1%) | 1547(53.6%) |  | 74.15(6.66) | 511(17.7%) | 548(19.0%) | 257(8.9%) | 1571(54.4%) |
| **Ethnicity** | |  |  |  |  |  |  |  |  |  |  |  |  |  |  |  |
|  | **Han Chinese** | 58.95(15.83) | 2916(59.7%) | 1969(40.3%) |  | 20.13(14.60) | 1784(36.5%) | 336(6.9%) | 97(2.0%) | 2668(54.6%) |  | 74.18(6.78) | 878(18.0%) | 906(18.5%) | 419(8.6%) | 2682(54.9%) |
|  | **Ethnic minorities** | 72.28(20.45) | 60(57.7%) | 47(45.2%) |  | 24.35(12.36) | 33(31.7%) | 2(1.9%) | 0(0) | 72(69.2%) |  | 75.66(5.87) | 11(10.6%) | 18(17.3%0 | 2(1.9%) | 76(73.1%) |
| **Marital status** | |  |  |  |  |  |  |  |  |  |  |  |  |  |  |  |
|  | **Married and living with spouse** | 57.52(16.77) | 846(60.5%) | 553(39.5%) |  | 21.23(14.97) | 519(37.1%) | 73(5.2%) | 29(2.1%) | 778(55.6%) |  | 74.49(8.05) | 218(15.6%) | 283(20.2%) | 114(8.1%) | 784(56.0%) |
|  | **Other** | 59.86(15.68) | 2130(59.3%) | 1463(40.7%) |  | 19.81(14.40) | 1298(36.1%) | 265(7.4%) | 68(1.9%) | 1962(54.6%) |  | 74.10(6.19) | 671(18.7%) | 640(17.8%) | 307(8.5%) | 1974(55.0%) |
| **Residence** | |  |  |  |  |  |  |  |  |  |  |  |  |  |  |  |
|  | **Inner-city** | 60.79(16.79) | 1971(59.9%) | 1318(40.1%) |  | 24.72(14.72) | 1218(37.0%) | 172(5.2%) | 75(2.3%) | 1824(55.5%) |  | 73.64(6.85) | 545(16.6%) | 627(19.1%) | 276(8.4%) | 1841(56.0%) |
|  | **Suburb** | 59.46(14.20) | 1005(59.0%) | 698(41.0%) |  | 12.45(10.45) | 599(35.2%) | 166(9.7%) | 22(1.3%) | 916(53.8%) |  | 75.21(6.50) | 344(20.2%) | 297(17.4%) | 145(8.5%) | 917(53.8%) |
| **Household income (RMB)** | |  |  |  |  |  |  |  |  |  |  |  |  |  |  |  |
|  | **<5000** | 58.79(13.72) | 1011(61.8%) | 625(38.2%) |  | 12.51(9.16) | 632(38.6%) | 155(9.5%) | 51(3.1%) | 798(48.8%) |  | 74.77(6.41) | 430(26.3%) | 205(12.5%) | 200(12.2%) | 801(49.0%) |
|  | **5000-15000** | 58.56(16.50) | 1096(60.5%) | 717(39.5%) |  | 26.72(16.15) | 659(36.3%) | 103(5.7%) | 24(1.3%) | 1027(56.6%) |  | 73.46(6.88) | 236(13.0%) | 428(23.6%） | 121(6.7%) | 1028(56.7%) |
|  | **>15000** | 63.87(19.46) | 512(56.8%) | 390(43.2%) |  | 24.05(14.18) | 317(35.1%) | 57(6.3%) | 9(1.0%) | 519(57.5%) |  | 74.18(7.84) | 143(15.9%) | 175(19.4%) | 58(6.4%) | 526(58.3%) |
| **City** | |  |  |  |  |  |  |  |  |  |  |  |  |  |  |  |
|  | **Beijing** | 81.61(16.48) | 480(58.7%) | 338(41.3%) |  | 24.69(9.04) | 182(22.2%) | 3(0.4%) | 0(0) | 633(77.4%) |  | 78.27(5.89) | 5(0.6%) | 118(14.4%) | 0(0) | 695(85.0%) |
|  | **Shanghai** | 45.54(4.67) | 791(60.2%) | 522(39.8%) |  | 36.72(15.38) | 492(37.5%) | 0(0) | 22(1.7%) | 799(60.9%) |  | 70.69(6.23) | 12(0.9%) | 522(39.8%) | 0(0) | 779(59.3%) |
|  | **Tianjin** | 70.63(10.09) | 201(56.1%) | 157(43.9%) |  | 22.20(8.86) | 109(30.4%) | 0(0) | 0(0) | 249(69.6%) |  | 81.45(5.62) | 8(2.2%) | 87(24.3%) | 0(0) | 263(73.5%) |
|  | **Chongqing** | 59.85(10.20) | 696(58.4%) | 496(41.6%) |  | 8.16(4.29) | 543(45.6%) | 102(8.6%) | 75(6.3%) | 472(39.6%) |  | 71.42(5.66) | 328(27.5%) | 2(0.2%) | 415(34.8%) | 447(37.5%) |
|  | **Chengdu** | 58.94(12.86) | 575(62.0%) | 353(38.0%) |  | 13.10(5.65) | 280(30.2%) | 233(25.1%) | 0(0) | 415(44.7%) |  | 75.72(5.47) | 481(51.8%） | 33(3.6%) | 6(0.6%) | 408(44.0%) |
|  | **Guangzhou** | 45.18(5.18) | 233(60.8%) | 150(39.2%) |  | 19.42(0.09) | 211(55.1%) | 0(0) | 0(0) | 172(44.9%) |  | 78.55(4.69) | 55(14.4%) | 162(42.3%) | 0(0) | 166(43.3%) |
| **Main occupation before 60 years of age** | |  |  |  |  |  |  |  |  |  |  |  |  |  |  |  |
|  | **Non-manual** | 61.26(18.56) | 315(55.7%) | 251(44.3%) |  | 28.57(14.92) | 179(31.6%) | 14(2.5%) | 6(1.6%) | 367(64.8%) |  | 74.49(7.88) | 53(9.4%) | 117(20.7%) | 29(5.1%) | 367(64.8%) |
|  | **Other** | 58.97(15.69) | 2659(60.2%) | 1761(39.8%) |  | 19.29(14.22) | 1637(37.0%) | 322(7.3%) | 91(2.1%) | 2370(53.6% |  | 74.18(6.63) | 834(18.9% | 806(18.2%) | 392(8.9% | 2388(54.0%) |
| **Education, years** | |  |  |  |  |  |  |  |  |  |  |  |  |  |  |  |
| **Education** | |  |  |  |  |  |  |  |  |  |  |  |  |  |  |  |
|  | **0 year** | 59.90(15.27) | 1599(60.0%) | 1068(40.0%) |  | 17.04(12.77) | 986(37.0%) | 224(8.4%) | 66(2.5%) | 1391(52.2%) |  | 74.32(6.17) | 553(20.7%) | 437(16.4%) | 267(10.0%) | 1410(52.9%) |
|  | **1-6 year** | 57.98(16.29) | 933(60.2%) | 618(39.8%) |  | 22.39(15.26) | 567(36.6%) | 96(6.2%) | 25(1.6%) | 863(55.6%) |  | 74.28(7.09) | 255(16.4%) | 320(20.6%) | 116(7.5%) | 860(55.4%) |
|  | **>6 years** | 59.27(18.37) | 420(57.1%) | 315(42.9%) |  | 28.76(15.76) | 247(33.6%) | 13(1.8%) | 6(0.8%) | 469(63.8%) |  | 73.67(8.30) | 73(9.9%) | 157(21.4%) | 34(4.6%) | 471(64.1%) |
| **Smoking status** | |  |  |  |  |  |  |  |  |  |  |  |  |  |  |  |
|  | **Current** | 58.97(15.40) | 575(60.5%) | 376(39.5%) |  | 16.76(12.48) | 358(37.6%) | 90(9.5%) | 15(1.6%) | 488(51.3%) |  | 74.66(7.09) | 235(24.7%) | 146(15.4%) | 87(9.1%0 | 483(50.8%) |
|  | **Former** | 60.63(16.44) | 498(61.4%) | 313(38.6%) |  | 20.71(13.53) | 269(33.2%) | 48(5.9%) | 18(2.2%) | 476(58.7%) |  | 75.02)6.36) | 133(16.4%) | 136(16.8%) | 63(7.8%) | 479(59.1%) |
|  | **Never** | 58.91(16.11) | 1901(58.9%) | 1325(41.1%) |  | 21.16(15.27) | 1190(36.9%) | 199(6.2%) | 64(2.0%) | 1772(54.9%) |  | 73.86(6.74) | 520(16.1%0 | 642(19.9%) | 271(8.4%) | 1793(55.6%) |
| **Alcohol status** | |  |  |  |  |  |  |  |  |  |  |  |  |  |  |  |
|  | **Current** | 58.89(15.66) | 568(59.6%) | 385(40.4%) |  | 17.32(13.42) | 344(36.1%) | 82(8.6%) | 21(2.2%) | 506(53.1%) |  | 74.15(7.02) | 209(21.9%) | 149(15.6%) | 96(10.1%) | 499(52.4%) |
|  | **Former** | 61.39(15.03) | 346(59.1%) | 239(40.9%) |  | 18.14(12.46) | 211(36.1%) | 44(7.5%) | 10(1.7%) | 320(54.7%) |  | 74.75(6.25) | 112(19.1%) | 87(14.9%) | 66(11.3%) | 320(54.7%) |
|  | **Never** | 58.90(16.29) | 2060(59.7%) | 1390(40.3%) |  | 21.45(15.08) | 1262(36.6%) | 211(6.1%) | 66(1.9%) | 1911(55.4%) |  | 74.13(6.78) | 567(16.4%) | 688(19.9%) | 259(7.5%) | 1936(56.1%) |
| **Exercise** | |  |  |  |  |  |  |  |  |  |  |  |  |  |  |  |
|  | **Current** | 60.69(17.17) | 1111(59.6%) | 753(40.4%) |  | 21.84(14.24) | 704(37.8%) | 78(4.2%) | 30(1.6%) | 1052(56.4%) |  | 74.48(7.39) | 294(15.8%) | 347(18.6%) | 169(9.1%) | 1054(56.5%) |
|  | **Former** | 63.06(17.03) | 328(58.4%) | 234(41.6%) |  | 24.79(14.50) | 197(35.1%) | 22(3.9%) | 6(1.1%) | 337(60.0%) |  | 74.30(6.43) | 71(12.6%) | 107(19.0%) | 34(6.0%) | 350(62.3%) |
|  | **Never** | 67.03(14.70) | 1534(59.9%) | 1025(40.1%) |  | 18.14(14.46) | 915(35.8%) | 237(9.3%) | 61(2.4%) | 1346(52.6%) |  | 74.01(6.37) | 523(20.4%) | 470(18.4%) | 218(8.5%0 | 1348(52.7%) |

**Supplementary Table 1-3. Characteristics of participants, and their distance to public facilities, and counts of public facilities in 1 and 5 km-radius buffers.** This table shows the accessibility to overall public facilities as well as to three main categories of public facilities, namely medicine-related facilities, sports and leisure service-related places and scenic spots-related places. We also specifically looked at 3a grade hospitals since they are closely related with health, though they are already included in medicine-related facilities. We discovered that some subgroups of participants lived nearer to public facilities and had more public facilities within 1 km and 5 km radius around each participant’s location, including individuals that were aged 65-79 years, male, ethnic minorities, married and living with spouse, lived in inner city, had higher household income, had previously worked in non-manual labor, received higher level education, while those who were current smokers, current alcohol consumers and never exercised had poorer accessibility to public facilities. We also observed that subgroup living with the easiest access to public facilities were those who received more than6 years of education and participants from Shanghai.

| **Characteristics** | | **Accessibility to public facilities (1km)** | **Accessibility to public facilities (5km)** | **Distance to the nearest facility (km)** | **Distance to the nearest facility (km)** | | | |  | **Counts of facilities (5 km)** | | | |
| --- | --- | --- | --- | --- | --- | --- | --- | --- | --- | --- | --- | --- | --- |
|  |  |  |  |  | **Medicine** | **Leisure** | **Landscape** | **3a hospitals** |  | **Medicine** | **Leisure** | **Landscape** | **3a hospitals** |
| **Total** |  | 2776(3489) | 54663(60639) | 0.22(0.36) | 0.59(0.81) | 0.63(1.08) | 0.87(1.08) | 8.36(11.34) |  | 1368(1389) | 1752(1964) | 566(708) | 72(97) |
|  | **Range** | 0 to 21421 | 2 to 210903 | 0.00 to 4.47 | 0.00 to 8.38 | 0.00 to 11.74 | 0.00 to 8.88 | 0.00 to 82.00 |  | 0 to 6044 | 0 to 6969 | 0 to 2631 | 0 to 392 |
|  | **Median (IQR)** | 1331(49 to 4314) | 24274(1752 to 95874) | 0.06(0.02 to 0.26) | 0.3(0.1 to 0.8) | 0.22(0.10 to 0.72) | 0.46(0.20 to 1.09) | 2.82(0.95 to 12.45) |  | 821(66 to 2438) | 100(65 to 3154) | 192(23 to 919) | 7(0 to 137) |
| **Age, years** | |  |  |  |  |  |  |  |  |  |  |  |  |
| **Age group** | |  |  |  |  |  |  |  |  |  |  |  |  |
|  | **65-79 years** | 3151(3652) | 63238(62120) | 0.18(0.32) | 0.50(0.79) | 0.54(1.02) | 0.75(1.01) | 6.94(10.34) |  | 567(1376) | 2010(2009) | 650(729) | 82(99) |
|  | **80-89 years** | 2433(3219) | 46413(55677) | 0.23(0.38) | 0.64(0.82) | 0.65(1.07) | 0.92(1.10) | 8.77(10.87) |  | 1272(1438) | 1451(1762) | 473(642) | 60(91） |
|  | **90-99 years** | 2558(3304) | 51402(59468) | 0.23(0.37) | 0.59(0.78) | 0.67(1.16) | 0.94(1.13) | 8.93(11.44) |  | 1255(1328) | 1675(1938) | 560(729) | 70(96) |
|  | **≥100 years** | 2982(3726) | 57710(63695) | 0.23(0.38) | 0.63(0.86) | 0.63(1.07) | 0.87(1.04) | 8.73(12.48) |  | 1392(1404) | 1868(2088) | 575(714) | 77(100) |
| **Sex** |  |  |  |  |  |  |  |  |  |  |  |  |  |
|  | **Male** | 2910(3518) | 57664(61346) | 0.20(0.35) | 0.57(0.82) | 0.59(1.06) | 0.82(1.03) | 7.76(10.72) |  | 1445(1423) | 1831(1976) | 595(718) | 76(98) |
|  | **Female** | 2679(3465) | 53475(60034) | 0.23(0.37) | 0.61().81) | 0.65(1.10) | 0.91(1.11) | 8.79(11.76) |  | 1312(1362) | 1694(1954) | 544(701) | 70(96) |
| **Ethnicity** | |  |  |  |  |  |  |  |  |  |  |  |  |
|  | **Han Chinese** | 2764(3501) | 54359(60837) | 0.22(0.37) | 0.60(0.82) | 0.63(1.09) | 0.88(1.08) | 8.44(11.37) |  | 1360(1392) | 1741(1970) | 554(700) | 71(96) |
|  | **Ethnic minorities** | 3326(2853) | 68547(49018) | 0.10(0.22) | 0.37(0.64) | 0.32(0.76) | 0.51(0.58) | 4.56(9.14) |  | 1750(1213) | 2221(1658) | 1076(872) | 153(120) |
| **Marital status** | |  |  |  |  |  |  |  |  |  |  |  |  |
|  | **Married and living with spouse** | 3090(3573) | 61826(62269) | 0.19(0.35) | 0.53(0.77) | 0.55(1.00) | 0.78(1.01) | 7.27(10.39) |  | 1543(1422) | 1965(2015) | 635(720) | 81(98) |
|  | **Other** | 2654(3448) | 51874(59769) | 0.23(0.37) | 0.62(0.83) | 0.65(1.11) | 0.91(1.10) | 8.78(11.67) |  | 1300(1371） | 1669(1938) | 529(702) | 69(96) |
| **Residence** | |  |  |  |  |  |  |  |  |  |  |  |  |
|  | **Inner-city** | 3792(3740) | 75423(60715) | 0.11(0.17) | 0.35(0.50) | 0.29(0.36) | 0.50(0.51) | 4.28(6.46) |  | 1887(1368) | 2404(1970) | 785(739) | 103(103) |
|  | **Suburb** | 816(1685) | 14571(34933) | 0.44(0.51) | 1.05(1.07) | 1.28(1.60) | 1.59(1.45) | 16.23(14.23) |  | 366(720) | 492(1185) | 142(378) | 12(41) |
| **Household income (RMB)** | |  |  |  |  |  |  |  |  |  |  |  |  |
|  | **<5000** | 1390(2380) | 25600(41700) | 0.35(0.47) | 0.88(1.00) | 1.01(1.44) | 1.25(1.25) | 13.0(13.2) |  | 784(1210) | 763(1260) | 236(453) | 25(60) |
|  | **5000-15000** | 3680(3920) | 73000(65700) | 0.16(0.26) | 0.46(0.68) | 0.45(0.80) | 0.69(0.93) | 6.21(9.99) |  | 1690(1370) | 2360(2140) | 747(758) | 98(103) |
|  | **>15000** | 3170(3350) | 67600(60200) | 0.14(0.22) | 0.42(0.61) | 0.40(0.73) | 0.65(0.89) | 5.88(9.29) |  | 1660(1320) | 2240(2000) | 722(734) | 92(98) |
| **City** |  |  |  |  |  |  |  |  |  |  |  |  |  |
|  | **Beijing** | 2456(2038) | 54333(37674) | 0.11(0.19) | 0.37(0.62) | 0.29(0.49) | 0.51(0.65) | 4.52(7.47) |  | 1426(928) | 1929(1358) | 1018(929) | 146(128) |
|  | **Tianjin** | 2536(2746) | 47477(419412) | 0.24(0.58) | 0.53(0.81) | 0.72(1.33) | 1.43(1.88) | 7.57(10.72) |  | 1169(965) | 1117(1016) | 331(341) | 39(39) |
|  | **Shanghai** | 5244(4329) | 105559(71344) | 0.08(0.14) | 0.32(0.46) | 0.24(0.28) | 0.56(0.88) | 4.44(7.23) |  | 2096(1309) | 3570(2292) | 971(728) | 126(94) |
|  | **Chongqing** | 1055(1996) | 14254(26005) | 0.47().51) | 1.10(1.14) | 1.41(1.66) | 1.41(1.23) | 16.02(15.65) |  | 521(896) | 435(846) | 95(165) | 6(15) |
|  | **Chengdu** | 3178(3803) | 60544(56777) | 0.22(0.23) | 0.61(0.56) | 0.51(0.53) | 0.86(0.68) | 9.47(9.07) |  | 1697(1523) | 1707(1569) | 695(671) | 73(84) |
|  | **Guangzhou** | 1706(2762) | 35194(47808) | 0.13(0.18) | 0.44(0.69) | 0.40(0.79) | 0.52(0.71) | 4.19(4.57) |  | 1318(1768) | 978(1298) | 235(304) | 29(54) |
| **Main occupation before 60 years of age** | |  |  |  |  |  |  |  |  |  |  |  |  |
|  | **Non-manual** | 4387(3970) | 86898(62975) | 0.10(0.20) | 0.33(0.60) | 0.30(0.73) | 0.51(0.88) | 3.85(7.92) |  | 2084(1374) | 2810(2034) | 919(760) | 123(104) |
|  | **Other** | 2570(3368) | 50549(59077) | 0.24(0.38) | 0.62(0.83) | 0.67(1.11) | 0.92(1.10) | 8.92(11.54) |  | 1277(1365) | 1616(1914) | 520(689) | 66(94) |
| **Education, years** | |  |  |  |  |  |  |  |  |  |  |  |  |
| **Education** | |  |  |  |  |  |  |  |  |  |  |  |  |
|  | **0 year** | 2093(3016) | 41512.2(53750) | 0.27(0.40) | 0.71(0.88) | 0.76(1.19) | 1.03(1.17) | 10.13(12.25) |  | 1088(1290) | 1326(1737) | 429(640) | 55(89) |
|  | **1-6 year** | 3095(3597) | 60531(62178) | 0.19().33) | 0.53(0.77) | 0.55(1.02) | 0.78(0.98) | 7.44(10.36) |  | 1509(1426) | 1929(2010) | 617(712) | 79(97) |
|  | **>6 years** | 4588(4103) | 90089(65527) | 0.10().20) | 0.32(0.53) | 0.30(0.63) | 0.49(0.76) | 3.96(7.76) |  | 2092(1364) | 2922(2125) | 950(776) | 123(104) |
| **Smoking status** | |  |  |  |  |  |  |  |  |  |  |  |  |
|  | **Current** | 2113(3155) | 41241(54856) | 0.25(0.38) | 0.69(0.90) | 0.72(1.17) | 0.97(1.07) | 10.08(12.18) |  | 1100(1345) | 1298(1749) | 413(622) | 51(86) |
|  | **Former** | 2858(3300) | 55515(56969) | 0.19(0.34) | 0.50(0.73) | 0.52(0.91) | 0.84(1.13) | 7.46(10.62) |  | 1485(1460) | 1743(1811) | 565(678) | 74(95) |
|  | **Never** | 2953(3607) | 58422(62612) | 0.22().36) | 0.58(0.80) | 0.63(1.10) | 0.85(1.06) | 9.06(11.16) |  | 1418(1375) | 1888(2041) | 611(733) | 78(99) |
| **Alcohol status** | |  |  |  |  |  |  |  |  |  |  |  |  |
|  | **Current** | 2277(3279) | 44405(57684) | 0.27(0.38) | 0.71(0.90) | 0.74(1.13) | 1.05(1.19) | 11.00(13.70) |  | 1141(1345) | 1422(1868) | 454(668) | 57(91) |
|  | **Former** | 2463(3045) | 48511(54642) | 0.21(0.34) | 0.57(0.75) | 0.60(0.97) | 0.82(0.90) | 7.99(10.12) |  | 1343(1454) | 1507(1723) | 478(645) | 62(92) |
|  | **Never** | 2969(3599) | 58567(62002) | 0.21(0.36) | 0.56(0.80) | 0.60(1.09) | 0.83(1.07) | 7.68(10.65) |  | 1436(1384) | 1885(2015) | 611(724) | 78(99) |
| **Exercise** | |  |  |  |  |  |  |  |  |  |  |  |  |
|  | **Current** | 3081(3506) | 61897(59603) | 0.17(0.29) | 0.50(0.73) | 0.47(0.82) | 0.72(0.94) | 6.33(9.30) |  | 1611(1435) | 1965(1915) | 651(725) | 84(100) |
|  | **Former** | 3468(3559) | 68736(60777) | 0.14(0.23) | 0.43(0.65) | 0.42(0.76) | 0.71(1.04) | 6.00(9.85) |  | 1715(1401) | 2181(1953) | 735(767) | 99(107) |
|  | **Never** | 2406(3421) | 46350(60180) | 0.27(0.42) | 0.70(0.89) | 0.78(1.27) | 1.02(1.16) | 10.34(12.53) |  | 1116(1304) | 1504(1971) | 466(666) | 58(89) |

**Supplementary Table 1-4. Characteristics of participants from different ethnic groups.** The table presents the disparity of demographic and socioeconomic characteristics, lifestyles, NDVI, air pollution and accessibility to public facilities between Han Chinese and their ethnic minority counterparts. Data is n (%) and mean (SD). The mean age of participants that were ethnic minority was 90.4 years, while that for Han Chinese was 87.8 years. In the ethnic minority groups, a greater proportion of people were not co-habiting with a spouse, lived in inner-city, had previously worked in non-manual labor, never smoked, never consumed alcohol and were former exerciser compared with Han Chinese. Additionally, ethnic minorities lived in areas with higher PM_2.5,_ NO_2_ and ozone, lower NDVI and easier excess to public facilities.

| **Characteristics** | | **Total** | **Han Chinese** | **Ethnic minorities** |
| --- | --- | --- | --- | --- |
| **Total** | | 4992(100%) | 4885(97.9%) | 107(2.1%) |
| **Age, years** | | 87.81(11.68) | 87.76(11.72) | 90.43(9.57) |
| **Age group** | |  |  |  |
|  | **65-79 years** | 1217(24.4%) | 1205(24.67%) | 12(11.21%) |
|  | **80-89 years** | 1155(23.1%) | 1128(23.09%) | 27(25.23%) |
|  | **90-99 years** | 1409(28.2%) | 1364(27.92%) | 45(42.06%) |
|  | **≥100 years** | 1211(24.2%) | 1188(24.31%) | 23(21.50%) |
| **Sex** | |  |  |  |
|  | **Male** | 2105(42.2%) | 2059(42.15%) | 46(42.99%) |
|  | **Female** | 2887(57.8%) | 2826(57.85%) | 61(57.01%) |
| **Marital status** | |  |  |  |
|  | **Married and living with spouse** | 1399(28.0%) | 1378(28.21%) | 21(19.63%) |
|  | **Other** | 3592(72.0%) | 3506(71.77%) | 86(80.38%) |
| **Residence** | |  |  |  |
|  | **Inner-city** | 3289(65.9%) | 3204(65.59%) | 85(79.44%) |
|  | **Suburb** | 1703(34.1%) | 1681(34.41%) | 22(20.56%) |
| **Household income (RMB)** | |  |  |  |
|  | **<5000** | 1620(32.45%) | 1615(33.06%) | 5(4.67%) |
|  | **5000-15000** | 1780(35.66%) | 1771(36.25%) | 9(8.41%) |
|  | **>15000** | 885(17.73%) | 885(18.12%) | 0(0) |
| **Main occupation before 60 years of age** | |  |  |  |
|  | **Non-manual** | 566(11.3%) | 552(11.30%) | 14(13.08%) |
|  | **Other** | 4420(88.5%) | 4328(88.60%) | 92(85.98%) |
| **Education, years** | | 2.78(8.27) | 2.80(4.18) | 2.75(4.06) |
| **Education** | |  |  |  |
|  | **0 year** | 2667(53.4%) | 2611(53.44%) | 56(52.34%) |
|  | **1-6 year** | 1551(31.1%) | 1517(31.05%) | 34(31.78%) |
|  | **>6 years** | 735(14.7) | 720(14.74%) | 15(14.02%) |
| **Smoking status** | |  |  |  |
|  | **Current** | 951(19.1%) | 936(19.16%) | 15(14.02%) |
|  | **Former** | 811(16.2%) | 796(16.29%) | 15(14.02) |
|  | **Never** | 3226(64.6%) | 3150(64.48%) | 76(71.03%) |
| **Alcohol status** | |  |  |  |
|  | **Current** | 953(19.1%) | 935(19.14%) | 18(16.82%) |
|  | **Former** | 585(11.7%) | 578(11.83%) | 7(6.54%) |
|  | **Never** | 3450(69.1%) | 3369(68.97%) | 81(75.70%) |
| **Exercise** | |  |  |  |
|  | **Current** | 1864(37.7%) | 1824(37.34%) | 40(37.38%) |
|  | **Former** | 562(11.2%) | 546(11.18%) | 16(14.95%) |
|  | **Never** | 2559(51.3%) | 2509(51.36%) | 50(46.73%) |
| **Last-year PM_2.5_ (μg/m^3^)** | | 59.20(16.03) | 58.95(15.83) | 72.28(20.45) |
| **Last-year NO_2_ (μg/m^3^)** | | 20.21(14.57) | 20.13(14.60) | 24.35(12.36) |
| **Last-year O_3_ (μg/m^3^)** | | 74.21(6.77) | 74.18(6.78) | 75.66(5.87) |
| **Cumulative annual NDVI (1250m)** | | 0.38(0.19) | 0.38(0.19) | 0.26(0.14) |
| **Accessibility to public facilities** | |  |  |  |
|  | **Counts of facilities (1 km)** | 2776(3489.19) | 2764(3501.11) | 3326(2852.56) |
|  | **Counts of facilities (5 km)** | 54663(60639.09) | 54359(60837.43) | 68547(49018.43) |
|  | **Distance to the nearest facility(km)** | 0.22(0.36) | 0.22(0.37) | 0.10(0.22) |

**Supplementary Table 2-1. HRs and 95% CIs for association between all-cause mortality and demographic, socioeconomic and lifestyle factors in age-sex-adjusted models.** The table presents the association of each covariate and mortality in a set of age- and sex-adjusted Cox regression models. When assessing the health effect of factors except age, the Cox models were adjusted for continuous age. As a well-known risk factor, age (continuous) had a hazard ratio (HR) (95% CI) of 1.08(1.08,1.09). Another well-studied predictor of health outcomes, gender, showed a HR (95%CI) of 0.83(0.77, 0.90), with female showing superior health outcomes. Furthermore, we noticed that the socioeconomic status of participants was an important determinant of successful aging in Chinese megacities. Individuals who were married and living with their spouse, who had previously worked in non-manual labor, who received more than 6 years of formal education as opposed to those without formal education, who resided in inner areas of the city as opposed to those living in suburb, and had 5000-15000-yuan annual household income as opposed to those with household income below 5000 yuan usually outlived their counterparts. Apart from that, people who were current smokers and alcohol consumers compared with former smokers and alcohol consumers, and those who currently exercised compared with those who never or formerly exercised had longer survival times. However, ethnicity showed no significant effect on mortality.

| **Characteristics** | **n** | **HR (95% CI)** | **P value** |
| --- | --- | --- | --- |
| **Continuous age** | 3818 | 1.082(1.077,1.086) | <0.001 |
| **Age group** |  |  |  |
| 65-79 years | 918 | Ref |  |
| 80-89 years | 911 | 3.759(3.285,4.302) | <0.001 |
| 90-99 years | 1057 | 7.422(6.489,8.490) | <0.001 |
| ≥100 years | 932 | 11.549(10.033,13.294) | <0.001 |
| **Sex** |  |  |  |
| Male | 1589 | Ref |  |
| Female | 2192 | 0.831(0.769,0.899) | <0.001 |
| **Ethnicity** |  |  |  |
| Han | 3711 | Ref |  |
| Other | 70 | 1.069(0.820,1.393) | 0.623 |
| **Education** |  |  |  |
| 0 year | 2093 | Ref |  |
| 1-6 years | 1191 | 0.963(0.876,1.059) | 0.435 |
| >6 years | 497 | 0.809(0.705,0.928) | 0.003 |
| **Occupation** |  |  |  |
| Non-manual | 394 | Ref |  |
| Other | 3387 | 1.179(1.025,1.357) | 0.021 |
| **Marriage** |  |  |  |
| Married and living with spouse | 1075 | Ref |  |
| Other | 2706 | 1.254(0.125,1.399) | <0.001 |
| **Continuous household income (RMB)** | 3818 | 1.000(1.000, 1.000) | 0.692 |
| **Household income (RMB)** |  |  |  |
| <5000 | 1445 | Ref |  |
| 5000-15000 | 1429 | 0.914(0.838,0.997) | 0.043 |
| >15000 | 591 | 0.961(0.858,1.077) | 0.494 |
| **Residence** |  |  |  |
| Inner-city | 2394 | Ref |  |
| Rural area | 1387 | 1.152(1.068,1.243) | <0.001 |
| **Smoking** |  |  |  |
| Current | 762 | Ref |  |
| Former | 1191 | 1.168(1.032,1.322) | 0.014 |
| Never | 497 | 0.993(0.892,.10107) | 0.903 |
| **Alcohol** |  |  |  |
| Current | 763 | Ref |  |
| Former | 455 | 1.173(1.026,1.340) | 0.019 |
| Never | 2563 | 1.085(0.984,1.197) | 0.103 |
| **Exercise** |  |  |  |
| Current | 1373 | Ref |  |
| Former | 414 | 1.466(1.291,1.665) | <0.001 |
| Never | 1994 | 1.383(1.269,1.506) | <0.001 |

**Supplementary Table 2-2. HRs and 95% CIs for association between all-cause mortality and nearby facilities in age-sex-adjusted models (n = 4,992).** Data is HRs and 95% CIs in a set of age-and sex-adjusted models that estimated the association between accessibility to public facilities and all-cause mortality. Shorter distances from public facilities to participants’ residences and more main health-related public facilities were related to lower risk of death: participants living 1 km further from medicine-related facilities, sports and leisure service-related places and scenic spots-related places had 8% (HR=1.08, 95% CI: 1.04,1.13), 5% (HR=1.05, 95% CI:1.02,1.08) and 7% (HR=1.07, 95% CI: 1.04,1.11) higher risks of mortality respectively; counts of the above three kinds of public facilities in 1 km-radius and 5 km-radius buffers also showed positive correlation with health outcomes.

| **Accessibility of public facilities** | | **HR (95% CI)** | **P value** |
| --- | --- | --- | --- |
| **Overall public facilities** | Distance to the nearest one | 1.157(1.057,1.266) | 0.002 |
|  | Count in 1 km-radius buffer (unit=50) | 1.000(0.999,1.000) | 0.030 |
|  | Count in 5 km-radius buffer (unit=150) | 1.000(1.000,1.000) | 0.001 |
| **Any medicine-related facilities** | Distance to the nearest one | 1.082(1.040,1.126) | <0.001 |
|  | Count in 1 km-radius buffer (unit=50) | 0.971(0.948,0.994) | 0.013 |
|  | Count in 5 km-radius buffer (unit=150) | 0.993(0.989,0.997) | 0.001 |
| **Any sports and leisure service-related** | Distance to the nearest one | 1.050(1.021,1.080) | <0.001 |
|  | Count in 1 km-radius buffer (unit=50) | 0.971(0.952,0.990) | 0.003 |
|  | Count in 5 km-radius buffer (unit=150) | 0.995(0.992,0.998) | <0.001 |
| **Any scenic spots-related** | Distance to the nearest one | 1.073(1.040,1.107) | <0.001 |
|  | Count in 1 km-radius buffer (unit=50) | 0.950(0.912,0.990) | 0.014 |
|  | Count in 5 km-radius buffer (unit=150) | 0.985(0.976,0.993) | <0.001 |
| **Any 3a grade hospitals** | Distance to the nearest one | 1.005(1.002,1.007) | 0.002 |
|  | Count in 1 km-radius buffer (unit=50) | 0.960(0.939,0.980) | <0.001 |
|  | Count in 5 km-radius buffer (unit=150) | 0.884(0.829,0.942) | <0.001 |

**Supplementary Table 2-3. HRs and 95% CIs for association between all-cause mortality and nearby facilities (by quartiles of counts in 5 km-radius buffer) in age-sex-adjusted models.** Data is HRs and 95% CIs in a set of age-and sex-adjusted models that estimated the association of three main categories of public facilities with mortality by quartile. Compared with the lowest quartile(count<=49), the highest quartile of medicine-related facilities (count > 2,312) had an HR of 0.83(95%CI:0.74,0.92). The highest quartile of sports and leisure service-related places (count >2,778) had an 16.9% lower risk of death than the lowest quartile (count <= 49). For scenic spot-related places, people in the highest quartile also had higher odds of survival (HR=0.82,95%CI:0.74,0.91) when using the lowest quartile(count<=18) as reference.

| **Counts of public facilities** | **n** | **HR (95% CI)** | **P value** |
| --- | --- | --- | --- |
| **Any medicine-related** |  |  |  |
| Q1 (<=49) | 958 | Ref |  |
| Q2 (49-588) | 954 | 0.914(0.828,1.010) | 0.077 |
| Q3 (588-2312) | 955 | 0.838(0.755,0.930) | <0.001 |
| Q4 (>2312) | 951 | 0.825(0.742,0.917) | <0.001 |
| **Any sports and leisure service-related** | |  |  |
| Q1 (<=49) | 956 | Ref |  |
| Q2 (49-422) | 954 | 0.948(0.858,1.046) | 0.287 |
| Q3 (422-2778) | 954 | 0.854(0.769,0.947) | 0.003 |
| Q4 (>2778) | 954 | 0.831(0.747,0.923) | <0.001 |
| **Any scenic spots-related** | |  |  |
| Q1 (<=18) | 962 | Ref |  |
| Q2 (18-124) | 949 | 0.930(0.843,1.027) | 0.154 |
| Q3 (124-729) | 954 | 0.841(0.758,0.933) | <0.001 |
| Q4 (>729) | 953 | 0.821(0.738,0.913) | <0.001 |

**Supplementary Table 2-4. HRs and 95% CIs for association of all-cause mortality with residential green space and air pollution in age- and sex-adjusted models.** Data presented in the table is HRs and 95% CIs in a set of age-and sex-adjusted models that estimated the association of NDVI and air pollutants as well as their change with time with mortality. The mortality HR for each 10 μg/m^3^ increase in PM_2.5_ was 1.04 (95%CI:1.02,1.07). Whereas, there was no appreciably discernible association of cumulative NDVI, last-year NO_2_ and ozone with health outcomes. We observed no effect of changes in greenness and air pollution exposure over time on mortality

| **Factors** | **n** | **HR (95% CI)** | **P value** |
| --- | --- | --- | --- |
| **NDVI (per 0.1-unit increase)** | 3732 | 1.018(0.997,1.038) | 0.089 |
| **NDVI Change** |  |  |  |
| No change | 547 | Ref |  |
| Increase | 788 | 0.908(0.822,1.003) | 0.056 |
| Decrease | 37 | 1.046(0.793,1.380) | 0.750 |
| **PM_2.5_ (10 μg/m^3^)** | 3781 | 1.044(1.021,1.067) | <0.001 |
| **PM_2.5_ Change** |  |  |  |
| No change | 1371 | Ref |  |
| Increase | 0 | NA |  |
| Decrease | 1 | 1.901(0.474,7.625) | 0.364 |
| **NO_2_ (10 μg/m^3^)** | 3675 | 0.978(0.951,1.005) | 0.103 |
| **NO_2_ Change** |  |  |  |
| No change | 887 | Ref |  |
| Increase | 195 | 1.148(1.000,1.318) | 0.051 |
| Decrease | 40 | 1.122(0.871,1.446) | 0.372 |
| **O_3_ (10 μg/m^3^)** | 3781 | 1.002(0.953,1.054) | 0.938 |
| **O_3_ Change** |  |  |  |
| No change | 508 | Ref |  |
| Increase | 388 | 0.923(0.815,1.045) | 0.203 |
| Decrease | 220 | 1.001(0.877,1.162) | 0.900 |

**Supplementary Table 3. Pearson-correlation coefficients and 95% CIs for association between residential nighttime light and covariates.** Data is Pearson correlation coefficients and their 95%CIs. We conducted Pearson correlation analysis to assess the relationship of nighttime light with demographic and socioeconomic characteristics, lifestyles, accessibility to public facilities, NDVI, air pollution. Results indicates that participants who were younger, female, ethnic minorities, who received higher level of education, who worked in manual labor before retirement, who were not co-habiting with a spouse, who had higher household income, who never smoked, consumed alcohol or exercised, who had easier accessibility to public facilities, who lived with less green space and higher air pollution generally lived in areas with higher nighttime light.

| **Factors** | **Pearson correlation coefficients(95%CI)** |
| --- | --- |
| **Age** | -0.05(-0.08, -0.03) |
| **Sex** | -0.03(-0.06,0) |
| Male |  |
| Female |  |
| **Ethnicity** | 0.07(0.04,0.10) |
| Han |  |
| Other |  |
| **Education** | 0.18(0.16,0.21) |
| **Occupation** | -0.18(-0.21, -0.15) |
| Non-manual |  |
| Other |  |
| **Marriage** | -0.07(-0.10, -0.05) |
| Married and living with spouse |  |
| Other |  |
| **Household income (RMB)** | 0.23(0.20,0.26) |
| <5000 |  |
| 5000-15000 |  |
| >15000 |  |
| **Residence** | -0.67(-0.68, -0.65) |
| Inner-city |  |
| Suburb |  |
| **Smoking** | 0.10(0.07,0.12) |
| Current |  |
| Former |  |
| Never |  |
| **Alcohol** | 0.13(0.10,0.15) |
| Current |  |
| Former |  |
| Never |  |
| **Exercise** | 0.13(0.10,0.15) |
| Current |  |
| Former |  |
| Never |  |
| **Overall accessibility to public facilities** |  |
| Total count of all facilities in 1 km-radius buffer | 0.62(0.61,0.64) |
| Total count of all facilities in 5 km-radius buffer | 0.75(0.73,0.76) |
| Distance to the nearest public facility | -0.59(-0.61, -0.57) |
| **Count in 5 km-radius buffers** |  |
| Any medicine-related facilities | 0.77(0.76,0.78) |
| Any sports and leisure service-related places | 0.74(0.73,0.75) |
| Any scenic spots-related places | 0.67(0.65,0.68) |
| **Accessibility to 3a grade hospitals** |  |
| Counts in 1 km-radius buffer | 0.35(0.33,0.38) |
| Counts in 5 km-radius buffer | 0.67(0.65,0.68) |
| Distance to the nearest 3a grade hospital | -0.71(-0.72, -0.69) |
| **NDVI (per 0.1-unit increase)** | -0.85(-0.86, -0.84) |
| **PM_2.5_ (10 μg/m^3^)** | 0.15(0.12,0.18) |
| **NO_2_ (10 μg/m^3^)** | 0.80(0.79,0.81) |
| **O_3_ (10 μg/m^3^)** | -0.04(-0.07, -0.01) |

**Supplementary Table 4. Prevalence, unadjusted risk ratios and Population Attributable Fractions (PAF) of mortality risk factors.** Risk ratios are unadjusted RRs of environmental, socioeconomic, and geographical risk factors for all-cause mortality. PAF indicates the fraction of mortality that could be prevented by eliminating certain risk factors from a population, which is calculated by the formula: PAF = P (RR − 1)/ (P [RR − 1] + 1), in which P is the population prevalence of a risk factor and RR is the risk ratio of that risk factor. As anticipated, SES, lifestyle and social resources were important determinants of health. Marriage (28.75%), occupation (28.42%), and exercise status (18.66%) were associated with the greatest fraction of attributable mortality. The count of medicine-related, leisure service-related, and scenic spots-related places in a 5 km-radius buffer showed a substantial association with mortality, with PAFs of 10.98%, 11.45% and 11.80%, respectively. However, environmental indicators made less contribution to death, among which, 11.63% deaths can be prevented by increasing nighttime light.

|  | **Risk Ratio** | **Prevalence (%)** | **PAF (%)** |
| --- | --- | --- | --- |
| **Age** |  |  |  |
| >99 years | 1.43 | 24.26 | 9.47 |
| <=99 years |  |  |  |
| **Sex** |  |  |  |
| Male | 0.94 | 42.17 | -2.54 |
| Female |  |  |  |
| **Ethnicity** |  |  |  |
| Ethnic minority | 1.04 | 2.08 | 0.09 |
| Han |  |  |  |
| **Education** |  |  |  |
| without formal education | 1.41 | 53.85 | 18.15 |
| received formal education |  |  |  |
| **Occupation** |  |  |  |
| Manual | 1.45 | 88.65 | 28.42 |
| Other |  |  |  |
| **Marriage** |  |  |  |
| Not married or not living with spouse | 1.56 | 71.97 | 28.75 |
| Other |  |  |  |
| **Household income (RMB)** |  |  |  |
| <7000 | 1.33 | 71.97 | 13.90 |
| >7000 |  |  |  |
| **Residence** |  |  |  |
| Suburb | 1.47 | 35.94 | 14.42 |
| Inner-city |  |  |  |
| **Smoking** |  |  |  |
| Current smoker | 1.00 | 19.07 | 0.01 |
| Non-smoker |  |  |  |
| **Alcohol** |  |  |  |
| Current drinker | 1.06 | 19.11 | 1.18 |
| Non-drinker |  |  |  |
| **Exercise** |  |  |  |
| Non-exerciser | 1.37 | 99.63 | 26.75 |
| Current exerciser |  |  |  |
| **Count of medicine-related facilities in 5 km-radius buffer** |  |  |  |
| The lowest quartile | 1.49 | 24.98 | 10.98 |
| other |  |  |  |
| **Count of sports and leisure service-related places in 5 km-radius buffer** |  |  |  |
| The lowest quartile | 1.53 | 24.58 | 11.45 |
| other |  |  |  |
| **Count of scenic spots-related places in 5 km-radius buffer** |  |  |  |
| The lowest quartile | 1.54 | 24.96 | 11.80 |
| other |  |  |  |
| **NDVI** |  |  |  |
| The lowest quartile | 0.81 | 24.99 | -4.92 |
| Other |  |  |  |
| **PM_2.5_** |  |  |  |
| The highest quartile | 1.09 | 24.94 | 2.11 |
| Other |  |  |  |
| **NO_2_** |  |  |  |
| The highest quartile | 0.81 | 25.01 | -4.90 |
| Other |  |  |  |
| **O_3_** |  |  |  |
| The highest quartile | 0.90 | 25.02 | -2.02 |
| Other |  |  |  |
| **Nighttime light** |  |  |  |
| The lowest quartile |  |  |  |
| Others | 0.12 | 24.94 | 11.63 |
